# A Novel Protein NAB1‐356 Encoded by circRNA circNAB1 Mitigates Atrial Fibrillation by Reducing Inflammation and Fibrosis

**DOI:** 10.1002/advs.202411959

**Published:** 2025-03-27

**Authors:** William W Du, Muhammad Rafiq, Hui Yuan, Xiangmin Li, Sheng Wang, Jun Wu, Jinfeng Wei, Ren‐Ke Li, Huiming Guo, Burton B Yang

**Affiliations:** ^1^ Sunnybrook Research Institute and Department of Laboratory Medicine and Pathobiology University of Toronto Toronto M4N3M5 Canada; ^2^ Department of Anesthesiology Beijing Anzhen Hospital Capital Medical University Beijing 100029 China; ^3^ Toronto General Research Institute University Health Network Department of Physiology University of Toronto Toronto M5G 2C4 Canada; ^4^ Department of Anesthesiology Guangdong Cardiovascular Institute Guangdong Provincial People's Hospital & Guangdong Academy of Medical Sciences Guangzhou Guangdong 510080 China

**Keywords:** cardiac fibrosis, cardiac remodeling, circNAB1, circular RNA

## Abstract

Atrial fibrillation (AF) is a common arrhythmia with irregular atrial electrical activity. Circular RNAs (circRNAs) are key regulators in tissue homeostasis, yet their role in AF remains unclear. Here, we investigated the expression and function of circNAB1 in AF using high‐throughput sequencing and functional assays in circNAB1 transgenic mice. We identified circNAB1 as a significantly downregulated circRNA in AF patient specimens. Silencing circNAB1 promoted collagen deposition and inflammation, whereas overexpression reduces atrial fibrosis and AF susceptibility in mice, consistent with results observed in human atrial tissues. Mechanistically, circNAB1 translates into a novel protein, NAB1‐356, which is highly expressed in human cardiac hypertrophy. NAB1‐356 interacts with EGR1 as NAB1 does, decreasing fibrosis and inflammation in the atrium. Furthermore, NAB1‐356 also regulates transcription factor Runx1 and Gadd45b transcription, exerting regulatory effects on cytokine expression and fibrosis. Targeting EGR1, Gadd45b, and Runx1 by circNAB1 or siRNAs attenuate AF incidence and cardiac remodeling, suggesting potential therapeutic strategies for AF management. Delivery of circNAB1 improves AF conditions in LKB1 knockout mice, further highlighting its anti‐arrhythmic potential. Our findings elucidate the mechanistic role of circNAB1 in AF pathogenesis and suggest its therapeutic implications for cardiac remodeling‐associated disorders.

## Graphical abstract

The circRNA circNAB1 is downregulated in AF patient. Silencing circNAB1 promotes collagen deposition and inflammation, whereas overexpression reduces AF susceptibility. circNAB1 encodes the protein NAB1 356 that interacts with EGR1 to mitigate fibrosis and inflammation. NAB1 356 also regulates Runx1 and Gadd45b transcription, influencing cytokine expression and fibrosis. Our findings highlight circNAB1's mechanistic role in AF and its therapeutic potential.

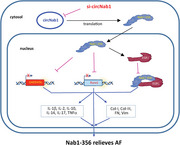

## 1 Introduction

Atrial fibrillation (AF) is a prevalent cardiac arrhythmia characterized by abnormal heart rhythm, wherein the atrial chambers beat irregularly.^[^ ^1^ ^]^ In AF, short periods of irregular beating are followed by longer irregular rhythms. While patients may remain asymptomatic, others may experience symptoms such as rapid, irregular, or skipped heartbeats, particularly noticeable after physical exertion. Additionally, chest pain may occur due to increased demand for oxygen surpassing the supply of the heart. AF can also lead to complications including congestive swelling, transient ischemic attack, heart failure, and stroke.^[^ ^2^ ^]^ High blood pressure is the most common risk factor causing AF, with other risk factors including cardiomyopathy, mitral stenosis, and mitral regurgitation.^[^ ^3^, ^4^ ^]^ These factors induce atrial dilation, hypertrophy, tissue remodeling, and fibrosis.^[^ ^5^ ^]^ Cardiac remodeling and fibrosis alter the structure, shape, size, and function of the heart, with progressive changes observed primarily in AF. Remodeling may induce ventricular hypertrophy and dilation, reducing contractile strength and stroke volume, and leading to myocardial fibrosis. This weakened tissue may fail to withstand the pressure and volume load, leading to chamber dilatation and impaired function. Thus, pressure overload exacerbates the severity of AF and may lead to cardiovascular death or progressive disability due to heart failure. To model pressure overload‐induced cardiac remodeling and fibrosis, we utilized an animal model of transverse aortic constriction (TAC), which demonstrated increased collagen deposition and decreased cardiomyocyte viability.^[^ ^6^, ^7^ ^]^

NAB1 (NGFI‐A Binding Protein 1) is a member of the corepressor family for early growth response (EGR) transcription factors. Initially identified through the yeast two‐hybrid system, NAB1 was found to interact with EGR1 and inhibit transcription activity of EGR members.^[^ ^8^, ^9^ ^]^ NAB1 belongs to the evolutionarily conserved family of corepressors and specifically represses transcription mediated by members of the NGFI‐A family. While NAB1 does not impede DNA binding or nuclear localization, it directly binds to the repression domain of transcription factors such as EGR1, EGR2, and EGR3 to repress DNA promoter activity.^[^ ^9^, ^10^ ^]^ Mutations affecting this domain led to decreased repression of EGR2 through NAB1.^[^ ^11^ ^]^ In cardiac physiology, NAB1 exhibits upregulation in conditions of cardiac hypertrophy and heart failure. NAB1 is highly expressed in cardiac myocytes and regulates cardiomyocyte growth through interaction with EGRs.^[^ ^12^ ^]^ Transgenic mice expressing NAB1 displays an ability to inhibit pathological cardiac growth by repressing EGR activity in vivo.^[^ ^12^ ^]^ Overexpression of NAB1 and deletion of EGR1 have been shown to protect the heart from pathological cardiac hypertrophy and fibrosis.^[^ ^12^ ^]^ Interestingly, overexpression of NAB1 does not influence physiological cardiac growth response. NAB1 transgenic mice exhibit a cardiac growth response similar to wild‐type mice under physiological conditions, indicating a specific role for NAB1 in pathological but not physiological hypertrophy.

Circular RNAs are non‐coding single stranded RNAs generated from genomic transcripts with covalently closed loops at the 5′ and 3′ ends. Circularization occurs through a splice donor site joined to a splice acceptor site upstream of the primary transcript.^[^ ^13^ ^]^ Expression of some circular RNAs is specific to cell type and developmental stage implying specific functions of these circular RNAs.^[^ ^14^, ^15^, ^16^, ^17^ ^]^ Recent studies have highlighted the role of circular RNAs as templates for protein translation, further expanding our understanding of their functional diversity.^[^ ^18^, ^19^, ^20^, ^21^, ^22^ ^]^ In the context of cardiac biology, non‐coding RNAs, including circular RNAs, have been increasingly implicated in pathological cardiac remodeling.^[^ ^23^, ^24^, ^25^, ^26^ ^]^ High‐throughput RNA sequencing of specimens obtained from fibrotic myocardial tissue has been instrumental in elucidating the differential expression patterns of circular RNAs, offering valuable insights into their potential roles in cardiac pathophysiology.

## 2 Results

### 2.1 circNAB1 Expression in AF

**Figure 1:**
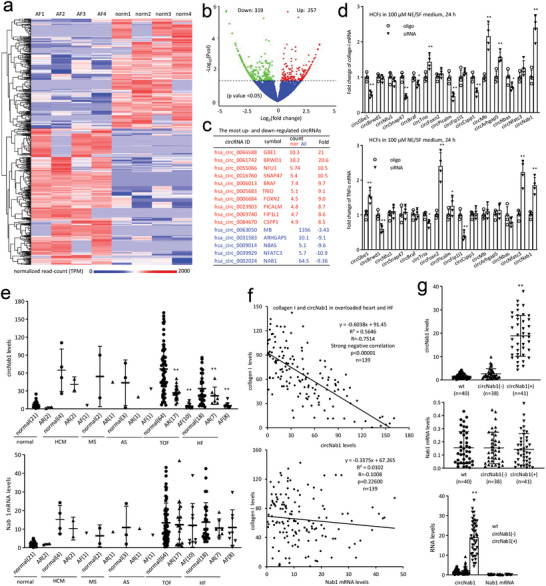
Expression of circNAB1 in AF. a) Heatmap illustrating differentially expressed circular RNA profiles in AF patients relative to normal atrial tissues. The color scale represents number of normalized read‐counts (range: 0–2000). b) Volcano plot displaying circRNAs detected by RNA sequencing, showing differential expression at *p* values *<* *0.05*. c) List of the most up‐ and down‐regulated 15 circRNAs with the highest abundance. d) Human cardiac fibroblasts (HCFs) were transfected with siRNAs against indicated circular RNAs and cultured in serum‐free DMEM/F12 medium with 100 µm norepinephrine (NE) for 24 h. RT‐PCR showed expression of collagen‐I (upper) and TNFα (lower) in the transfected cells (*p < 0.05, **p < 0.01 versus oligo; *n = 6*). e) Human heart tissues were subjected to RT‐PCR, showing that expression of both circNAB1 and linear NAB mRNA in normal rhythm, arrhythmia (AR), and atrial fibrillation cases within normal heart, hypertrophic cardiomyopathy (HCM), mitral stenosis (MS), aortic stenosis (AS), Tetralogy of Fallot (TOF), and heart failure (HF) samples. AF patients displayed significantly decreased circNAB1 levels compared with normal rhythm cases. The arrhythmia (AR) group included supraventricular tachycardia (SVT), premature atrial contractions (PAC), premature ventricular contractions (PVC), Atrioventricular node block (AV node block), and ventricular conduction block (*p < 0.05, **p < 0.01 versus normal; case numbers labeled in the figures). f) Correlation analysis of collagen‐I with circNAB1 (upper) and NAB1 (lower) in 139 heart disease specimens. Pearson correlation was performed to evaluate the association. Trend line, R^2^ and p value were labeled in the figures. g) RNAs isolated from wildtype (wt), circNAB1(‐), and circNAB1(+) mice was subject RT‐PCR. circNAB1(+) mouse hearts expressed high levels of circNAB1, and unchanged NAB1 mRNA (**p < 0.01 versus wt; n was labeled in the figures).

We performed high‐throughput circular RNA sequencing in the auricles of patients clinically diagnosed with atrial fibrillation (AF) and non‐AF (normal) cases, using donor hearts intended for transplantation. The four AF cases were diagnosed with persistent AF lasting for more than a week and exhibited typical AF electrocardiograms (ECGs), characterized by absent P waves and an irregularly irregular QRS complex before surgery. The four non‐AF samples were collected from patients with no AF history and presented with normal ECG before surgery. A total of 46667 circRNAs were identified in these samples, by at least two reads spanning a head‐to‐tail splice junction in each sample. Among them, 13516 were already included in circBase.^[^ ^27^ ^]^ Compared to normal auricle, the numbers of differentially expressed circular RNAs at a significant twofold cut‐off were 277, 139 up regulated, 138 down regulated (**Figure** **1**a; Figure S1a; Table S1, Supporting Information). At a fourfold cut‐off, there are 23 up and 13 down regulated. At a eightfold cut‐off, there were 10 up and 4 down regulated. The one that was expressed at the highest level was also included in this group making a total of 15 for the pre‐screening assay (siRNA assay) (Figure 1b,c; Figure S1b,c, Supporting Information). We examined the effects of the listed circular RNAs on collagen deposition that is associated with AF development by designing siRNAs targeting the junction sequence of each circular RNA. In all siRNAs tested, only silencing circNAB1 significantly increased collagen deposition and cytokine TNF‐α expression (Figure 1d).

We collected human specimens from patients with heart diseases associated with pressure overload (PO, n = 106), including Tetralogy of Fallot (TOF, n = 91), hypertrophic cardiomyopathy (HCM, n = 7), aortic stenosis (AS, n = 5), and mitral stenosis (MS, n = 3), all with a normal left ventricular ejection fraction (LVEF) above 40%. Heart samples were also obtained from heart failure cases (HF, n = 33) selected from individuals with PO heart diseases exhibiting reduced ejection fraction (HFrEF), with LVEF below 40%. We also collected 23 normal heart samples from individuals without detectable cardiovascular diseases who died from non‐cardiac related causes. All cases were classified into subgroups: normal rhythm, arrhythmia (AR), and AF. The AR group included supraventricular tachycardia (SVT), premature atrial contractions (PAC), premature ventricular contractions (PVC), atrioventricular node block (AV node block) and ventricular conduction block. The no symptom paroxysmal SVT (PSVT) cases were excluded. All the occasional PAC/PVC and less than 5 PACs/PVCs per minute on ECG or 30 PACs/PVCs per hour in during ambulatory monitoring cases were excluded. The AF group only included persistent AF lasting more than a week. All these heart samples were obtained from heart donations or surgeries, and heart rhythm information was sourced from patient case histories.

We measured levels of circNAB1 by real‐time PCR (primers listed in Major Resources Table) and detected significantly lower levels of circNAB1 in patients with AF compared to those with normal rhythm, a difference not observed for the linear full‐length NBA1 mRNA (Figure 1e). Since collagens are markers of tissue fibrosis, which is a key factor in AF, we measured collagen‐I levels in heart disease specimens and observed a negative correlation between levels of collagen and circNAB1, but not NAB1 mRNA (Figure 1f).

We generated human circNAB1 expression construct (Figure S2a, Supporting Information) and transfected it into the mouse atrial cardiomyocyte cell line HL‐1. We amplified the junction of circNAB1 and confirmed it by DNA sequencing (Figure S2b, Supporting Information). In human cardiomyocyte AC16 cells, this construct produced a transcript that was translocated to the cytoplasm (Figure S2c, Supporting Information) and was resistant to RNAse R digestion (Figure S2d, Supporting Information). The human circNAB1 expression construct was then placed under the control of the α‐myosin heavy chain promoter and used to generate transgenic mice expressing circNAB1. We confirmed that circNAB1‐transgenic mice produced significantly higher levels of circNAB1 in the heart compared to wildtype and litter‐matched negative mice (Figure 1g).

### 2.2 Improved Cardiac Functioning in Transgenic Mice Expressing circNAB1

**Figure 2:**
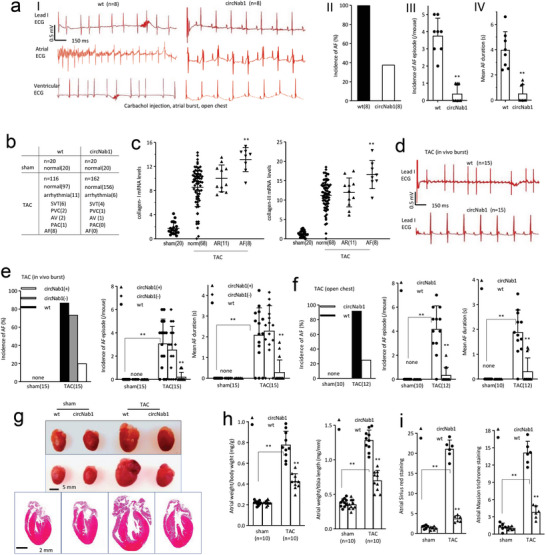
Functions of circNAB1. a) 8‐week‐old wt and circNAB1(+) mice were intraperitoneally administrated carbamyl choline (Carbachol, 50 ng g^−1^). After 5–10 min, mice were processed to open chest programmed electrical stimulation of right atrium, with electrocardiogram (ECG) recording lead I, atrial and ventricular electrical signals. Typical images recorded lead I, atrial and ventricular ECG after termination of the burst of atrial stimulation (I). Transgenic expression of circNAB1 decreased AF incidence (II), AF episode (III), and AF duration (IV). b) The incidence of AF in the mouse model was assessed in 8‐week‐old wt and circNAB1(+) mice subjected to sham and pressure overload (PO) surgery by transverse aortic constriction (TAC) for 12 weeks. The tested mice were anesthetized with 2% isoflurane, and surface ECG was recorded using electrodes configured in a lead‐I configuration. In the tested 116 wt mice, arrhythmia was detected in 11 mice, 8 mice developed AF. While in 162 circNAB1(+) mice, only 6 mice developed arrhythmia, no AF was detected. The occurrence rate of both arrhythmia and AF of the circNAB1(+) mice was much lower than wt (Fisher's exact test, two sided, *p<0.001*; n was labeled in the figures). Anaesthetized with 2% isoflurane, most mice showed normal with rates from 350–800 beats per minute (bpm). The arrythmia group included individuals with supraventricular tachycardia (SVT), premature atrial contractions (PAC), premature ventricular contractions (PVC), Atrioventricular node block (AV node block). AF ECG shows no visible P waves and an irregularly irregular QRS complex. Supraventricular tachycardia (SVT) is defined by a narrow QRS at a heart rate >800 bpm. Premature ventricular contractions (PVCs) are extra heartbeats that begin in ventricles, with wide abnormally shaped QRS complexes that occur earlier than expected. Premature atrial contractions (PACs) are premature heartbeats that occur in atria, an early P wave that has a different shape than a normal P wave. More than 10 PVCs or PACs/min on the routine ECG were included in the group as PVCs or PACs. AV node block is indicated by a P wave that is not followed by a QRS wave or a prolonged PR interval greater than 0.04 s. c) RT‐PCR analysis of atrial tissues from TAC wt mice showing significant increase in circNAB1 in ECG‐normal TAC mice, but not in AF mice. PCR also demonstrated that AF mouse atrial tissues expressed high levels of collagen‐I and collagen‐III (**p < 0.01 versus normal; n was labeled in the figures). d) In vivo transesophageal programmed electrical stimulation of left atrium with surface ECG (lead I) analysis in wt and circNAB1(+) mice subjected to TAC for 12 weeks. Representative ECG images recorded lead I after termination of the burst of atrial stimulation. e) ECG analysis showing lower incidence of AF, fewer AF episodes, and shorter mean AF duration in circNAB1(+) mice compared to wt mice during 3 series of bursts (**p < 0.01 versus wt; *n = 15*). f) ECG analysis of wt and circNAB1(+) mice subjected to TAC for 12 weeks, followed by open chest programmed electrical stimulation of the right atrium. Lower incidence of AF, fewer AF episodes, and shorter mean AF duration were observed in circNAB1(+) mice compared to wt mice during 3 series of bursts (**p < 0.01 versus wt; *n = 12*). g) Whole‐mount heart images (upper) and HE‐stained heart sections (lower) showing atrial sizes of circNAB1(+) mice after TAC. h) Reduced ratio of atrial weight/body weight or tibia in circNAB1(+) mice compared to wt mice after TAC (**p < 0.01 versus wt; *n = 10*). i) ImageJ analysis showing decreased Masson's trichrome and Sirius red staining in atriums of circNAB1(+) mice compared to wt mice after TAC (**p < 0.01 versus wt; *n = 6*).

Eight‐week‐old circNAB1(+) and wildtype (wt) mice were intraperitoneally administrated with carbamyl choline and processed to open chest programmed electrical stimulation of right atrium. ECG analysis detected significantly lower incidence, fewer episodes, and shorter duration of atrial fibrillation (AF) in circNAB1(+) mice (**Figure** **2**a). Similarly, we examined spontaneous incidence of AF in the circNAB1‐transgenic mice by surface ECG record using electrodes in a lead‐I configuration, 12 weeks of pressure overload induced by transverse aortic constriction (TAC). circNAB1(+) mice showed reduced incidence of both arrhythmia and AF compared to wt mice (Figure 2b; Figure S3a, Supporting Information).

Since cardiac fibrosis could initiate AF, and collagens are markers of fibrosis, we investigated the relationship between circNAB1 expression and collagen deposition in the mouse atrium. RT‐PCR analysis of atrial tissues from TAC‐induced wt mice revealed significant elevated levels of collagen‐I and collagen‐III in AF mouse atrial tissues (Figure 2c). PCR analysis showed an increase in circNAB1 expression, whereas its levels remained unchanged in the AF mice (Figure S3b, Supporting Information). Correlation analysis of collagen‐I and collagen‐III with circNAB1 in 87 mouse atrial specimens after TAC indicated a negative association, as assessed by Pearson correlation (Figure S3c, Supporting Information).

Additionally, in vivo transesophageal programmed electrical stimulation of the left atrium in TAC‐induced 8‐week‐old wt, circNAB1(‐) and circNAB1(+) mice demonstrated improvement in ECG measurement (Figure 2d), resulted in lower incidence, fewer episodes, and shorter duration of AF in circNAB1(+) mice compared to wt and circNAB1(‐) mice (Figure 2e). Similarly, programmed electrical stimulation of the right atrium in TAC‐induced mouse hearts under Langendorff‐perfusion revealed reduced AF susceptibility in circNAB1(+) mice (Figure S4a,b, Supporting Information).

To corroborate the role of circNAB1 in fibrosis, we examined the effect of TAC‐induced AF on cardiac fibrosis affected by circNAB1 expression. Following TAC, mice underwent open‐chest programmed electrical stimulation of the right atrium, with electrocardiogram (ECG) recording lead I, atrial and ventricular electrical signals. Notably, circNAB1(+) mice exhibited a significantly lower incidence of atrial fibrillation (AF), fewer AF episodes, and shorter mean AF duration compared to wt mice across three series of bursts (Figure 2f; Figure S4c, Supporting Information). Whole‐mount heart images and HE stained sections revealed reduced atrial sizes in circNAB1(+) mice post‐TAC (Figure 2g). A diminished ratio of atrial weight to body weight or tibia length was detected in circNAB1(+) mice compared to wt counterparts after TAC (Figure 2h). Furthermore, Masson's trichrome and Sirius red staining revealed notable reductions in atrial fibrosis in circNAB1(+) mice, as supported by ImageJ analysis, highlighting the therapeutic potential of circNAB1 in attenuating atrial fibrosis‐associated pathologies (Figure 2i; Figure S4d, Supporting Information).

### 2.3 Translation of circNAB1

**Figure 3:**
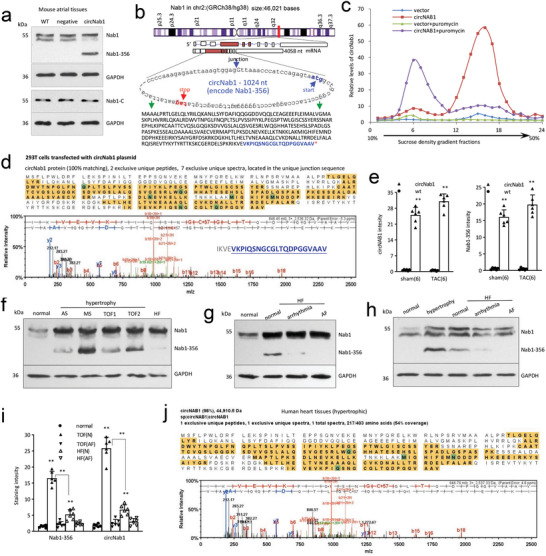
Translation of circNAB1. a) Mouse atrial tissues were lysed and subjected to western blot probing with antibody against the N‐terminus of NAB1. A significant NAB1‐356 band was detected in circNAB1(+) mice. b) Gene structure and amino acids translated from circNAB1. Due to circularization, a 21‐amino‐acid motif (blue) is added to the major core peptide (green, 335 amino acids). c) Lysates prepared from vector‐ and circNAB1‐transfected 293T cells were subjected to sucrose gradient assays. The corresponding separated RNAs were subjected to RT‐PCR, showing that circNAB1 transfected cells expressed higher levels of circNAB1 than vector control, with most circNAB1 accumulating in polysome fractions, which shifted to lighter polysomes after puromycin treatment. d) HEK 293T cells transiently transfected with the human circNAB1 construct were processed for immunoprecipitation with antibody against NAB1 (N‐T), followed by mass spectrometry analysis. Photographs showed the spectrum analysis of the detected NAB1‐356 containing the unique circNAB1 junction amino acids. e) In situ hybridization and immunofluorescence staining followed by ImageJ analysis showed high levels of both circNAB1 and NAB1‐356 in circNAB1(+) mouse atriums (**p < 0.01 versus wt; *n = 6*). f) Human atrial tissues were lysed and subjected to western blot probing with antibody against the N‐terminus of NAB1. A significant NAB1‐356 band was detected in hypertrophy (AS, MS, and TOF), and a weak NAB1‐356 band was also detected in HF. g) Western blot detected a NAB1‐356 band in HF with normal ECG, but not in the atrium with AF. h) Western blot detected a NAB1‐356 band in HF with normal ECG, but not in the atrium with AF. i) In situ hybridization and immunofluorescence staining followed by ImageJ analysis showed both circNAB1 and NAB1‐356 expressed at high levels in human atriums with no AF (**p < 0.01 versus normal; *n = 6*). j) Human atrial tissues from hypertrophy patients with no‐AF were lysed and subjected to immunoprecipitation with antibody against the N‐terminus of NAB1 (N‐T). Pull‐down products were processed to mass spectrometry. Photographs show the spectrum analysis of the detected NAB1‐356 containing the unique circNAB1 junction amino acids.

We examined whether ectopic circNAB1 affected expression of endogenous NAB1 using atrial tissues for Western blotting. We detected an extra smaller protein band (40 kDa) in the circNAB1‐transgenic mice, using a N‐terminus NAB1 antibody (**Figure** **3**a). Computational algorithm revealed potential translation of a 356 amino acid peptide, annotated as NAB1‐356 (Figure 3b). It showed that a 21‐amino‐acid motif (VKPIQSNGCGLTQDPGGVAAV) was added to this peptide because of back‐splicing. To confirm translation of circNAB1, we expressed circNAB1 in HEK293T cells, harvested cell lysates and performed sucrose gradient assays. The corresponding separated RNAs were subjected to RT‐PCR measuring circNAB1. The circNAB1‐transfected cells showed higher levels of circNAB1 interacted with polysome fractions than the vector control, but the peak shifted to the lighter polysome fractions after puromycin treatment, suggesting ongoing translation (Figure 3c). Sucrose gradient assays showed the accumulation of circNAB1 in the polysomes suggesting translation of circNAB1 (Figure S5a, Supporting Information). The lysates were also subjected to immunoprecipitation with the antibody against the N‐terminus of NAB1 (N‐T). The precipitated proteins were subjected to mass spectrometry analysis. The peptides detected covered most of protein sequence encoded by circNAB1 (Figure 3d). Specially, the unique protein sequence encoded by the back‐splicing nucleotide sequence was detected, confirming translation of circNAB1. In situ hybridization and immunofluorescence staining using circNAB1(+) mouse models revealed significantly higher levels of both circNAB1 and NAB1‐356 in cardiomyocytes of circNAB1(+) mouse atriums relative to wt mice (Figure 3e). While we detected circNAB1 mainly in cytosol, NAB1‐356 appeared mainly in nuclei (Figure S5b, Supporting Information).

We then performed Western blotting to examine human atrial tissues and detected the NAB1‐356 band in hypertrophy conditions (AS, MS, and TOF), with a weaker band detected in HF (Figure 3f). Intriguingly, NAB1‐356 was absent in atriums with atrial fibrillation (AF), suggesting a disease‐specific expression pattern (Figure 3g,h). In situ hybridization and immunofluorescence staining confirmed expression of circNAB1 and NAB1‐356 in both cardiomyocytes and cardiac fibroblasts of human atriums affected by TOF and HF (Figure S6, Supporting Information). Notably, circNAB1 was predominantly expressed in the cytosol, whereas NAB1‐356 localized to the nuclei. ImageJ analysis demonstrated significantly higher levels of both circNAB1 and NAB1‐356 in human atriums without AF compared to those with AF, suggesting a potential correlation between their expression levels and AF status (Figure 3i). We precipitated NAB1‐356 with the anti‐NAB1 antibody for mass spectrometry analysis and confirmed the presence of NAB1‐356 containing unique circNAB1 junction amino acids in human hypertrophic atriums without AF status (Figure 3j). These findings underscore the importance of circNAB1 and NAB1‐356 in AF pathogenesis and offer insights into their potential diagnostic and therapeutic implications. It highlighted the translational potential of circNAB1 and its association with cardiac pathologies, providing valuable insights into its mechanistic role in cardiac health and disease.

### 2.4 Mediating circNAB1 Functions by Runx1 and Gadd45b

**Figure 4:**
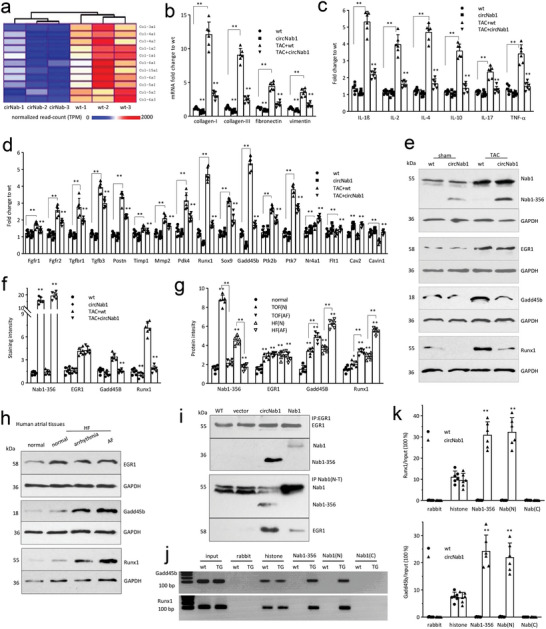
Signaling of Runx1 and Gadd45b. a) Heatmap illustrated collagen expression in circNAB1(+) mouse atriums relative to wt, 4 weeks after TAC. The color scale represents number of normalized read‐counts (range: 0–2000). b) PCR showed that circNAB1(+) mouse atriums expressed low levels of fibrosis markers, including collagen‐I, collagen‐III, fibronectin and vimentin, after TAC (**p < 0.01 versus wt; *n = 6*). c) PCR showed that circNAB1(+) mouse atriums expressed low levels of cytokine IL‐1β, Il‐2, Il‐4, Il‐10, Il‐17, and TNF‐α after TAC (**p < 0.01 versus wt; *n = 6*). d) PCR showed that circNAB1(+) mouse atriums expressed significantly low levels of Gadd45b and Runx1 after TAC (**p < 0.01 versus wt; *n = 6*). e) Western blot showed that TAC induced Gadd45b and Runx1 expression in mouse atriums, which could be prevented by expression of circNAB1. f) IHC staining images showed expression of EGR, Gadd45b, Runx1, and NAB1‐356 in wt and circNAB1(+) mouse atrium slides with TAC. ImageJ analysis showed that TAC induced EGR1, Gadd45b and Runx1 expression in mouse atriums. Expression of circNAB1 repressed Gadd45b and Runx1 but did not affect EGR1 expression in the atrium of TAC mice (**p < 0.01 versus wt; *n = 6*). g) IHC staining images showed expression of EGR, Gadd45b, Runx1, and NAB1‐356 in the atriums of normal and TOF or HF patients with or without AF. ImageJ analysis showed AF atriums expressed increased Gadd45b and Runx1, decreased NAB1‐356 compared to no‐AF cases in both TOF and HF patients (**p < 0.01 versus wt; *n = 6*). h) Western blot showed that AF atrium tissues expressed increased Gadd45b and Runx1 compared to no‐AF cases in HF patients. i) AC16 cells transfected with control vector, circNAB1 and NAB1 plasmid, were subjected to immunoprecipitation with antibodies against EGR1 or NAB1 (N‐T). Western blot showed that precipitation of EGR1 pulled down high levels of NAB1‐356 and low levels of NAB1. Whereas precipitation of NAB1‐356 pulled down high levels of EGR1, and precipitation of NAB1 pulled down low levels of EGR1. j) Chromatin from wt and circNAB1(+) mouse atrial tissues was isolated, digested, and immunoprecipitated with rabbit IgG and antibodies against histone, NAB1‐356, NAB1(N‐T) and NAB1(C‐T), followed by PCR with primers flanking a DNA sequence at the Gadd45b or Runx1 promoter. k) Real‐time PCR showed precipitating NAB1‐356 pulled down Gadd45b and Runx1 promoter sequences (**p < 0.01 versus wt; *n = 6*).

To uncover the molecular mechanism behind the roles of circNAB1, we performed RNA sequencing (RNA‐seq) on atrial tissues from circNAB1 transgenic and littermate‐negative mice, 4 weeks after TAC. Among the 342 differentially expressed genes identified (Table S2, Supporting Information), the heatmap displayed that the most significantly altered mRNAs at a 2‐fold cutoff were collagens that were downregulated by circNAB1 expression (**Figure** **4**a).

To examine the role of circNAB1 in atrial fibrosis, we harvested circNAB1(+) and wt mouse atriums followed by RT‐PCR analysis. After confirming elevated circNAB1 expression in circNAB1(+) mouse atriums (Figure S7a, Supporting Information), we detected reduced expression of fibrosis markers collagen‐I, collagen‐III, fibronectin, and vimentin (Figure 4b). PCFs isolated from circNAB1 transgenic mice also showed decreased expression, while silencing circNAB1 in HCFs displayed increased expression of these fibrosis markers (Figure S7b, Supporting Information).

Moreover, the mRNA sequencing results showed involvement of many cytokines. We tested the expression of several essential cytokines in the atrium and observed diminished expression of pro‐inflammatory cytokines IL‐1β, IL‐2, IL‐4, IL‐10, IL‐17, and TNF‐α, post‐TAC in the circNAB1(+) mice relative to wt mice (Figure 4c). PCMs isolated from circNAB1 transgenic mice also showed decreased expression, while silencing circNAB1 in AC16 displayed increased expression of several tested cytokines (Figure S7c, Supporting Information).

We then examined how circNAB1/NAB1‐356 would suppress fibrosis and cytokine expression. The full‐length protein NAB1 is known to function as a corepressor of early growth response (EGR) transcription factors.^[^ ^8^, ^9^ ^]^ This corepressor plays a pivotal role in cardiomyocytes, where it regulates cardiomyocyte growth through its interaction with EGR.^[^ ^12^ ^]^ We thus examined expression of transcription factors associated with fibrosis and cytokine expression. Both mRNA sequencing data and real‐time PCR showed significant downregulation of FGFR1, FGFR2, TGFbR1, TGFb3, POSTN, TIMP1, MMP2, PDK4, Runx1, Gadd45b, PTK2b, and PTK7, but upregulation of NR4a1, FLT1, CAV2, and CAVIN1 (Figure 4d). Runx1 and Gadd45b appeared to be the mostly downregulated transcription factors. Analysis of mouse atrium tissues showed transgenic expression of circNAB1 decreased Runx1 and Gadd45b levels but had no effect on EGR1 expression (Figure S7d, Supporting Information). Suppression of Runx1 and Gadd45b by circNAB1/NAB1‐356 was also confirmed in circNAB1 primary cells (PCMs, PCFs, and HCFs Figure S7e–g, Supporting Information) and cell lines (MCF, HL‐1, and AC16, Figure S7e–g, Supporting Information). Silencing circNAB1 increased Runx1 and Gadd45b expression (Figure S8a,b, Supporting Information). Ectopic expression of circNAB1 increased cardiomyocyte survival and decreased apoptosis (Figure S8c, Supporting Information), while silencing circNAB1 produced opposite effects (Figure S8d, Supporting Information).

In the TAC‐induced cardiac stress, Gadd45b and Runx1 expression was highly upregulated, but it was prevented in the circNAB1‐transgenic mice confirmed by Western blotting (Figure 4e) and IHC staining (Figure 4f; Figure S9a, Supporting Information). IHC staining revealed elevated expression of Gadd45b and Runx1 in AF atriums compared to those without AF, across both TOF and HF patient groups (Figure S9b, Supporting Information). ImageJ analysis further confirmed this trend, demonstrating increased Gadd45b and Runx1 levels alongside decreased NAB1‐356 expression in AF cases relative to no‐AF counterparts (Figure 4g). Similarly, Western blot analysis of AF atrium tissues from HF patients corroborated these findings, revealing heightened Gadd45b and Runx1 expression levels compared to non‐AF cases (Figure 4h), highlighting their potential roles in the pathogenesis in AF status.

To verify the role of NAB1‐356 in mediating circNAB1 functions, we generated a number of constructs including precursor (expressing pre‐circNAB1), circ‐mut (expressing untranslated mutant circNAB1), NAB1 (expressing full‐length NAB1 protein), and NAB1‐356 (no circNAB1 but the protein produced, Figure S10, Supporting Information). We found that NAB1‐356 protein, as well as circNAB1 could repress expression of Gadd45b and Runx1 (Figure S11a, Supporting Information), collagen‐1 and TNF‐a (Figure S11b, Supporting Information), leading to enhanced cell survival and decreased apoptosis (Figure S11c, Supporting Information).

Our results showed that neither NAB1 nor NAB1‐356 affected EGR1 expression. We then examined their binding interaction. Co‐immunoprecipitation showed that both NAB1 and NAB1‐356 interacted with EGR1: in fact, NAB1‐356 displayed stronger binding capacity to EGR1 than NAB1 (Figure 4i), showing that addition of the unique motif affected its binding activity to EGR1. In a competition assay, we found that EGR1 had much higher binding activity to interact with NAB1‐356 compared to the full‐length NAB1 (Figure S12a, Supporting Information).

We then performed chromatin immunoprecipitation assays: it revealed NAB1‐356 binding to the promoters of Gadd45b and Runx1, corroborated by gel electrophoresis images (Figure 4j; Figure S12b, Supporting Information) and real‐time PCR analyses (Figure 4k), indicating direct modulation of Gadd45b and Runx1 transcription.

### 2.5 Delivery of EGR1, Gadd45b and Runx1

**Figure 5:**
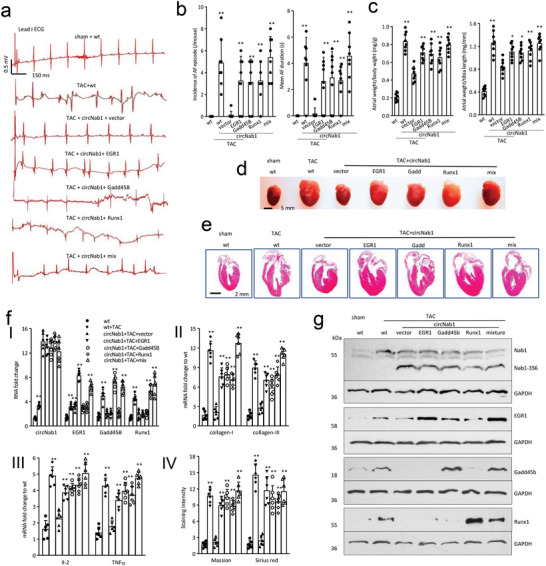
Delivery of EGR1, Gadd45b and Runx1 induced mouse AF, atrial fibrosis, and inflammation. a) Eight‐week‐old wt and circNAB1(+) mice were subjected to TAC and injected with EGR1, Gadd45b, Runx1 or a mixture twice a week for 12 weeks. Representative images recorded I lead surface ECG after termination of the in vivo transesophageal programmed burst of atrial stimulation. b) ECG analysis showed that circNAB1(+) mice displayed frequent AF episode and long mean AF duration after TAC and delivery with EGR1, Gadd45b or Runx1 during 3 series of bursts as well as wt mice (**p < 0.01 versus vector; *n = 8*). c) Quantification of the ratio of atrial weight/body weight or tibia of above mice (**p < 0.01 versus vector; *n = 8*). d) Typical images of the heart showed atrial sizes of circNAB1(+) mice after TAC and delivery with EGR1, Gadd45b or Runx1. e) Typical images of HE‐stained heart section of the above mice. f) I. PCR showed delivery of EGR1, Gadd45b or Runx1 increased EGR1, Gadd45b, and Runx1 mRNA in mouse atriums. II. PCR showed delivery of EGR1, Gadd45b or Runx1 increased collagen‐I and collagen‐III in mouse atriums. III. PCR showed delivery of EGR1, Gadd45b or Runx1 increased Il‐2 and TNF‐α mRNA in mouse atriums. IV. ImageJ analysis showed delivery of EGR1, Gadd45b or Runx1 increased Masson's trichrome and Sirius red staining in mouse atriums after TAC (**p < 0.01 versus vector; *n = 6*). g) Western blot showed delivery of EGR1, Gadd45b or Runx1 increased EGR1, Gadd45b, and Runx1 expression in mouse atriums.

To test whether the effects of NAB1‐356 on atrial fibrosis, cytokine synthesis, and AF were mediated through EGR1, Gadd45b, and Runx1, we induced fibrosis and AF in circNAB1‐transgenic mice through TAC surgery, followed by delivery of EGR1, Gadd45b, and Runx1 expression plasmids with nanoparticles. Eight‐week‐old wild‐type (wt) and circNAB1(+) mice were subjected to TAC and injected with EGR1, Gadd45b, Runx1, or a mixture of these factors twice a week for 12 weeks. Subsequent assessment via surface ECG recordings revealed representative images of lead I signals after termination of the in vivo transesophageal programmed burst atrial stimulation (**Figure** **5**a). ECG analysis revealed a high incidence of atrial fibrillation (AF), frequent AF episodes, and prolonged mean AF duration in circNAB1(+) mice following TAC and delivery of EGR1, Gadd45b, or Runx1, comparable to control vector delivered mice (Figure 5b; Figure S13a,b, Supporting Information). Furthermore, hearts from the injected mice were processed for programmed electrical stimulation of the right atrium under Langendorff‐perfusion conditions, capturing typical images of atrial and ventricular ECG recordings post‐stimulation (Figure S13c, Supporting Information). Typical images of hearts showed enlarged atrial sizes, as evidenced by both HE‐stained heart sections and quantification of the ratio of atrial weight to body weight or tibia length in the treated mice (Figure 5c–e). Additionally, representative images of Masson trichrome and Sirius red staining highlighted increased fibrotic deposition in the atriums of mice subjected to TAC and injected with EGR1, Gadd45b, or Runx1, underscoring the pro‐fibrotic effects of these transcription factors (Figure S13d, Supporting Information). These results collectively underscored the pro‐arrhythmic potential of EGR1, Gadd45b, and Runx1 in promoting AF in a mouse model, providing valuable insights into the mechanistic underpinnings of cardiac rhythm disturbances.

We further examined the exacerbation of atrial fibrosis and inflammation following the delivery of transcription factors EGR1, Gadd45b, and Runx1 in the cardiac stress induced by TAC. RT‐PCR analysis revealed increased expression of collagen‐I and collagen‐III, cytokines IL‐2 and TNF‐α. ImageJ analysis showed increased Masson's trichrome and Sirius red staining in mouse atriums injected with EGR1, Gadd45b, or Runx1 compared to vector controls, indicative of enhanced fibrotic deposition (Figure 5f). Western blotting confirmed the increased expression of EGR1, Gadd45b, and Runx1 at the protein level in treated mouse atriums (Figure 5g). Our results collectively highlight the pro‐fibrotic and pro‐inflammatory effects of EGR1, Gadd45b, and Runx1 delivery in promoting atrial remodeling under conditions of cardiac stress.

### 2.6 Silencing EGR1, Gadd45b and Runx1

**Figure 6:**
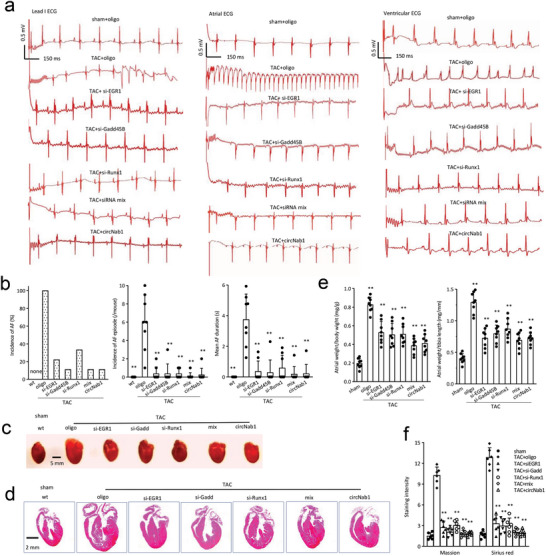
Silencing EGR1, Gadd45b and Runx1. a) Left, Eight‐week‐old wt mice were subjected to TAC and injected with siRNAs against EGR1, Gadd45b, Runx1 or a mixture of above siRNAs twice a week for 12 weeks. Representative images recorded I lead surface ECG (left) after termination of the in vivo transesophageal programmed burst of atrial stimulation. The hearts were processed to programmed electrical stimulation of the right atrium under Langendorff‐perfusion. Typical images recorded atrial (middle) and ventricular (right) ECG after termination of the ex vivo transesophageal programmed burst of atrial stimulation. b) ECG analysis showed that TAC mice displayed a high incidence of AF, frequent AF episode and long mean AF during 3 series of bursts, which could be prevented by delivery of siRNAs against EGR1, Gadd45b or Runx1 as well as transgenically expressing circNAB1 (**p < 0.01 versus oligo; *n = 8*). c) Typical images of the heart showed atrial sizes of mice delivery with siRNAs against EGR1, Gadd45b or Runx1 after TAC. d) Typical images of HE‐stained heart sections of the above mice. e) Quantification of the ratio of atrial weight/body weight or tibia of the above mice (**p < 0.01 versus oligo; *n = 8*). f) ImageJ analysis showed delivery of siRNAs against EGR1, Gadd45b or Runx1 decreased Masson's trichrome and Sirius red staining in mouse atriums (**p < 0.01 versus oligo; *n = 6*).

On the other hand, we silenced these transcription factors EGR1, Gadd45b, and Runx1 with siRNAs in the TAC mice. Hearts from treated mice were subjected to programmed electrical stimulation of the right atrium under Langendorff‐perfusion, capturing typical images of atrial and ventricular ECG recordings post‐stimulation. Mice subjected to TAC exhibited a high incidence of AF, frequent AF episodes, and prolonged mean AF duration, which could be effectively prevented by the delivery of siRNAs against EGR1, Gadd45b, or Runx1, as well as by transgenic expression of circNAB1 (**Figure** **6**a). ECG analysis showed that TAC mice displayed high incidence of AF, frequent AF episode and long mean AF during 3 series of bursts, which could be prevented by delivery of siRNAs against EGR1, Gadd45b or Runx1 as well as transgenically expressing circNAB1 (Figure 6b). We detected the attenuating effects of silencing transcription factors EGR1, Gadd45b, or Runx1 with small interfering RNAs (siRNAs) on atrial sizes (Figure 6c), also demonstrated by HE staining (Figure 6d) following TAC, indicative of diminished cardiac remodeling. Quantification of the ratio of atrial weight to body weight or tibia length corroborated these findings, highlighting the beneficial effects of siRNA‐mediated knockdown of EGR1, Gadd45b, or Runx1 on preventing atrial fibrosis (Figure 6e). RT‐PCR, immunoblotting and histological examination of heart sections further revealed decreased fibrotic deposition, as evidenced by reduced Masson's trichrome and Sirius red staining in the atriums of siRNA‐treated mice (Figure S14, Supporting Information). ImageJ analysis provided quantitative confirmation, showing a significant decrease in Masson's trichrome and Sirius red staining intensity in mouse atriums following siRNA delivery (Figure 6f). These results underscore the potential therapeutic value of targeting EGR1, Gadd45b, or Runx1 to mitigate atrial fibrosis and inflammation, offering promising strategies for the treatment of cardiac remodeling‐associated pathologies, offering promising avenues for AF management.

### 2.7 Delivery of circNAB1 Reduced the Incidence of AF in LKB1 KO Mice

**Figure 7:**
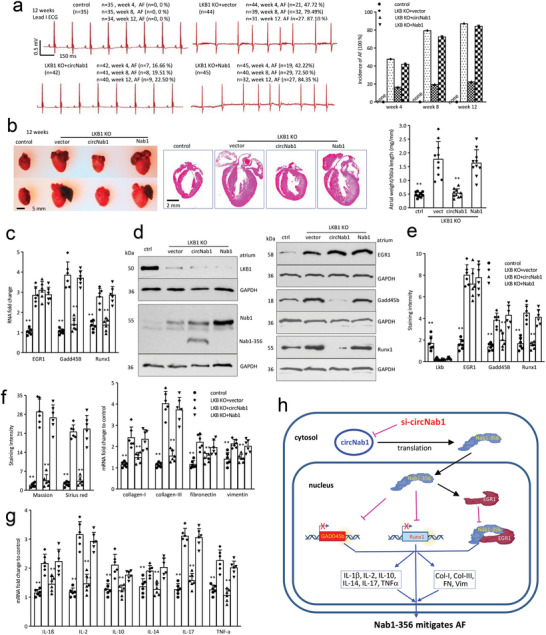
Delivery of circNAB1 reduced the incidence of AF in LKB1 KO mice by suppressing atrial fibrosis and inflammation. a) Left, Newborn (P0) mice were delivered with AAV9‐circNAB1 or ‐NAB1 (1 × 10^11^ vg per mouse) via retro‐orbital injection, after confirmed LKB1 KO, and repeated every 6 weeks. Surface ECG was recorded every 4 weeks. Representative images recorded I lead surface ECG at week 12. Right, ECG analysis showed that LKB1 KO mice developed AF spontaneously with high incidence. Delivery of circNAB1 significantly decreased AF incidence of LKB1 KO mice. n was indicated in the figures. b) Left, Typical images of the heart showed atrial sizes of LKB1 KO mice delivered with circNAB1 at week 12. Middle, Typical images of HE‐stained heart sections of above mice. Right, Quantification of the ratio of atrial weight/body weight or tibia of above mice (**p < 0.01 versus vector; *n = 10*). c) PCR showed that EGR1, Gadd45b and Runx1 expression increased in LKB1 KO mouse atriums. Expression of circNAB1 repressed Gadd45b and Runx1, but not affect EGR1 expression in mouse atriums (**p < 0.01 versus vector; *n = 6*). d) Western blot showed that EGR1, Gadd45b and Runx1 expression increased in LKB1 KO mouse atriums. Expression of NAB1‐356 repressed Gadd45b and Runx1, but not affect EGR1 expression in mouse atriums. e) ImageJ analysis showed that EGR1, Gadd45b and Runx1 expression increased in LKB1 KO mouse atriums. Expression of circNAB1 repressed Gadd45b and Runx1, but not affect EGR1 expression in mouse atriums (**p < 0.01 versus vector; *n = 6*). f) Left, ImageJ analysis showed delivery of circNAB1 decreased Masson's trichrome and Sirius red staining in LKB1 KO mouse atriums. Right, PCR showed that LKB1 KO mouse atriums expressed increased fibrosis markers, including collagen‐I, collagen‐III, fibronectin, and vimentin, which could be prevented by delivery of circNAB1 (**p < 0.01 versus vector; *n = 6*). g) PCR showed that LKB1 KO mouse atriums expressed increased cytokine IL‐1β, Il‐2, Il‐4, Il‐10, Il‐17, and TNF‐α, which could be prevented by delivery of circNAB1 (**p < 0.01 versus vector; *n = 6*). h) Signal pathway by which circNAB1/NAB1‐356 improves AF outcomes.

We then investigated the therapeutic potential of circNAB1 delivery in reducing the incidence of AF in LKB1 knockout (LKB1 KO) mice, a model of spontaneous AF development. Newborn LKB1 knockout mice received retro‐orbital injections of adeno‐associated virus 9 (AAV9) carrying circNAB1 or NAB1 every 6 weeks. After confirming expression of circNAB1 in mouse atriums (Figure S15a, Supporting Information), surface electrocardiogram (ECG) recordings at week 12 revealed that LKB1 KO mice developed AF spontaneously, with a high incidence, whereas delivery of circNAB1 significantly decreased AF incidence in these mice (**Figure** **7**a). Decreased atrial sizes and atrial weight/body weight ratio were observed in LKB1 KO mice delivered with circNAB1, highlighting the anti‐arrhythmic of circNAB1 in a mouse model of AF (Figure 7b). RT‐PCR analysis showed that EGR1, Gadd45b and Runx1 expression increased in LKB1 KO mouse atriums (Figure 7c). Typical Western blot (Figure 7d) and immunohistochemistry staining (Figure 7e; Figure S15b, Supporting Information) demonstrated increased expression of transcription factors EGR1, Gadd45b, and Runx1 in LKB1 KO mouse atriums. However, delivery of circNAB1 effectively suppresses the upregulation of Gadd45b and Runx1 without affecting EGR1 expression, confirmed by RT‐PCR, Western blot, and immunohistochemistry staining.

We examined the effect of circNAB1 on atrium fibrosis. Representative images of Masson trichrome and Sirius red staining reveal reduced fibrotic deposition in the atriums of mice delivered with circNAB1 compared to vector controls (Figure S15c, Supporting Information). ImageJ analysis further quantifies this reduction in fibrosis, confirming the efficacy of circNAB1 in attenuating atrial fibrosis and decreasing expression of fibrosis markers collagen‐I, collagen‐III, fibronectin, vimentin (Figure 7f). Consistently, circNAB1 delivery mitigates the increased pro‐inflammatory cytokines IL‐1β, Il‐2, Il‐4, Il‐10, Il‐17, and TNF‐α in LKB1 KO mouse atriums (Figure 7g). These results underscore the therapeutic potential of circNAB1 in suppressing atrial fibrosis and inflammation associated with AF, offering promising avenues for AF management in LKB1‐deficient individuals.

## 3 Discussion

Atrial fibrillation (AF) represents a significant global health burden due to its association with increased morbidity and mortality. Despite significant strides in understanding its pathophysiology and the underlying mechanisms, effective therapeutic strategies remain limited. In this study, we investigated the expression and functional significance of circNAB1, a circular RNA, in AF pathogenesis. We showed that the circular RNA circNAB1 and its encoded protein NAB1‐356 are beneficial in improving the conditions and outcomes of AF. Our findings offer insights into the complex interplay between NAB1‐356/circNAB1, fibrosis, inflammation, and transcriptional regulation. This comprehensive understanding presents promising avenues for therapeutic exploration aimed at mitigating the burden of AF.

The dysregulation of non‐coding RNAs, including circular RNAs, has emerged as a pivotal factor in various cardiovascular diseases, including AF.^[^ ^28^, ^29^, ^30^, ^31^ ^]^ Through high‐throughput circular RNA sequencing, we detected circNAB1 as one of the significantly downregulated circRNAs in AF patient specimens compared to healthy donor controls. circNAB1 and its encoded protein NAB1‐356 were detected in both cardiomyocytes and cardiac fibroblasts of human atriums, with high levels in hypertrophy hearts. Interestingly, NAB1‐356/circNAB1 levels significantly decreased in the heart specimen with AF. To corroborate this observation, we selected 15 circRNAs, based on their expression abundance and fold‐changes, for further investigation. Silencing these circRNAs not only confirmed the circRNA‐seq results but also suggested a potential role for circNAB1 in AF pathogenesis, prompting further investigation into its functional significance.

Our functional assays revealed a regulatory role for circNAB1 in cardiac fibrosis and cytokine production, the hallmarks and pre‐requirement of AF. Notably, silencing endogenous circNAB1 led to increased collagen deposition, whereas ectopic circNAB1 overexpression in atrial cardiomyocytes with a myosin heavy chain promoter attenuated atrial fibrosis in murine cardiac remodeling model, consistent with results observed in human atrial tissues that decreased circNAB1 was detected in human AF specimens. Increased function of circNAB1 was associated with decreased production of cytokines such as TNF‐α, IL‐1β, IL‐2, IL‐4, IL‐10, and IL‐17, cytokines that modulated tissue fibrosis.^[^ ^32^, ^33^ ^]^ Our findings suggest the anti‐fibrotic activities of circNAB1 may confer it as a potential therapeutic target for AF‐associated cardiac remodeling through regulating cytokine syntheses. Furthermore, our mechanistic elucidation revealed that the effects of circNAB1 on fibrosis were, at least partially, mediated through its interaction with transcription factors EGR1, Runx1 and Gadd45b, which are known key regulators of fibrotic processes and disease development.^[^ ^34^, ^35^, ^36^, ^37^ ^]^ Our current study suggested that significantly elevated expression of circNAB1/NAB1‐356 in cardiac hypertrophy suppressed Runx1 and Gadd45b expression, through directly binding to their promoters and inhibiting fibrotic related transcription. As a master regulator and transcriptional sensor in vascular dysfunction, EGR1 is functionally activated by hypertrophy, myocardial ischemia/reperfusion, and other related cardiac stress, modulated by its corepressor NAB1. circNAB1/NAB1‐356 showed much stronger binding capacity to EGR1 than NAB1, attenuating AF pathways via blocking abnormally activated EGR1 mediated cardiac transcription (Figure 7h).

The therapeutic targeting of the key transcription factors implicated in AF pathogenesis represents a particularly promising avenue. In our study, we demonstrated that circNAB1‐mediated suppression of EGR1, Gadd45b, and Runx1 effectively attenuated AF incidence and cardiac remodeling in the murine models. Conversely, delivery of EGR1, Gadd45b, and Runx1 exacerbated atrial fibrosis and inflammation, further highlighting their pathogenic roles in AF progression. These findings provide mechanistic insights into the transcriptional regulation of cytokine production and cardiac remodeling. Our study suggests the therapeutic potential of targeting these transcription factors in AF management.

An intriguing discovery derived from our study was the identification of a novel protein, NAB1‐356, translated from circNAB1. The translation of circRNAs into proteins has attracted increasing attention in recent years.^[^ ^22^ ^]^ These resulting proteins have been shown to exert strong biological functions and have been regarded as new protein isoforms specifically encoded by circRNAs.^[^ ^38^, ^39^, ^40^ ^]^ NAB1‐356 exhibited regulatory effects on fibrosis and cytokine expression, which are the key factors in AF development.^[^ ^41^ ^]^ Our study adds to the growing body of evidence supporting the functional significance of circRNA translation, demonstrating the key role of the resulting protein in cardiovascular health and disease.

Moreover, our study uncovered the therapeutic potential of circNAB1 in LKB1‐deficient mice, a model of spontaneous AF development.^[^ ^42^ ^]^ Delivery of circNAB1 effectively suppressed atrial fibrosis and inflammation in LKB1 KO mouse atriums. This further highlighted the anti‐arrhythmic properties of circNAB1/NAB1‐356 in a clinically relevant setting. These findings not only expand our understanding of circNAB1 function but also present promising therapeutic avenues for AF treatment, particularly in patients with underlying genetic predispositions.

In conclusion, our study elucidates the intricate roles of circNAB1/NAB1‐356 in AF pathogenesis and cardiac remodeling. By modulating collagen deposition, tissue fibrosis, inflammation, and transcriptional regulation, circNAB1/NAB1‐356 emerges as a potential therapeutic target for AF management. Future studies exploring the clinical translation of circNAB1‐based therapies and further uncovering the molecular mechanisms underlying circNAB1/NAB1‐356 actions are warranted. Ultimately, the insights gained from our study may pave the way for the development of novel therapeutic strategies aimed at mitigating the burden of AF and improving patient outcomes.

## 4 Experimental Section

### Constructs, Primers, and siRNAs

Plasmids encoding circNAB1, NAB‐356 (a translation fragment expressed by the pcDNA3.1 plasmid that does not form circular RNA), circNAB1 precursor (a plasmid that does not form circular RNA), circNAB1‐mut or circ‐mut (circNAB1 containing a point mutation to disrupt protein translation), were generated by Gene Universal. The vector plasmid contains a Bluescript backbone, with one CMV promoter driving green fluorescent protein (GFP) expression, and another CMV promoter driving the circular RNA‐forming fragments or a non‐related sequence serving as control. The plasmid containing the full‐length human NAB1 gene was obtained from DNASU, and plasmids encoding EGR1, Runx1 and Gadd45B were from Addgene. Recombinant adeno‐associated virus (AAV) 9 expressing above circNAB1 or NAB1 was generated and packed by WZ Biosciences. Construct, primer and siRNA sequences and all materials used are listed in the Major Resources Table.

### Human Heart Sample Collection

The study was conducted in accordance with The Ethics Code of the World Medical Association. Heart samples were collected from individuals with overloading heart diseases (n = 106), including Tetralogy of Fallot (TOF, n = 91), hypertrophic cardiomyopathy (HCM, n = 7), aortic stenosis (AS, n = 5), and mitral stenosis (MS, n = 3), all with normal left ventricular ejection fraction (LVEF) above 40%. Heart failure cases (HF, n = 33) were selected from individuals with pressure overloading (PO) heart diseases exhibiting reduced ejection fraction (HFrEF), with LVEF below 40%. The hearts without detectable cardiovascular disease were collected from 23 individuals who died from non‐cardiac related causes. All patients provided formal informed consent prior to enrollment in the study. Heart samples were obtained from heart donations or surgeries, collected 0.5–4 h after the patient's death or surgery. The sample information was provided in Supplementary Table S3 (Supporting Information).

All cases were classified into subgroups: normal rhythm, arrhythmia (AR), and AF. The arrythmia group included individuals with supraventricular tachycardia (SVT), premature atrial contractions (PAC), premature ventricular contractions (PVC), atrioventricular node block (AV node block) and ventricular conduction block. Atrial fibrillation (AF) is an irregular heart rhythm that originates in the atria. A typical AF ECG shows no visible P waves and an irregularly irregular QRS complex. The AF cases in this study included only individuals with persistent AF lasting for more than a week. Supraventricular tachycardia (SVT) is a dysrhythmia that originates at or above the atrioventricular (AV) node and is defined by a narrow QRS complex (<0.12 s) at a heart rate between 150–220 beats per minute (bpm). The SVT cases contained different types of SVT (including paroxysmal SVT, PSVT), with ECG or ambulatory monitoring showing heart rate between 150–220 bpm and QRS<0.12 s, and experienced typical SVT symptoms including, palpitations, dizziness, fatigue, shortness of breath or fainting, but excluded all those no symptom PSVT cases. Premature ventricular contractions (PVCs) are extra heartbeats that begin in ventricles, with wide abnormally shaped QRS complexes that occur earlier than expected. Premature atrial contractions (PACs) are premature heartbeats that occur in atria, an early P wave that has a different shape than a normal P wave. All the occasional PAC/PVC and less than 5 PACs/PVCs per minute on ECG or 30 PACs/PVCs per hour in during ambulatory monitoring cases were excluded.

Tissue from the left atrium was removed and parceled into four portions. Two fragments were collected into cryovials, stored in liquid nitrogen, used for RNA or protein isolation. The remaining two fragments were used for paraffin or frozen sectioning.

### Circular RNA Sequencing

High‐throughput circular RNA sequencing was performed in the auricles of patients clinically diagnosed with atrial fibrillation (AF) and non‐AF (normal) cases. The non‐AF samples were from donor hearts intended for transplantation. Four AF cases were diagnosed with persistent AF lasting for more than one week. These patients exhibited typical AF electrocardiograms (ECGs), characterized by absent P waves and an irregularly irregular QRS complex before surgery. Four non‐AF samples were collected from patients with no AF history and showed normal ECG before surgery.

The heart tissues were subject to RNA isolation and library construction. In brief, a total of 5 µg RNA per sample was used. The total RNA samples were then subjected to RNase R treatment to remove linear RNAs. They were also depleted of rRNA by using Epicentre Ribo‐zero rRNA Removal Kit (Epicentre, USA). The rRNA‐depleted RNAs were then treated with RNase R (Epicentre, USA) and subjected to Trizol extraction. RNase R‐resistant circular RNAs were subjected to electrophoresis on 1% agarose gels to monitor potential contamination and degradation. The purity of RNA samples was confirmed by NanoPhotometer spectrophotometer (IMPLEN, CA, USA).

The RNAs were then fragmented using divalent cations in NEBNext UltraTM RNA Library Prep Kit for Illumina (NEB, USA). Adenylation of 3′ ends, adaptor ligation, reverse transcription, PCR amplification, and purification of the cDNAs were performed following the manufacturer's instruction. The purified cDNAs were subjected to the generation of library. The cDNA library was sequenced and analyzed by Novogene (www.novogene.com) using an Illumina HiSeq 2500 System. Each sample was independently sequenced.

Based on the sequencing results, all 125‐bp to 150‐bp paired‐end reads were obtained. Reference genome and gene annotation were established using Bowtie v2.0.6. Paired‐end clean reads of each sequence were aligned to the reference genome by using TopHat v2.0.9. Unmapped reads were kept. All 20‐mers from 5′ and 3′ end of these reads were extracted and aligned independently to identify circular RNAs. Every circular RNA was recorded by at least two clean reads spanning a head‐to‐tail back splice junction in each sample. All potential circular RNAs were compared with the reported circular RNAs^[^ ^27^ ^]^ to determine known and unknown circular RNAs.

A total of 46667 circular RNAs were identified in these samples, by at least two clean reads spanning a head‐to‐tail back splice junction in the samples. Compared to normal samples, the numbers of differentially expressed circular RNAs at a twofold cut‐off were calculated for each AF sample. The significant difference was then determined between the normal and AF tissues. The analysis was performed by Novogene. They used two algorithms to avoid false positives.^[^ ^43^, ^44^ ^]^

### Animal Preparation

The circNAB1‐transgenic mouse model was established through pronuclear microinjection of a myosin heavy chain promoter and circNAB1‐containing DNA fragment into C57BL/6J mice, performed by the Toronto Centre for Phenogenomics (TCP). Cardiac‐specific LKB1 knockout (LKB1 KO) mice were generated by crossing LKB1*^flox/flox^* with transgenic mice expressing Cre‐recombinase from the α‐MHC promoter as described previously.^[^ ^45^ ^]^ The transgenic mice were confirmed by genotyping, PCR, and Western blot analysis before the experiments. Wild‐type (wt) mice were of the same strain (C57BL/6J) as the corresponding transgenic control mice.

All experimental procedures were approved by the Animal Care Committee of Sunnybrook Research Institute and performed in accordance with related guidelines and regulation. All treatment allocation and outcome assessment were blinded to the direct experimenters. Mice were anesthetized using 0.5% to 3.0% isoflurane during experimental procedures, including surgery, atrial stimulation, electrocardiogram (ECG) recording and termination. Pressure overload (PO) was induced by modified transverse aortic constriction (TAC) in mice as previously described.^[^ ^46^ ^]^ Successful establishment of the PO model was confirmed by measuring the carotid artery flow velocities using Doppler ultrasound. Only mice with a right carotid (RC)/left carotid (LC) flow ratio above 5 were used for further experiments. The sham mice underwent surgery without aortic banding. Twelve weeks after surgery, all mice underwent in vivo transesophageal programmed electrical stimulation of left atrium, with surface ECG (lead I) analysis. Atrial and ventricular ECG were also analyzed following programmed electrical stimulation of right atrium of above mouse hearts under Langendorff‐perfusion or in open chest stimulation model. Subsequently, the hearts were harvested and cut into two halves. The upper half of the heart was kept frozen for PCR or protein analysis. The lower half was fixed with 10% buffered formalin and embedded in paraffin or subjected to frozen sections.

### Delivery of siRNAs or Plasmids with Nanoparticles

SiRNAs or plasmids were conjugated with polyethylene glycol (PEG) and gold nanoparticles (Au NPs) to form complexes prior to injection. The synthesis of these complexes (siRNA‐ or plasmid‐PEG‐Au NP) was carried out as previously described.^[^ ^47^ ^]^ In brief, siRNAs or plasmids (20 nmol) were dissolved in 800 µL of RNase‐free water and mixed with mPEG‐SH at a 20:1 molar ratio. The resulting siRNA‐ or plasmid‐PEG conjugates were then mixed with 10 nm Au NP at weight ratio of 1:1 for conjugation. The mixture was gently shaken at 60 °C for 30 min and transferred into a syringe. The siRNA‐ or plasmid‐PEG‐Au NP conjugate was administered intraperitoneally in a volume of 100 µl twice a week as previously after TAC surgery.

### Delivery of Recombinant Adeno‐Associated Virus

Recombinant adeno‐associated virus serotype 9 (AAV9) expressing a control vector, circNAB1 and NAB1 were packed by WZ Biosciences. Newborn (P0) mice received AAV9‐circNAN1 or ‐NAB1 (1 × 10^11^ vg per mouse, 50 µl saline) via retro‐orbital injection, following confirmed LKB1 KO, and this administration was repeated every 6 weeks (5 × 10^11^ vg per mouse, 100 µl saline).

### Induction of AF and ECG Analysis

To assess the incidence of spontaneous AF in the mouse model, tested mice were anaesthetized with 2% isoflurane, and surface ECG was recorded using electrodes configured in a lead‐I configuration. The positive electrode (red) was placed at the left forelimb, the negative electrode (black) at the right forelimb, and the reference electrode (green) at the left hind‐limb.

Due to the low occurrence rates of AF in TAC model, AF inducibility was tested by applying a 2‐s burst with programmed electrical stimulation, with a cycle ranging from 40 to 20 ms with 2 ms decrement. A series of bursts was repeated three times after a stabilization period of 5 min. AF was identified in surface and ventricular ECGs by the presence of a typical narrow‐complex “irregularly irregular” QRS pattern with no distinguishable P waves. In this study, AF was defined as a rapid, irregular atrial rhythm with irregular R‐R intervals lasting for at least one second. The duration of AF was defined as the interval between the onset of the irregular atrial rhythm triggered by the bursts and the onset of the first normal sinus beat. AF was considered inducible if one or more bursts triggered an AF episode; otherwise, it was considered as not inducible.

For induction of AF in vivo, mice were anesthetized with 2% isoflurane supported by a ventilator, while maintaining a body temperature of 37 °C with a heating pad. A 2.3 French octapolar catheter was placed at the esophageal position dorsal to the left atrium. Surface ECG was simultaneously recorded using electrodes in a lead‐I configuration.

For induction of AF in ex vivo, mouse hearts were isolated after intraperitoneal administration of heparin (1 U g^−1^) and urethane (1 mg g^−1^), and retrogradely perfused using a Langendorff perfusion apparatus. The hearts were perfused with Krebs‐Henseleit buffer with 95% O_2_ at 37 °C and 80 mmHg pressure. Bipolar stimulating electrodes were pressed against the right atrium surface for programmed electrical stimulation. Bipolar recording electrodes were placed on the left atrium surface to record atrial ECG, and another pair of recording electrodes were placed on the apex and anterior wall of the heart to record ventricular ECG. A common ground electrode was placed on the base of the heart.

Mice were also subjected to open chest atrium stimulation. They were anesthetized with 2% isoflurane supported by ventilation with intratracheal intubation. Mouse body temperature was monitored and kept at 37 °C with a heating pad. Bipolar stimulating electrodes were pressed against the right atrium surface for programmed electrical stimulation. Bipolar recording electrodes were placed on the left atrium surface to record atrial ECG. A pair of recording electrodes were placed on the apex and anterior wall of the heart to record ventricular ECG. Another electrode was placed on the chest wall as common ground electrode. Surface ECG was also simultaneously recorded using electrodes in a lead‐I configuration.

### Cardiac Fibrosis Staining

Masson's Trichrome and Sirius red staining was performed with kits as previously described.^[^ ^48^ ^]^ For Masson's Trichrome staining, tissue sections were de‐paraffinized with xylene and hydrated with ethanol and distilled water. Subsequently, the sections were incubated in Bouin's solution at 58 °C for 60 min, washed, and stained with Modified Weigert's Iron Hematoxylin for 5 min. Samples were then processed to acid‐alcohol solution (5 s), Biebrich Scarlet‐Acid Fuchsin solution (5 mins), 1% Phosphomolybdic acid solution (2 mins), Aniline Blue solution (5 mins), and acetic acid solution (30 s). After washed and dehydrated, the slides were mounted.

For Sirius red staining, tissue sections underwent de‐paraffinization with xylene, hydrated with ethanol and distilled water, followed by staining with Modified Weigert's Iron Hematoxylin for 5 min. After washing, the sections were stained with picro‐sirius red solution for 1 h. Following a wash with acetic acid water, stained slides were processed for dehydration and mounting.

### Isolation of Primary Mouse Cardiomyocytes (PCMs) and Cardiac Fibroblasts (PCFs)

The isolation of PCMs and PCFs from neonatal mice was performed as previously described.^[^ ^7^ ^]^ Briefly, mice were heparinized, anaesthetized and euthanized by cervical dislocation. Hearts were rapidly isolated, washed in PBS with 20 mM BDM, and minced into small pieces. The minced tissues were digested with 0.01 g Collagenase D, 0.01 g Collagenase B, and 0.001 g Protease XIV in 20 ml Tyrode solution at 37 °C for 30 min. Following digestion, mixtures were centrifuged at 800 rpm for 10 min. The resulting cell pellet was resuspended in DMEM/F12 medium with 10% FBS and 20 mm BDM, and plated onto a 10 cm cell culture dish and incubated for 3 h in a cell culture incubator. This step facilitated the isolation of PCFs, which adhered to the uncoated cell culture dish. Non‐adherent cardiac cells were then transferred to a gelatin‐coated cell culture dish to collect PCMs.

### Cell Culture of Human Cardiac Fibroblasts (HCFs)

The Human Cardiac Fibroblasts (HCF, C‐12375) were purchased from Millipore Sigma, and cultured in DMEM/F12 medium with 10% FBS in 37 °C, 5% CO_2_ humidified incubator. The culture medium was changed every other day until the cells reach 60% confluency. After reaching 80% confluency, cells could be sub‐cultured for experiments.

### Purification of Polyclonal Antibodies

Polyclonal antibodies were raised in rabbits against NAB1‐356 junction peptide (VKPIQSNGCGLTQDPGGVAAV) by Genscript. The antibodies were purified with histidine‐tagged NAB1‐356 bound Ni‐NTA resins in our lab as previously described.^[^ ^7^ ^]^ Escherichia coli BL21 (DE3) competent cells (ThemoFisher Scientific) were transformed with the NAB1‐356 expressing pET‐28α plasmid linked with an N‐terminal histidine tag. The amplified protein was incubated and bound with Ni‐NTA beads (QIAGEN). The Ni‐NTA resin beads were washed three times with 50 ml buffers (20 mM Tris‐HCl (pH 8.0), 0.5 M NaCl, 1 mM DTT, and 20 mM imidazole) containing 8, 4, or 2 m urea respectively, and then washed with 50 ml Tris‐buffer saline (10 mM Tris‐HCl, pH 7.4 and 150 mm NaCl). The washed beads were incubated with 10 ml polyclonal rabbit antibodies against NAB‐356 junction peptide at 4 °C for 24 h. The resin was then washed with 50 ml washing buffer (20 mm Tris‐HCl (pH 7.4), 1 M NaCl, and 0.5% Triton X‐100) and 50 ml TBS. The bound antibodies were eluted with 5 ml antibody elution buffer (20 mm Tris‐HCl (pH 7.4), 0.15 m NaCl, and 6 m guanidine‐HCl). The eluted products were purified using a Spectra/Por Float‐A‐Lyzer G2 Dialysis device (Spectrum Lab, 5 KD), and concentrated using a 10 kDa MWCO Millipore Amicon Centrifugal Filter.

### Chromatin Immunoprecipitation

Chromatin immunoprecipitation (ChIP) was performed using the SimpleChIP chromatin IP kit (Cell Signaling) according to the manufacturer's instructions. Atrial tissues or cells were treated with formaldehyde solution, and the chromatin was lysed, digested and immunoprecipitated with rabbit IgG and antibodies against histone, NAB1‐356, NAB1(N‐T) and NAB1(C‐T). A total of 15% of the inputs were used for immunoblotting. The captured chromatin was eluted, crosslinking was reversed, and the DNA was recovered. ChIP DNA was subjected to real‐time PCR using specific primers flanking the promoter region of Gadd45B or Runx1.

### Sucrose Gradient Assay

Sucrose gradient assay was used to evaluate the translation of circNAB1. Control vector‐ or circNAB1‐tranfected cells were cultured with or without 100 µg mL^−1^ puromycin for 10 min lysed in hypotonic buffer containing 5 mM Tris‐HCl (pH 7.4), 2.5 mm MgCl2, 1.5 mm KCl, 1x EDTA‐free protease inhibitor cocktail, 0.5% Triton X‐100, 0.1 mg mL^−1^ cycloheximide, and 0.5% sodium deoxycholate. Sucrose gradients with 10% to 50% sucrose solutions were prepared in tubes. The polysome lysate was loaded on top of the sucrose gradient solution followed by ultracentrifuge at 30000 rpm at 4 °C for 2 h. The polysome fractions were collected from top to bottom. The absorbance at 254 nm (A254 nm) was measured, and RNA extraction of each fraction was performed. The RNA levels of circNAB1 in each fraction were determined by real‐time PCR. GAPDH mRNA levels in each layer were used as internal controls.

### Cell Survival Assay

Cells were cultured in 10% FBS with basal medium in 12‐well culture dishes (4 × 10^4^ cells/well) and maintained at 37 °C for 12 h. The medium was then replaced with H_2_O_2_‐containing medium at the indicated concentrations, and cells were cultured for the specified time points. Subsequently, the harvested cells were stained with Trypan Blue and counted using a Coulter Counter under the inverted microscope.

### Annexin‐V Assay

Cells were cultured in basal medium containing H_2_O_2_ with indicated concentration and harvested at specific time points. The harvested cells were resuspended in Annexin V‐APC binding buffer, and incubated with Annexin V‐APC and propidium iodide solutions for 30 min at room temperature. The Annexin V‐APC stained cells were analyzed by flow cytometry.

### Western Blotting

Atrial tissues or cells were lysed and subjected to sodium dodecyl sulfate‐polyacrylamide gel electrophoresis (SDS‐PAGE), and the proteins were transferred to nitrocellulose membrane in Tris/glycine buffer containing 20% methanol at 60 V at 4 °C for 2 h. After blocked in a TBST washing buffer (10 mM Tris‐Cl, pH 8.0, 150 mM NaCl, 0.05% Tween‐20) and 5% milk for 0.5 h, the blot was incubated with 1:4000 primary antibodies at 4 °C overnight. The membranes were then washed with washing buffer three times for 30 min each and incubated with secondary antibodies for 2 h. After washing, the expression of proteins was visualized with an ECL detection kit.

### Real‐Time PCR

Atrial tissues or cells were lysed, and total RNA was extracted with the RNA mini kit (Geneaid). Real‐time PCR was performed with SYBR Green PCR Kit (Bio‐Rad) using 2 µl cDNA as a template with a pair of primers. The thermocycler runs 30 cycles, denaturation at 95 °C for 15 s, annealing 56 °C for 5 s and extension step of 72 °C for 5 s. The ΔΔCT method was used to quantify all relative RNA levels using small nuclear RNA U6 as the reference and internal control.

### Fluorescence in Situ Hybridization (FISH)

An Alexa 555 labeled DNA oligo probe against circNAB1 was generated by fluorescence PCR labeling kit from Biolynx. The labeled probe was heated at 95 °C for 2 min and chilled on ice before use. The fixed sections were dehydrated for 2 min in 50%, 90%, and 100% ethanol, and then pretreated with hybridization solution in 55 °C for 30 mins. Slides were incubated with 100 nM fluorescence labeled probe in hybridization buffer at 55 °C for 4 h. After washing with saline‐sodium citrate (SSC) buffers, the slides were subjected to the next step of immunofluorescence staining.

### Immunofluorescence Staining

Sections were de‐paraffinized with xylene, and hydrated with ethanol and Tris‐Buffered‐Saline (TBS) containing 0.005% Triton X‐100. After blocked with 10% goat serum, section samples were incubated with primary antibody in 10% goat serum overnight. The slides were washed and incubated with Alexa Fluor 488, 555 or 647 conjugated secondary antibodies at room temperature for 1.5 h, followed by staining with DAPI. Images of the stained samples were captured using Nikon N‐SIM S confocal laser scanning microscopy. ImageJ was used for analysis and quantification of the staining results.

### Immunohistochemistry Staining (IHC)

Tissue sections were de‐paraffinized with xylene and hydrated with ethanol, Tris‐Buffered‐Saline (TBS) and then boiled in a pressure cooker for with high pressure for 15 mins. After washing with TBS, the sections were blocked with 10% goat serum and incubated with 1:200 primary antibody with 10% goat serum overnight. The slides were then incubated with biotinylated secondary antibody for 1.5 h, followed by avidin conjugated horseradish peroxidase. The section samples were stained with DAB kit, and then counterstained with Mayer's Hematoxylin.

### Statistical Analysis

Data were presented as mean with standard deviation (mean ± SD). The Shapiro‐Wilk test was used to determine the normality of distribution if the tested group size (n) was ≥6. A two‐tailed unpaired Student's *t*‐test was performed to assess the difference between two groups with a single independent factor. For multiple group analyses, a one‐way ANOVA followed by a Bonferroni post hoc test for one independent variable, and a two‐way ANOVA followed by a Bonferroni correction for two independent variables were performed. Pearson correlation was used to analyze the linear relationship between two variables, and a simple linear regression analysis was used to estimate parameters in a linear equation that predicted values of one variable based on the other. Prism 8 software (GraphPad Software: La Jolla, CA) was used for the statistical analyses mentioned above with statistical significance set at *p < 0.05*.

### Ethics Approval

The use of human heart tissues was conducted with guidelines and regulations approved by the Ethics Committee of Guangdong Provincial People's Hospital & Guangdong Academy of Medical Sciences. All animal experiments were conducted in accordance with the relevant guidelines and regulations approved by the Animal Care Committee of Sunnybrook Research Institute. The approval number for animal experiments is AUP#2004‐244.

## Conflict of Interest

The authors declare no conflict of interest.

## Author Contributions

B.B.Y. supervised the project. W.W.D. designed the experiments. W.W.D., M.R., X.L., J.W. performed the experiments. S.W. performed computational analysis of circRNA interaction. W.W.D. and B.B.Y. analyzed the data. WWD and BBY wrote the paper.

## Supporting information



Supporting Information

## Data Availability

The data that support the findings of this study are available in the supplementary material of this article.
