# Supplementary material for: A Novel Protein NAB1‐356 Encoded by circRNA circNAB1 Mitigates Atrial Fibrillation by Reducing Inflammation and Fibrosis
Source: Adv Sci (Weinh). 2025 Mar 27;12(20):2411959. doi: 10.1002/advs.202411959 (PMC12120700; doi:10.1002/advs.202411959)
Supplement: Supplementary file 1 — Supporting Information [file ADVS-12-2411959-s001.pdf]

ADVANCED SCIENCE
Open Access

Supporting Information

for *Adv. Sci.*, DOI 10.1002/advs.202411959

A Novel Protein NAB1-356 Encoded by circRNA circNAB1 Mitigates Atrial Fibrillation by Reducing Inflammation and Fibrosis

*William W Du, Muhammad Rafiq, Hui Yuan, Xiangmin Li, Sheng Wang, Jun Wu, Jinfeng Wei, Ren-Ke Li, Huiming Guo and Burton B Yang**

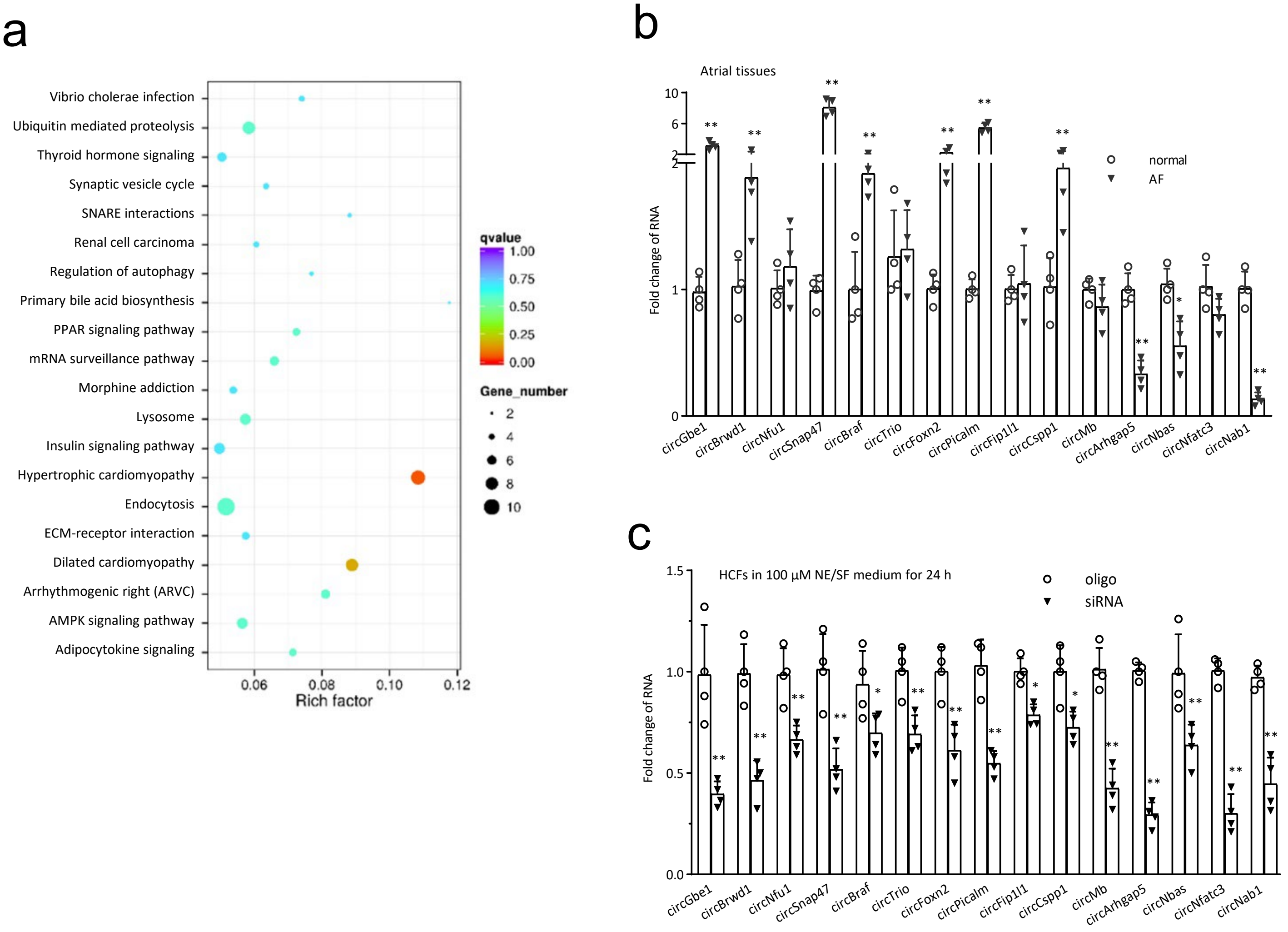

Online Figure S1. Characterization of circular RNAs associated with atrial fibrillation (AF).

(a) Enrichment of GO pathway. Circles represent the number of genes involved in the associated pathways, with sizes ranging from 1 to 14. The position of each circle indicates the enrichment of these genes, while numbers denote their ranking among all pathways. The color scale represents q values (adjusted p values, range: 0.00-1.00), indicating the significance of gene enrichment within each pathway, with the most significant enrichments highlighted in red.

(b) Fold change of 15 circular RNAs in atrial tissues analyzed by real-time PCR (*$p<0.05$, **$p<0.01$ versus normal; $n=6$).

(c) Fold change of 15 circular RNAs in HCFs transfected with their siRNAs analyzed by real-time PCR. Cells were transfected with circRNA siRNAs and cultured with serum-free DMEM/F12 medium with 100 μM NE for 24 h (*$p<0.05$, **$p<0.01$ versus oligo; $n=6$).

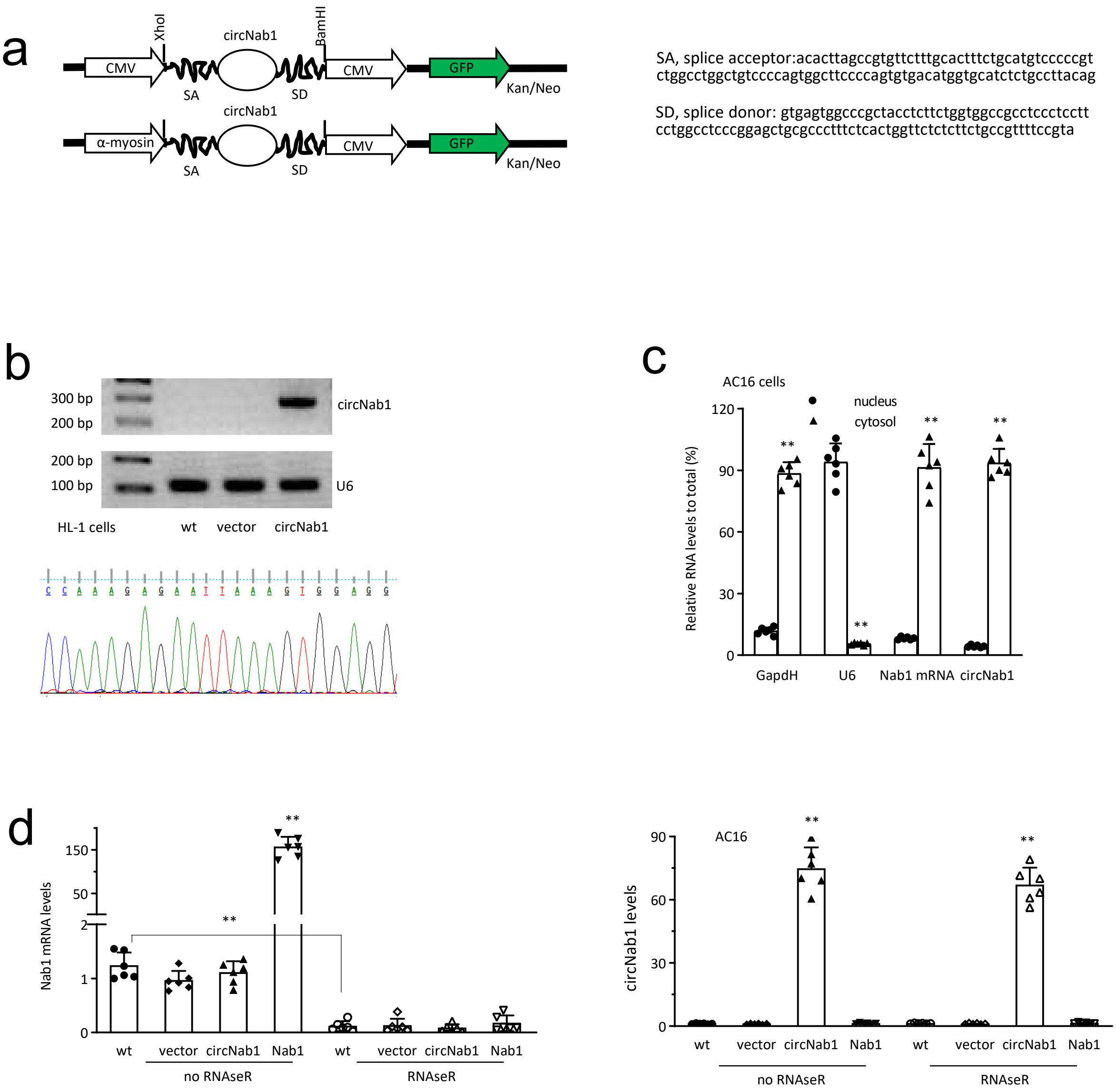

Fig S2. Development of circNAB1 transgenic mice

(a) Schematic representation of the constructs utilized in this study.

(b) The human circNAB1 construct was transfected into the mouse atrial cardiomyocyte cell line HL-1. Circular junctions were obtained by RT-PCR, cloned using TA-Cloning, and subsequently sequenced, confirming the expression junction sequence of human circNAB1.

(c) Real-time PCR analysis of RNAs extracted from the cytoplasm and nuclei of AC16 cells, with primers amplifying GAPDH, U6, NAB1, and circNAB1. High levels of NAB1 and circNAB1 were predominantly detected in the cytoplasm (***p<0.01* versus nucleus; *n=6*).

(d) Total RNA from vector-, circNAB1-or NAB1 transfected AC16 cells was subjected to incubation with or without RNAse R in 37 °C for 10 min, followed by RNA spike-in using mouse total RNA and subsequent real-time PCR. While RNAse R treatment decreased linear NAB1 levels (left), it did not affect circNAB1 levels (right) (***p<0.01* versus control vector; *n=6*).

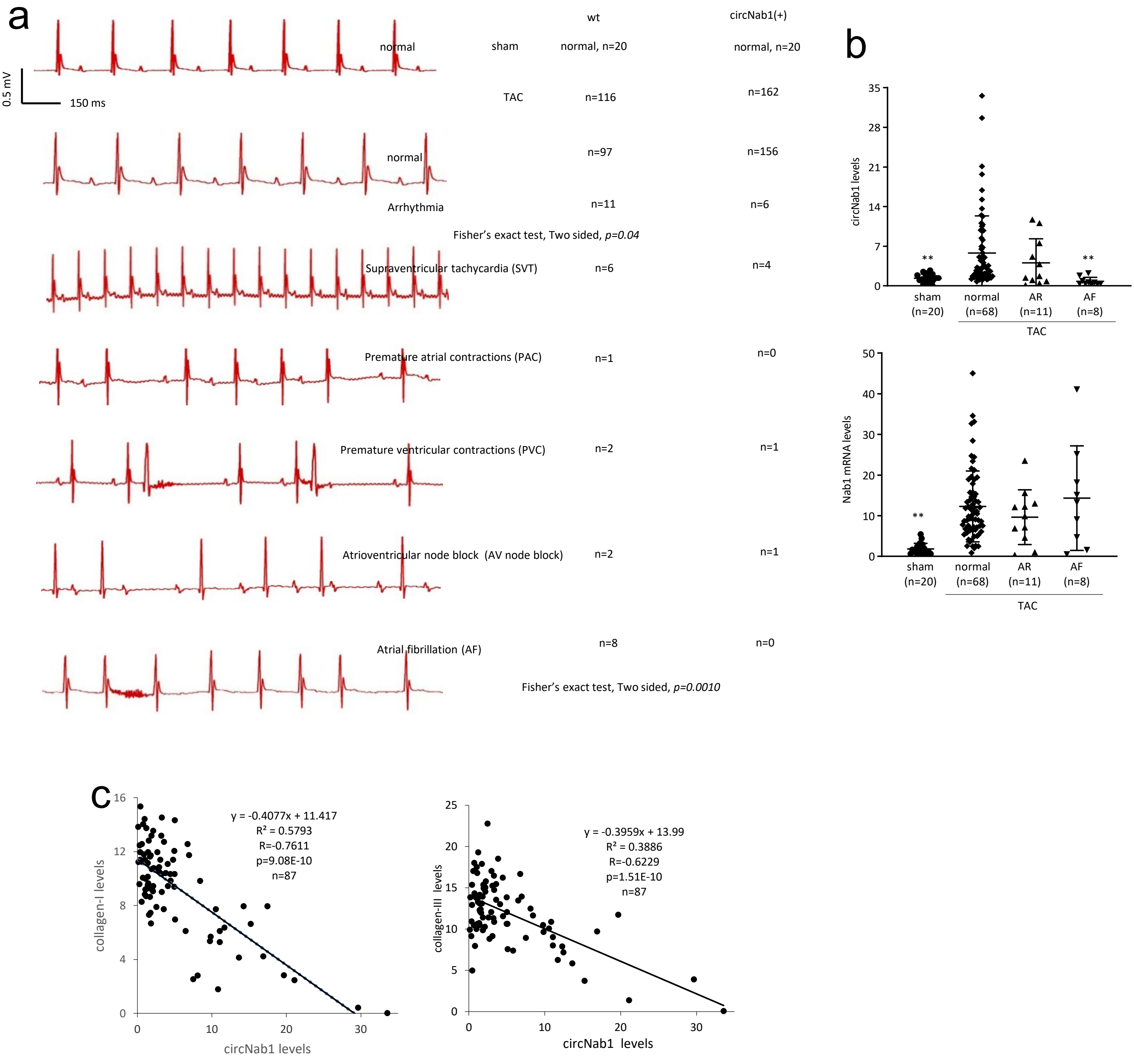

Fig S3. circNAB1(+) mice resisted to TAC induced atrial fibrillation and arial fibrosis.

(a) Eight-week-old wild-type (wt) and circNAB1(+) mice were subjected to pressure overload (PO) via transverse aortic constriction (TAC) for 12 weeks, followed by ECG analysis. Among the 116 wt mice tested, 11 mice were detected with arrhythmia, and 8 mice developed AF. Conversely, among the 162 circNAB1(+) mice, only 6 developed arrhythmia, with no instances of AF detected. The occurrence rate of both arrhythmia and AF in circNAB1(+) mice was significantly lower than wt mice (Fisher's exact test, two-sided, $p<0.05$; *n* labeled in the figures)

(b) Atrial tissues from the aforementioned TAC wt mice were subjected to RT-PCR analysis. circNAB1 expression was significantly increased in TAC mice but not in those with AF (**$p<0.01$ versus normal; *n* was labeled in the figures).

(c) The correlation of collagen-I (left) and collagen-III (right) with circNAB1 was analyzed in 87 wt mouse atrial specimens following TAC. Pearson correlation was performed to evaluate the association, with trend lines, R2, and p values labeled in the figures.

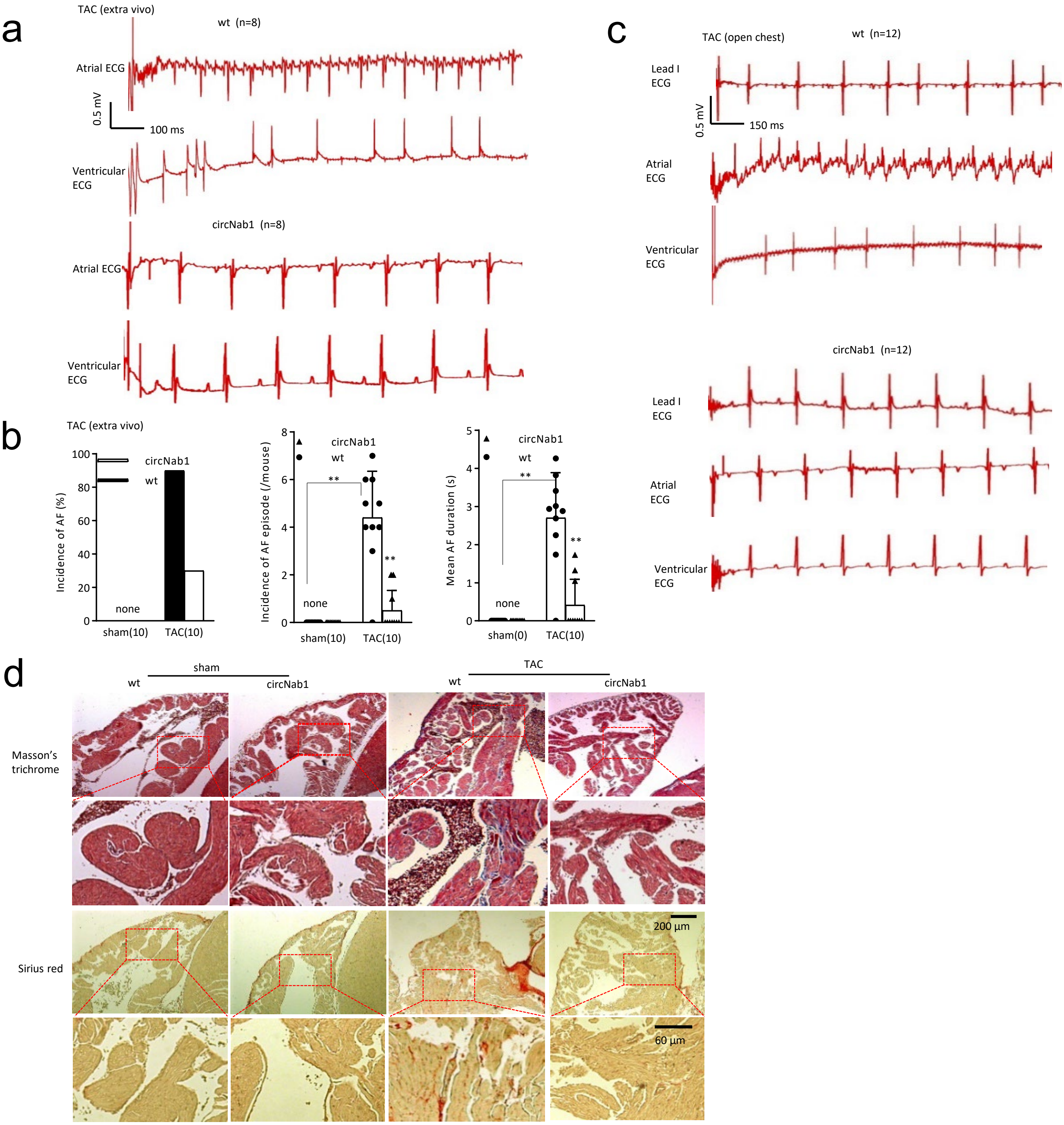

Fig S4. Incidence of AF and fibrosis affected by circNAB1

(A) Eight-week-old wild-type (wt) and circNAB1(+) mice underwent TAC for 12 weeks, followed by programmed electrical stimulation of the right atrium of the TAC-treated mouse hearts under Langendorff-perfusion. Typical images recorded atrial and ventricular ECG after termination of the burst of atrial stimulation (*n=8*).

(B) ECG analysis showed that circNAB1(+) mice exhibited a lower incidence of AF, fewer AF episode, and shorter mean AF duration compared to wt mice during three series of bursts (***p<0.01* versus wt; *n=8*).

(C) Above wt and circNAB1(+) mice were subjected to open-chest programmed electrical stimulation of the right atrium, with ECG recording lead I, atrial, and ventricular electrical signals. Typical images recorded lead I, atrial and ventricular ECG after termination of the burst of atrial stimulation (*n=12*).

(D) Representative images of Masson trichrome and Sirius red staining showed atrial fibrosis. circNAB1(+) mice exhibited decreased Masson's trichrome and Sirius red staining in atrium compared to wt after TAC.

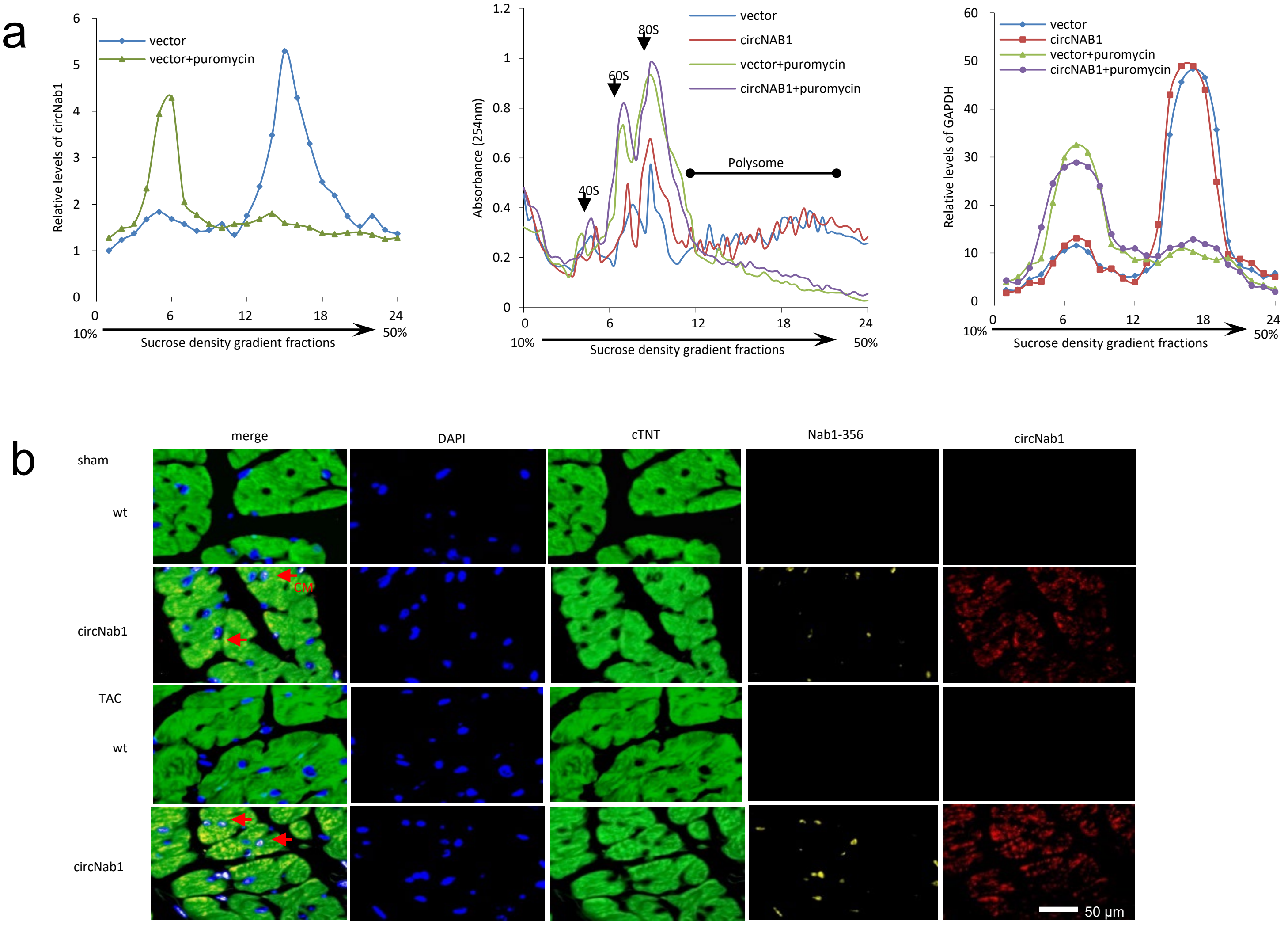

Fig S5. Translation of circNAB1

(a) Left, Lysates prepared from vector- and circNAB1-transfected 293T cells were subjected to sucrose gradient assays. The sucrose gradient assay results of vector-transfected samples were analyzed separately to observe endogenous circNAB1 expression in polysome fractions, which shifted to lighter polysomes after puromycin treatment. Middle, To analyze the polysome distribution, above fractioned lysates were measured for absorbance at 254 nm (A254nm) using a spectrophotometer. Right, PCR of the separated RNAs showed the distribution of GAPDH in polysome fractions, which also shifted to lighter polysomes with puromycin treatment.

(b) In situ hybridization and immunofluorescence staining showed that circNAB1 (red) and NAB1-356 (yellow) were expressed in cardiomyocytes (CM, red arrows) of circNAB1(+) mouse atriums. circNAB1 was expressed in cytosol, while NAB1-356 was expressed in nuclei.

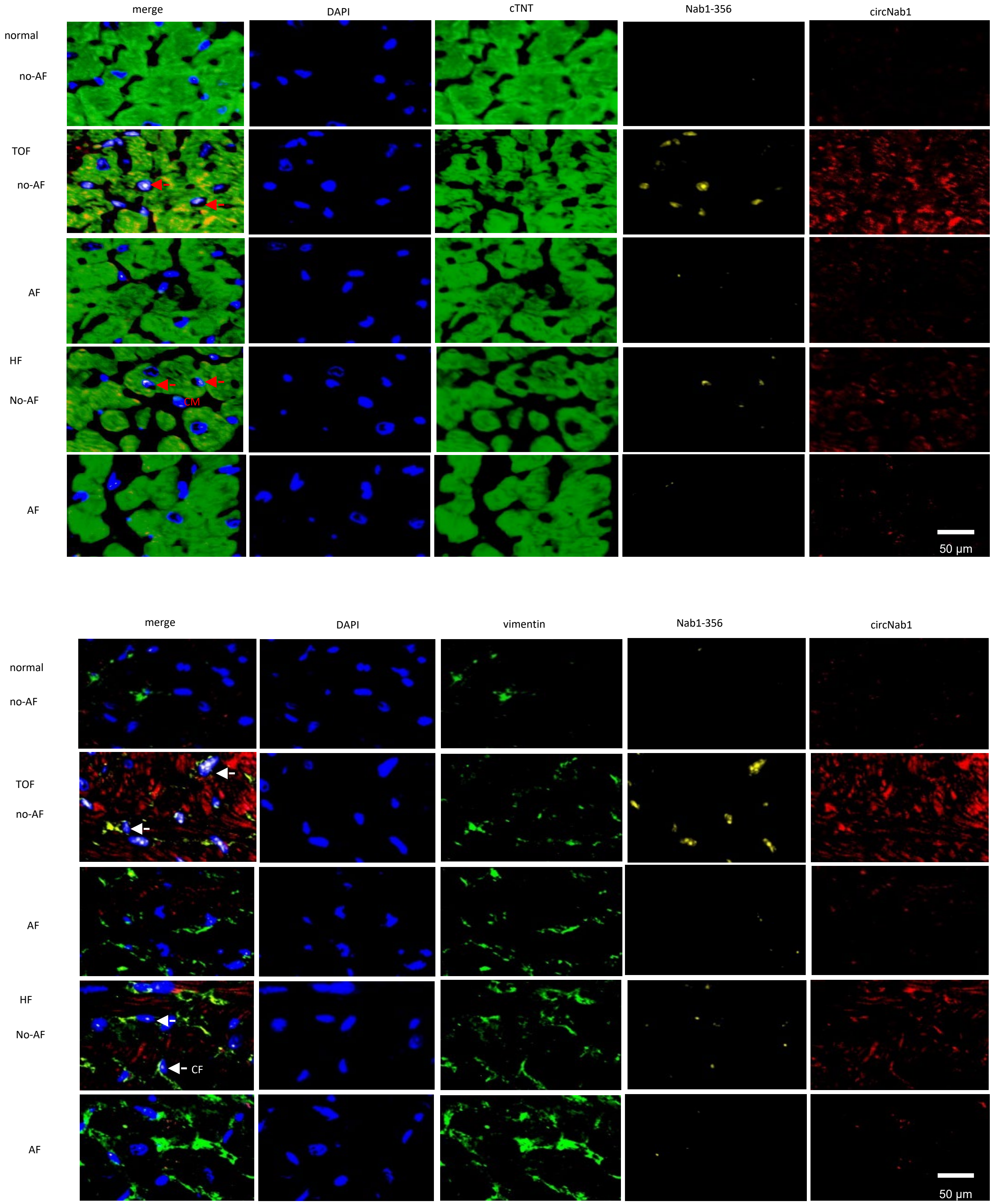

Fig S6. circNAB1 and NAB1-356 expression decreased in AF.
In situ hybridization and immunofluorescence staining showed that circNAB1 (red) and NAB1-356 (yellow) were expressed in cardiomyocytes (upper, CM, red arrows) and cardiac fibroblasts (lower, CF, white arrow) of human TOF and HF atriums with no-AF. circNAB1 was expressed in cytosol, while NAB1-356 was expressed in nuclei.

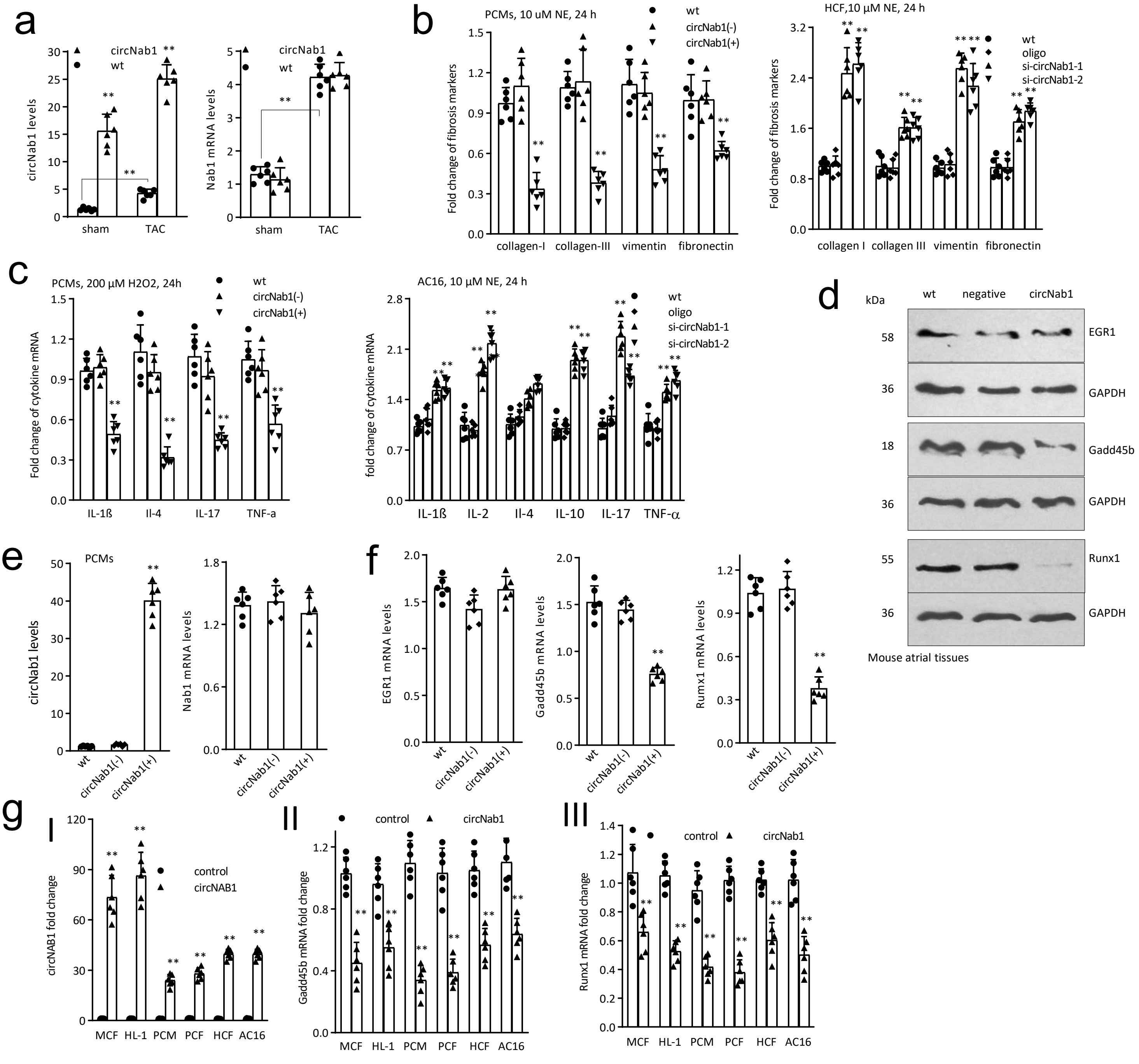

Fig S7. Ectopic expression of circNAB1 altered expression of fibrotic markers and cytokines.

(a). PCR showed that circNAB1(+) mouse atriums expressed high levels of circNAB1 (***p<0.01* versus wt; *n=6*).

(b) Left, PCR showed that circNAB1-expressing PCMs displayed decreased fibrosis markers collagen-I, collagen-III, fibronectin and vimentin (***p<0.01* versus wt; *n=6*). Right, PCR showed that silencing circNAB1 with siRNAs enhanced collagen-I, collagen-III, fibronectin and vimentin expression in HCFs (***p<0.01* versus oligo; *n=6*).

(c) Left, PCR showed that circNAB1-expressing PCMs displayed decreased cytokine IL-1β, Il-2, Il-4, Il-10, Il-17, and TNF-α after culture in basal medium with 200 μM H_2O_2 for 24h (***p<0.01* versus wt; *n=6*). Right, RT-PCR showed that silencing circNAB1 with siRNAs enhanced cytokine levels of IL-1β, Il-2, Il-4, Il-10, Il-17, and TNF-α levels in AC16 cells (***p<0.01* versus oligo; *n=6*).

(d) Western blot showed that circNAB1(+) mouse atria expressed decreased levels of Gadd45b and Runx1 compared to wt mice.

(e) PCMs were isolated from wt, circNAB1(-) and circNAB1(+) mouse hearts and subjected to RT-PCR, showing that circNAB1(+) mice expressed high levels of circNAB1 (***p<0.01* versus wt; *n=6*).

(f) PCR showed that circNAB1 down-regulated Gadd45b and Runx1 expression in PCMs (***p<0.01* versus wt; *n=6*).

(g) I. Mouse heart cell MCF, HL-1, PCM, PCF and human heart cell HCF, AC16 were transiently transduced with circNAB1 using AAV9 as a vector. PCR showed overexpression of circNAB1 in these heart cells. (***p<0.01* versus control; *n=6*). PCR also showed that the expression of circNAB1 suppressed Gadd45b (II) and Runx1 (III) transcription in both mouse and human heart cells (***p<0.01* versus control; *n=6*).

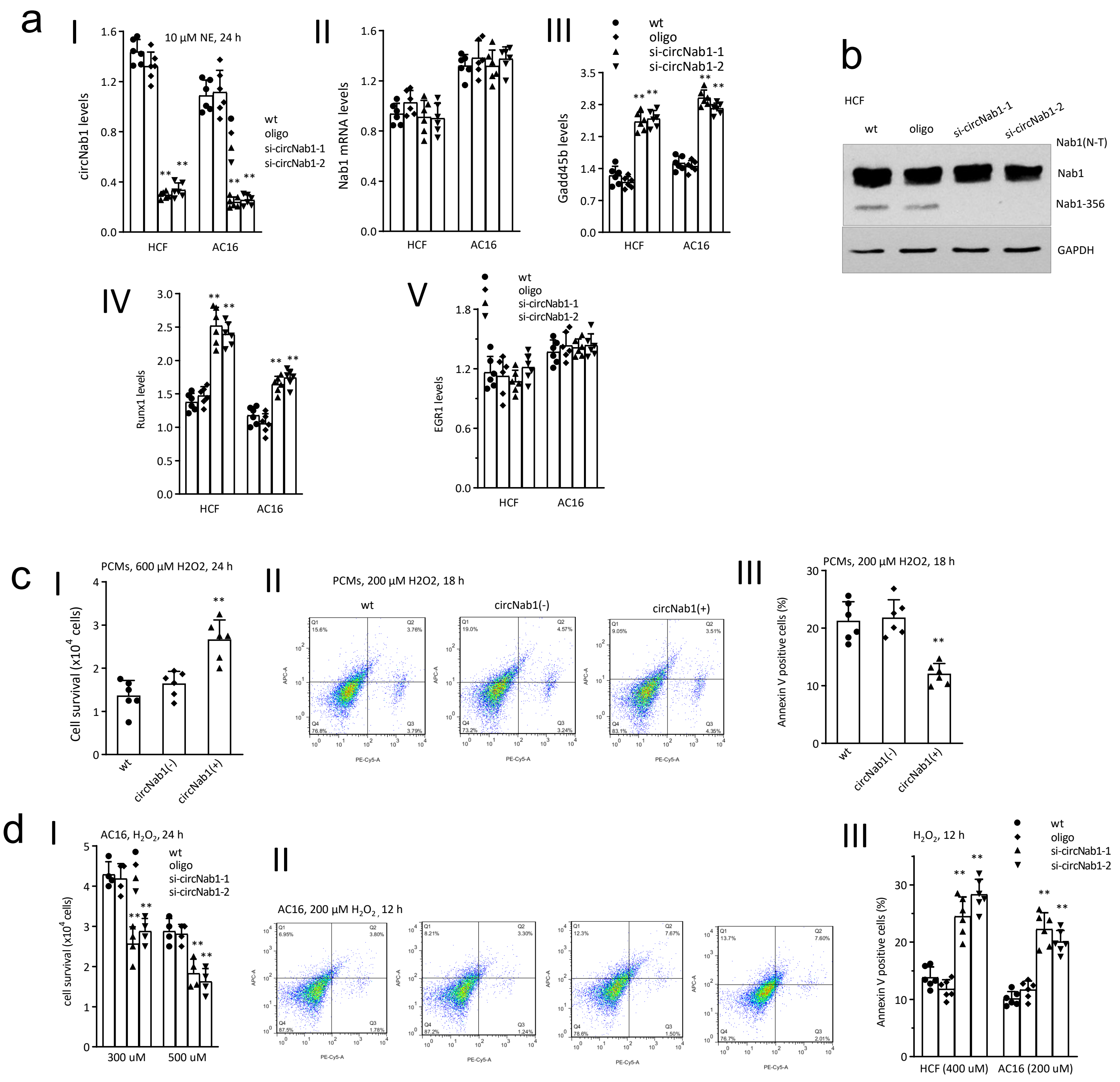

Fig S8. Ectopic expression of circNAB1 altered cell activities.

(a) PCR showed that silencing circNAB1 with siRNAs down-regulated circNAB1 expression in human HCFs and AC16 cell line (I) but didn't change NAB1 levels (II) (***p<0.01* versus oligo; *n=6*). RT-PCR showed that silencing circNAB1 with siRNAs enhanced Gadd45b (III) and Runx1 (IV) expression but did not affect EGR1 levels (V) (***p<0.01* versus oligo; *n=6*).

(b) Silencing activity of circNAB1 was also confirmed by Western blotting, showing decreased levels of NAB1-356.

(c) I. Expression of circNAB1 enhanced PCM survival after cultured in basal medium with 600 μM H_2O_2 for 24 h (***p<0.01* versus wt; *n=6*). II. PCMs were cultured in basal medium with 200 μM H_2O_2 for 18 h, stained with Annexin V, and subjected to flow cytometry. Typical images showed circNAB1 expressing PCMs exhibited decreased Annexin V staining. III. Flow cytometry analysis showed that the expression of circNAB1 suppressed PCM apoptosis (***p<0.01* versus wt; *n=6*).

(d) I. Silencing circNAB1 with siRNAs supressed AC16 cell survival after cultured in basal medium with H_2O_2 for 24h (***p<0.01* versus oligo; *n=6*). II, AC16 cells were cultured in basal medium with 200 μM H_2O_2 for 12 h, stained with Annexin V, and subjected to flow cytometry. Typical images showed siRNA transfected cells showed increased Annexin V staining. III, Flow cytometry analysis showed silencing circNAB1 with siRNAs increased cell apoptosis (***p<0.01* versus oligo; *n=6*).

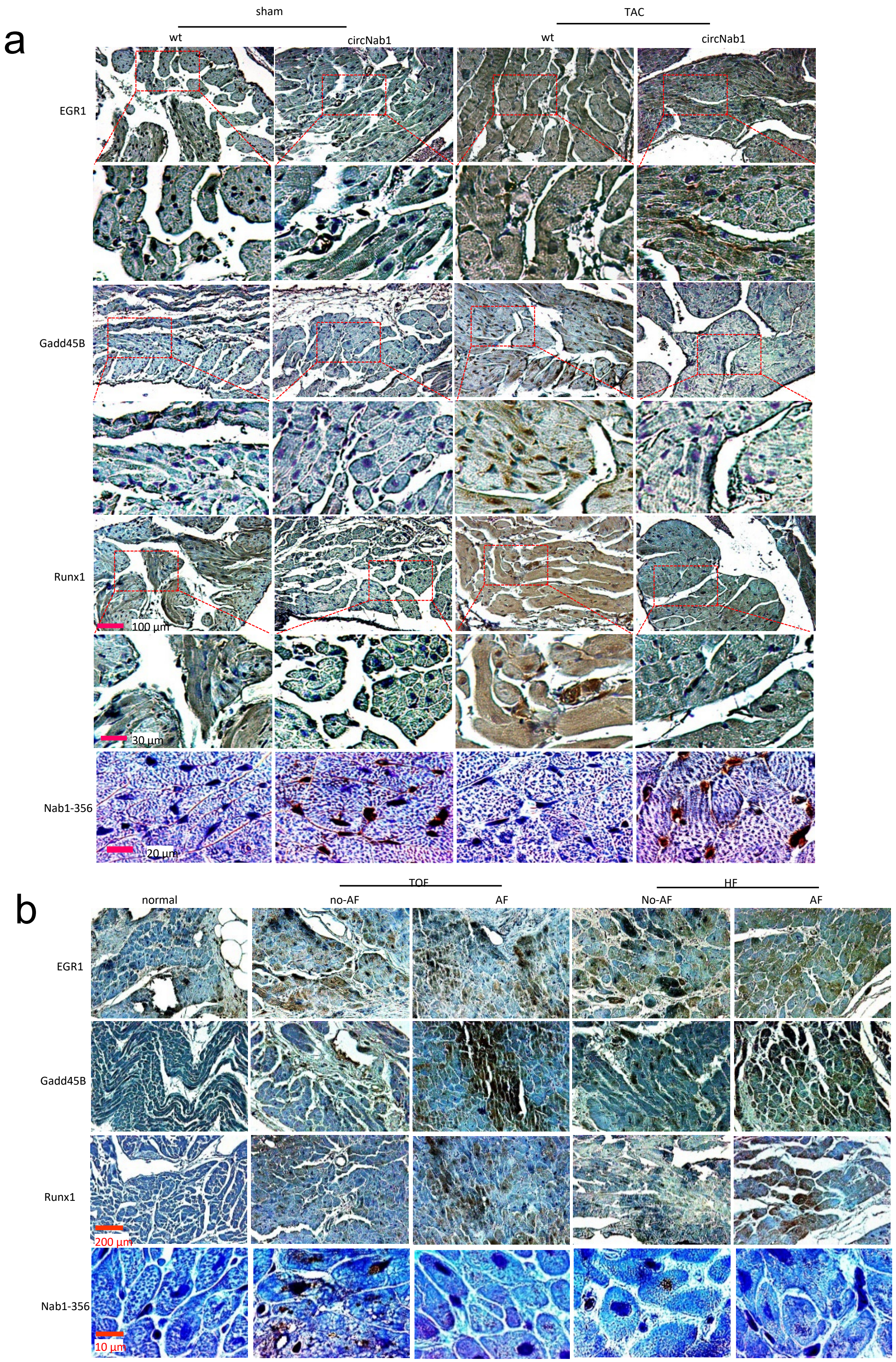

Fig S9. Effect of NAB1-356 on EGR, Gadd45b, and Runx1 expression

(a) Typical IHC staining images showed expression of EGR, Gadd45b, Runx1, and NAB1-356 (using anti-NAB1-356 antibody) in wt and circNAB1(+) mouse atrium slides with TAC.

(b) Typical IHC staining images showed the expression of EGR, Gadd45b, Runx1, and NAB1-356 in atriums of normal and TOF or HF patients with or without AF.

CP75-hu-circNAB1-actin100-2CMV(SalI)

gtcgacacacttagccgtgttctttgcactttctgcatgtcccccgtctggcctggctgtccccagtggcttccccagtgtgacatggtgcatctctgccttacagGttaaacccatccagagtaatggctgcggccttacccaggaccctgggggagttgcagctgtatagaatattacaaaaagccaatctactttcttattttgat
gcctttatccaacaaggtggtgatgatgtccagcaactctgtgaagcaggagaagaggagtttttggaaatcatggcactcgtgggcatggctagcaagccccttcatgttagaaggctgcagaaggctttgagagactgggtcacaaaccctgggcttttcaatcagccactgacttcccttcctgtcagtagcatacccat
ctataaattaccagagggatcaccaacatggctgggaatatcctgcagtagttatgaaaggagtagcaatgcccgggaacctcatttaaaaatccccaaatgtgctgccaccacctgtgtgcagagcttgggacaggggaagtcagatgtggttgggagcctagcactgcagagtgttggtgagtccagactctggcaa
ggccaccatgccactgagagcgagcacagcctctccccagcagacctgggctcccccgcgtccccaaaggagagcagtgaggcgctggatgctgctgctgcgctctctgtggctgagtgtgtggagcggatggcccccacactgccaaaaagtgacttgaatgaagtgaaagagctgctaaaaaccaacaagaa
gttggccaaaatgattggtcacatctttgagatgaacgatgatgatccacacaaagaggaggaaattcggaaatacagtgcaatatatggcagatttgactcaaagaggaaggatgggaaacatctcacacttcatgagctcactgttaatgaagcggctgctcaactctgtgtgaaggataatgccctgctgacaagaa
gagatgagctttttgccttggctcgacagatttctcgagaagtcacctataaatatacttacagaaccaccaagtcaaaatgtggagaaagagatgaattatccccaaagagaattaaagtggag
gtgagtggcccgctacctcttctggtggccgcctccctccttcctggcctcccggagctgcgccctttctcactggttctctcttctgccgttttccgtaggatcc(BamHI)

CP78.NAB1+1b-pcDNA3.1

Gccgccaccatggctgcggccttacccaggaccctgggggagttgcagctgtatagaatattacaaaaagccaatctactttcttattttgatgcctttatccaacaaggtggtgatgatgtccagcaactctgtgaagcaggagaagaggagtttttggaaatcatggcactcgtgggcatggctagcaagccccttcatgtt
agaaggctgcagaaggctttgagagactgggtcacaaaccctgggcttttcaatcagccactgacttcccttcctgtcagtagcatacccatctataaattaccagagggatcaccaacatggctgggaatatcctgcagtagttatgaaaggagtagcaatgcccgggaacctcatttaaaaatccccaaatgtgctgcc
accacctgtgtgcagagcttgggacaggggaagtcagatgtggttgggagcctagcactgcagagtgttggtgagtccagactctggcaaggccaccatgccactgagagcgagcacagcctctccccagcagacctgggctcccccgcgtccccaaaggagagcagtgaggcgctggatgctgctgctgcgctct
ctgtggctgagtgtgtggagcggatggcccccacactgccaaaaagtgacttgaatgaagtgaaagagctgctaaaaaccaacaagaagttggccaaaatgattggtcacatctttgagatgaacgatgatgatccacacaaagaggaggaaattcggaaatacagtgcaatatatggcagatttgactcaaagagg
aaggatgggaaacatctcacacttcatgagctcactgttaatgaagcggctgctcaactctgtgtgaaggataatgccctgctgacaagaagagatgagctttttgccttggctcgacagatttctcgagaagtcacctataaatatacttacagaaccaccaagtcaaaatgtggagaaagagatgaattatccccaaag
agaattaaagtggagGttaaacccatccagagtaatggctgcggccttacccaggaccctgggggagttgcagctgtatag GGATCC

MAAALPRTLGELQLYRILQKANLLSYFDAFIQQGGDDVQQLCEAGEEEFLEIMALVGMASKPLHVRRLQKALRDWVTNPGLFNQPLTSLPVSSIPIYKLPEGSPTWLGISCSSYERSSNAREPHLKIPKCAATTCVQ
SLGQGKSDVVGSLALQSVGESRLWQGHHATESEHSLSPADLGSPASPKESSEALDAAAALSVAECVERMAPTLPKSDLNEVKELLKTNKKLAKMIGHIFEMNDDDPHKEEEIRKYSAIYGRFDSKRKDGKHLTLH
ELTVNEAAAQLCVKDNALLTRRDELFALARQISREVTYKYTYRTTKSKCGERDELSPKRIKVEVKPIQSNGCGLTQDPGGVAAV*

CP95-hu-circNAB1-heart

ccagttgttcaactcacccttcagattaaaaataactgaggtaagggcctgggtaggggaggtggtgtgagacgctcctgtctctcctctatctgcccatcggccctttggggaggaggaatgtgcccaaggactaaaaaaaggccatggagccagaggggcgagggcaacagacctttcatgggcaaaccttggggc
cctgctgtcctcctgtcacctccagagccaagggatcaaaggaggaggagccaggacaggagggaagtgggagggagggtcccagcagaggactccaaatttaggcagcaggcatatgggatgggatataaaggggctggagcactgagagctgtcagagatttctccaaccc
gtcgacacacttagccgtgttctttgcactttctgcatgtcccccgtctggcctggctgtccccagtggcttccccagtgtgacatggtgcatctctgccttacagGttaaacccatccagagtaatggctgcggccttacccaggaccctgggggagttgcagctgtatagaatattacaaaaagccaatctactttcttattttgat
gcctttatccaacaaggtggtgatgatgtccagcaactctgtgaagcaggagaagaggagtttttggaaatcatggcactcgtgggcatggctagcaagccccttcatgttagaaggctgcagaaggctttgagagactgggtcacaaaccctgggcttttcaatcagccactgacttcccttcctgtcagtagcatacccat
ctataaattaccagagggatcaccaacatggctgggaatatcctgcagtagttatgaaaggagtagcaatgcccgggaacctcatttaaaaatccccaaatgtgctgccaccacctgtgtgcagagcttgggacaggggaagtcagatgtggttgggagcctagcactgcagagtgttggtgagtccagactctggcaa
ggccaccatgccactgagagcgagcacagcctctccccagcagacctgggctcccccgcgtccccaaaggagagcagtgaggcgctggatgctgctgctgcgctctctgtggctgagtgtgtggagcggatggcccccacactgccaaaaagtgacttgaatgaagtgaaagagctgctaaaaaccaacaagaa
gttggccaaaatgattggtcacatctttgagatgaacgatgatgatccacacaaagaggaggaaattcggaaatacagtgcaatatatggcagatttgactcaaagaggaaggatgggaaacatctcacacttcatgagctcactgttaatgaagcggctgctcaactctgtgtgaaggataatgccctgctgacaagaa
gagatgagctttttgccttggctcgacagatttctcgagaagtcacctataaatatacttacagaaccaccaagtcaaaatgtggagaaagagatgaattatccccaaagagaattaaagtggag
Gtgagtggcccgctacctcttctggtggccgcctccctccttcctggcctcccggagctgcgccctttctcactggttctctcttctgccgttttccgta GCGGCCGC (NotI)

CP97.circNAB1-stop

gtcgacacacttagccgtgttctttgcactttctgcatgtcccccgtctggcctggctgtccccagtggcttccccagtgtgacatggtgcatctctgccttacagGttaaacccatccagagtaatggctgcggccttacccaggaccctgggggagttgcagctgtatagaatattacaaaaagccaatctactttcttattttgat
gcctttatccaacaaggtggtgatgatgtccagcaactctgtgaagcaggagaagaggagtttttggaaatcatggcactcgtgggcatgTAAagcaagccccttcatgttagaaggctgcagaaggctttgagagactgggtcacaaaccctgggcttttcaatcagccactgacttcccttcctgtcagtagcataccca
tctataaattaccagagggatcaccaacatggctgggaatatcctgcagtagttatgaaaggagtagcaatgcccgggaacctcatttaaaaatccccaaatgtgctgccaccacctgtgtgcagagcttgggacaggggaagtcagatgtggttgggagcctagcactgcagagtgttggtgagtccagactctggcaa
ggccaccatgccactgagagcgagcacagcctctccccagcagacctgggctcccccgcgtccccaaaggagagcagtgaggcgctggatgctgctgctgcgctctctgtggctgagtgtgtggagcggatggcccccacactgccaaaaagtgacttgaatgaagtgaaagagctgctaaaaaccaacaagaa
gttggccaaaatgattggtcacatctttgagatgaacgatgatgatccacacaaagaggaggaaattcggaaatacagtgcaatatatggcagatttgactcaaagaggaaggatgggaaacatctcacacttcatgagctcactgttaatgaagcggctgctcaactctgtgtgaaggataatgccctgctgacaagaa
gagatgagctttttgccttggctcgacagatttctcgagaagtcacctataaatatacttacagaaccaccaagtcaaaatgtggagaaagagatgaattatccccaaagagaattaaagtggaggtgagtggcccgctacctcttctggtggccgcctccctccttcctggcctcccggagctgcgccctttctcactggttct
ctcttctgccgttttccgtaggatcc(BamHI)

CP112.linNAB1-stop

gtcgacGttaaacccatccagagtaatggctgcggccttacccaggaccctgggggagttgcagctgtatagaatattacaaaaagccaatctactttcttattttgatgcctttatccaacaaggtggtgatgatgtccagcaactctgtgaagcaggagaagaggagtttttggaaatcatggcactcgtgggcatgTAAa
gcaagccccttcatgttagaaggctgcagaaggctttgagagactgggtcacaaaccctgggcttttcaatcagccactgacttcccttcctgtcagtagcatacccatctataaattaccagagggatcaccaacatggctgggaatatcctgcagtagttatgaaaggagtagcaatgcccgggaacctcatttaaaaat
ccccaaatgtgctgccaccacctgtgtgcagagcttgggacaggggaagtcagatgtggttgggagcctagcactgcagagtgttggtgagtccagactctggcaaggccaccatgccactgagagcgagcacagcctctccccagcagacctgggctcccccgcgtccccaaaggagagcagtgaggcgctgga
tgctgctgctgcgctctctgtggctgagtgtgtggagcggatggcccccacactgccaaaaagtgacttgaatgaagtgaaagagctgctaaaaaccaacaagaagttggccaaaatgattggtcacatctttgagatgaacgatgatgatccacacaaagaggaggaaattcggaaatacagtgcaatatatggcag
atttgactcaaagaggaaggatgggaaacatctcacacttcatgagctcactgttaatgaagcggctgctcaactctgtgtgaaggataatgccctgctgacaagaagagatgagctttttgccttggctcgacagatttctcgagaagtcacctataaatatacttacagaaccaccaagtcaaaatgtggagaaagagat
gaattatccccaaagagaattaaagtggaggtgagtggcccgctacctcttctggtggccgcctccctccttcctggcctcccggagctgcgccctttctcactggttctctcttctgccgttttccgtaggatcc(BamHI)

CP148.pAAV-GFP-circNAB1, 6867 bp

gggcccgcgttacataacttacggtaaatggcccgcctggctgaccgcccaacgacccccgcccattgacgtcaataatgacgtatgttcccatagtaacgccaatagggactttccattgacgtcaatgggtggagtatttacggtaaactgcccacttggcagtacatcaagtgtatcatatgccaagtacgccccctatt
gacgtcaatgacggtaaatggcccgcctggcattatgcccagtacatgaccttatgggactttcctacttggcagtacatctacgtattagtcatcgctattaccatggtgatgcggttttggcagtacatcaatgggcgtggatagcggtttgactcacggggatttccaagtctccaccccattgacgtcaatgggagtttgttttg
gcaccaaaatcaacgggactttccaaaatgtcgtaacaactccgccccattgacgcaaatgggcggtaggcgtgtacggtgggaggtctatataagcagagctggtttagtgaaccgtcagatccgctagcgctaccggactcagatgtcgacacacttagccgtgttctttgcactttctgcatgtcccccgtctggcctgg
ctgtccccagtggcttccccagtgtgacatggtgcatctctgccttacaggttaaacccatccagagtaatggctgcggccttacccaggaccctgggggagttgcagctgtatagaatattacaaaaagccaatctactttcttattttgatgcctttatccaacaaggtggtgatgatgtccagcaactctgtgaagcaggaga
agaggagtttttggaaatcatggcactcgtgggcatggctagcaagccccttcatgttagaaggctgcagaaggctttgagagactgggtcacaaaccctgggcttttcaatcagccactgacttcccttcctgtcagtagcatacccatctataaattaccagagggatcaccaacatggctgggaatatcctgcagtagtta
tgaaaggagtagcaatgcccgggaacctcatttaaaaatccccaaatgtgctgccaccacctgtgtgcagagcttgggacaggggaagtcagatgtggttgggagcctagcactgcagagtgttggtgagtccagactctggcaaggccaccatgccactgagagcgagcacagcctctccccagcagacctgggc
tcccccgcgtccccaaaggagagcagtgaggcgctggatgctgctgctgcgctctctgtggctgagtgtgtggagcggatggcccccacactgccaaaaagtgacttgaatgaagtgaaagagctgctaaaaaccaacaagaagttggccaaaatgattggtcacatctttgagatgaacgatgatgatccacacaa
agaggaggaaattcggaaatacagtgcaatatatggcagatttgactcaaagaggaaggatgggaaacatctcacacttcatgagctcactgttaatgaagcggctgctcaactctgtgtgaaggataatgccctgctgacaagaagagatgagctttttgccttggctcgacagatttctcgagaagtcacctataaatat
acttacagaaccaccaagtcaaaatgtggagaaagagatgaattatccccaaagagaattaaagtggaggtgagtggcccgctacctcttctggtggccgcctccctccttcctggcctcccggagctgcgccctttctcactggttctctcttctgccgttttccgtaggatcctagttattaatagtaatcaattacggggtcatt
agttcatagcccatatatggagttccgcgttacataacttacggtaaatggcccgcctggctgaccgcccaacgacccccgcccattgacgtcaataatgacgtatgttcccatagtaacgccaatagggactttccattgacgtcaatgggtggagtatttacggtaaactgcccacttggcagtacatcaagtgtatcatatg
ccaagtacgccccctattgacgtcaatgacggtaaatggcccgcctggcattatgcccagtacatgaccttatgggactttcctacttggcagtacatctacgtattagtcatcgctattaccatggtgatgcggttttggcagtacatcaatgggcgtggatagcggtttgactcacggggatttccaagtctccaccccattgac
gtcaatgggagtttgttttggcaccaaaatcaacgggactttccaaaatgtcgtaacaactccgccccattgacgcaaatgggcggtaggcgtgtacggtgggaggtctatataagcagagctgtctaga

CP149.pAAV-GFP-full-length-NAB1, 7194 bp

gggcccgcgttacataacttacggtaaatggcccgcctggctgaccgcccaacgacccccgcccattgacgtcaataatgacgtatgttcccatagtaacgccaatagggactttccattgacgtcaatgggtggagtatttacggtaaactgcccacttggcagtacatcaagtgtatcatatgccaagtacgccccctatt
gacgtcaatgacggtaaatggcccgcctggcattatgcccagtacatgaccttatgggactttcctacttggcagtacatctacgtattagtcatcgctattaccatggtgatgcggttttggcagtacatcaatgggcgtggatagcggtttgactcacggggatttccaagtctccaccccattgacgtcaatgggagtttgttttg
gcaccaaaatcaacgggactttccaaaatgtcgtaacaactccgccccattgacgcaaatgggcggtaggcgtgtacggtgggaggtctatataagcagagctggtttagtgaaccgtcagatccgctagcgctaccggactcagat
gtcgacccgccaccatggctgcggccttacccaggaccctgggggagttgcagctgtatagaatattacaaaaagccaatctactttcttattttgatgcctttatccaacaaggtggtgatgatgtccagcaactctgtgaagcaggagaagaggagtttttggaaatcatggcactcgtgggcatggctagcaagccccttc
atgttagaaggctgcagaaggctttgagagactgggtcacaaaccctgggcttttcaatcagccactgacttcccttcctgtcagtagcatacccatctataaattaccagagggatcaccaacatggctgggaatatcctgcagtagttatgaaaggagtagcaatgcccgggaacctcatttaaaaatccccaaatgtgct
gccaccacctgtgtgcagagcttgggacaggggaagtcagatgtggttgggagcctagcactgcagagtgttggtgagtccagactctggcaaggccaccatgccactgagagcgagcacagcctctccccagcagacctgggctcccccgcgtccccaaaggagagcagtgaggcgctggatgctgctgctgcg
ctctctgtggctgagtgtgtggagcggatggcccccacactgccaaaaagtgacttgaatgaagtgaaagagctgctaaaaaccaacaagaagttggccaaaatgattggtcacatctttgagatgaacgatgatgatccacacaaagaggaggaaattcggaaatacagtgcaatatatggcagatttgactcaaag
aggaaggatgggaaacatctcacacttcatgagctcactgttaatgaagcggctgctcaactctgtgtgaaggataatgccctgctgacaagaagagatgagctttttgccttggctcgacagatttctcgagaagtcacctataaatatacttacagaaccaccaagtcaaaatgtggagaaagagatgaattatcccca
aagagaattaaagtggaggatgggtttccagatttccaggattctgtgcaaacactcttccagcaggctagagctaagagtgaagaacttgcagctcttagttcacagcagcctgaaaaggtgatggcaaagcagatggagttcctttgcaaccaagctggctatgagagactgcagcatgccgagaggaggttgtctgc
agggctttacaggcagagctcagaagagcacagtcctaacggcttgacttccgataactcagatggacaaggagaaagacctttgaatctccgaatgcctaatttacagaacagacaaccccatcattttgtggtggatggggagctgagcagactttaccccagtgaggcaaagtcccactcatcagagagccttggg
attttaaaagactaccctcattcagcttttaccttagaaaagaaagtcatcaaaacagagcctgaagattcaagatagaaacaaaaaaaaaaaacaaataatacaaataaagcaatagcatcacaaatttcacaaataaagcatttttttcactgcaggatcc
tagttattaatagtaatcaattacggggtcattagttcatagcccatatatggagttccgcgttacataacttacggtaaatggcccgcctggctgaccgcccaacgacccccgcccattgacgtcaataatgacgtatgttcccatagtaacgccaatagggactttccattgacgtcaatgggtggagtatttacggtaaactg
cccacttggcagtacatcaagtgtatcatatgccaagtacgccccctattgacgtcaatgacggtaaatggcccgcctggcattatgcccagtacatgaccttatgggactttcctacttggcagtacatctacgtattagtcatcgctattaccatggtgatgcggttttggcagtacatcaatgggcgtggatagcggtttgactc
acggggatttccaagtctccaccccattgacgtcaatgggagtttgttttggcaccaaaatcaacgggactttccaaaatgtcgtaacaactccgccccattgacgcaaatgggcggtaggcgtgtacggtgggaggtctatataagcagagctgtctaga

NC67.NAB1-probe

Cccgggaaaccc aagagaattaaagtggagGttaaacccatccagagtacgt aagagaattaaagtggagGttaaacccatccagagtcccc aagagaattaaagtggagGttaaacccatccagagtgggg aagagaattaaagtggagGttaaacccatccagagttttt tttcccgggccc

Fig S10. Sequences of constructs and primers used. (may be overlapped with major source table)

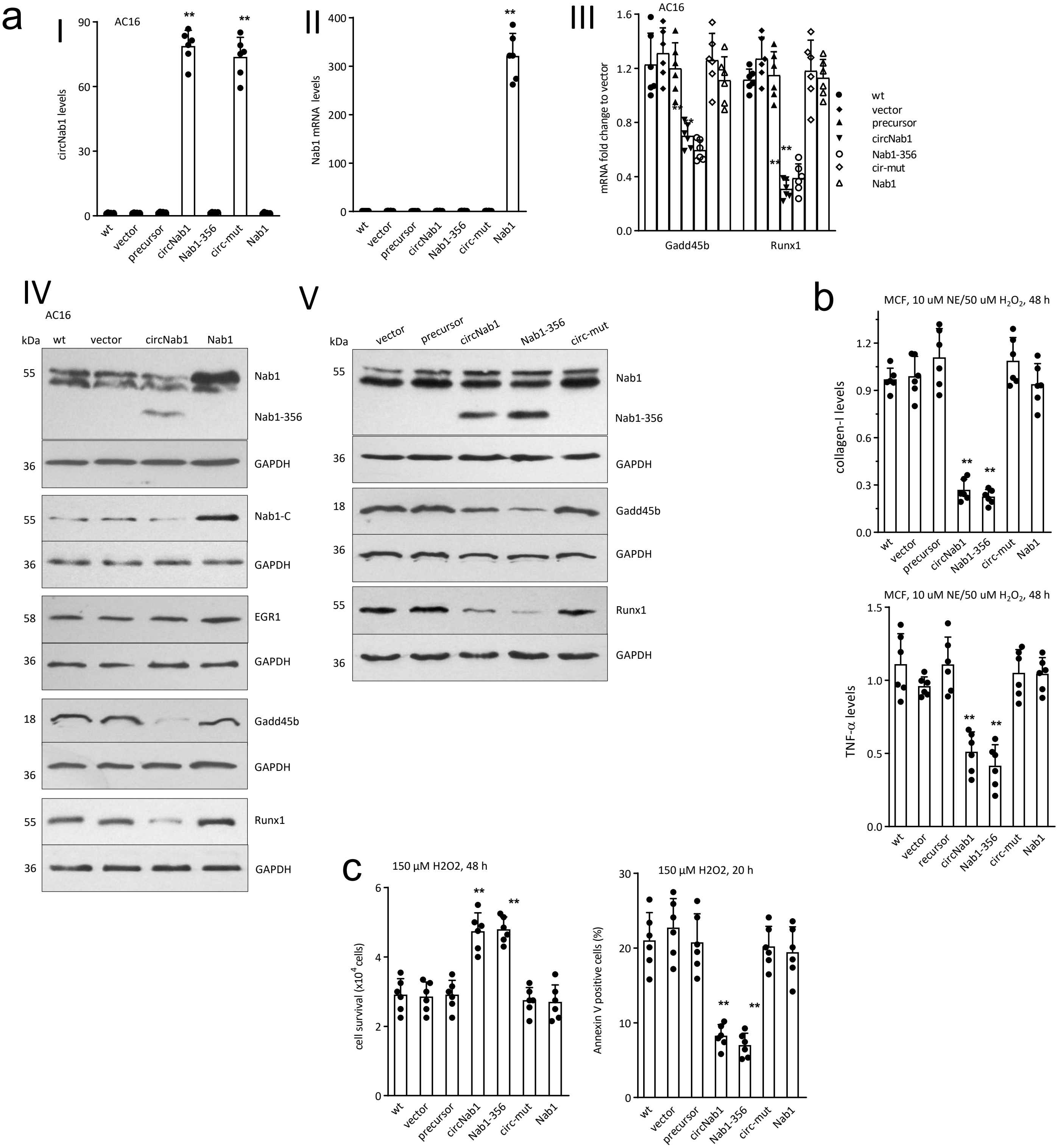

Fig S11. Verification of NAB1-356 on EGR, Gadd45b, and Runx1 expression

(a) I, AC16 cells were transfected with control vector, precursor, circNAB1, NAB1-356, circ-mut and NAB1 plasmid, and subjected to RT-PCR, showing the expression of circNAB1 (I) and NAB1 (II) in the transfected cells (***p<0.01* versus vector; *n=6*). RT-PCR showed that the expression of circNAB1 or NAB1-356 repressed Gadd45b and Runx1 levels (III) (***p<0.01* versus vector; *n=6*). Western blot showed that transfection of circNAB1, not NAB1 suppressed Gadd45b and Runx1 expression (IV). Transfection of circNAB1 and NAB1-356 suppressed Gadd45b and Runx1 protein levels (V).

(b) PCR showed that the expression of circNAB1 or NAB1-356 repressed collagen-I (upper) and TNF-αlevels (lower) in MCFs after the cells were cultured in basal medium with 10 uM NE/50 uM H2O2 for 48 h. (***p<0.01* versus vector; *n=6*).

(c) Left, Transfection of circNAB1 or NAB1-356 enhanced AC16 cell survival after cultured in basal medium with 150 μM H_2O_2 for 48 h. Right, Expression of circNAB1 or NAB1-356 repressed AC16 cell apoptosis (***p<0.01* versus vector; *n=6*).

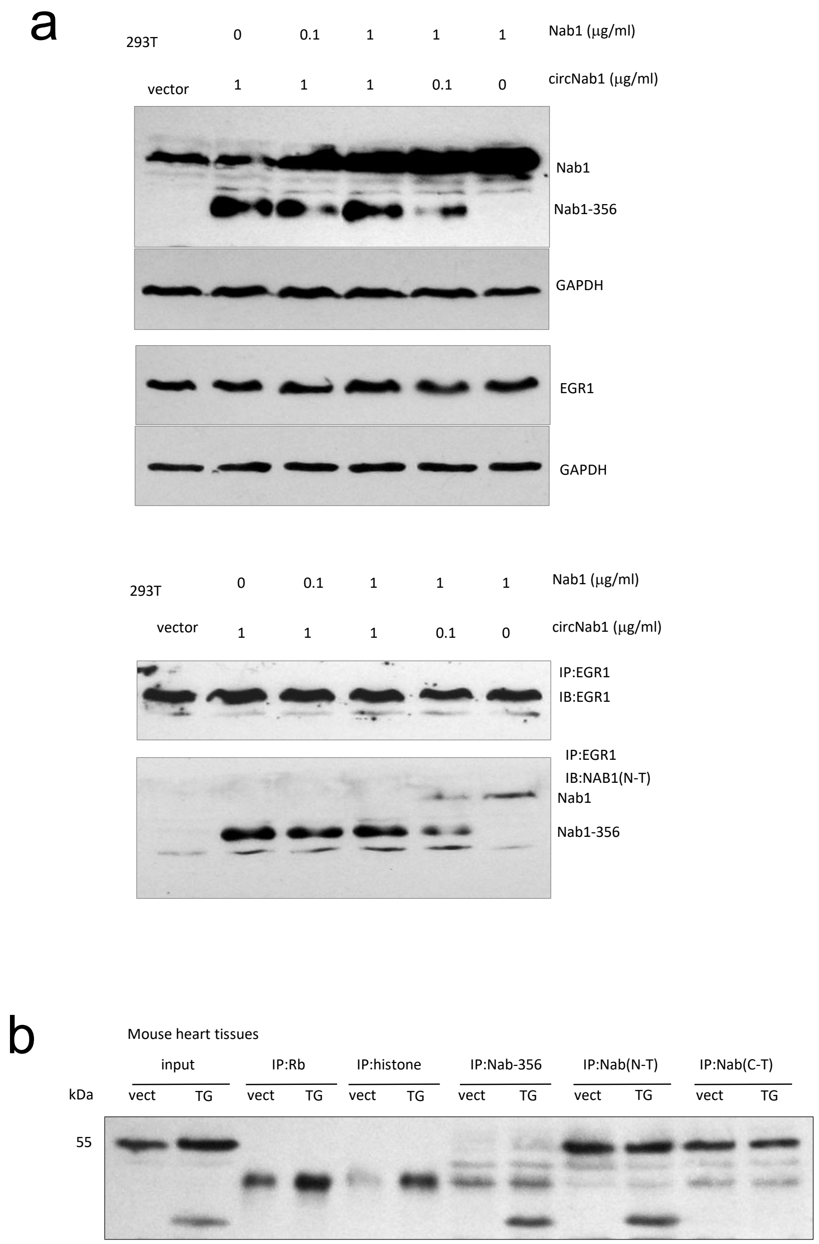

Fig S12. Verification of NAB1-356 on EGR, Gadd45b, and Runx1 expression

(a) Upper, 293T cells were transfected with a control vector, circNAB1 (1 μg/ml), circNAB1 (1 μg/ml) + NAB1 (0.1 μg/ml), circNAB1 (1 μg/ml) + NAB1 (1 μg/ml), circNAB1 (0.1 μg/ml) + NAB1 (1 μg/ml) and NAB1 (1 μg/ml), and subjected to Western blot with antibodies against NAB1 (N-T) and EGR1, showing the expression of NAB1, NAB1-356 and EGR1after transfection.

Lower, Above samples were subjected to immunoprecipitation with antibody against EGR1. Western blot analysis showed that precipitation of EGR1 pulled down more NAB1-356 than NAB1. NAB1-356 seemed to take a more competitive advantage than NAB1 to interact with EGR1.

(b) Chromatin from wt and circNAB1(+) mouse atrial tissues was isolated, digested, and immunoprecipitated with rabbit IgG and antibodies against histone, NAB1-356 (using anti-NAB1-356 antibody), NAB1(N-T) and NAB1(C-T), followed by western blot with antibody against NAB1(N-T).

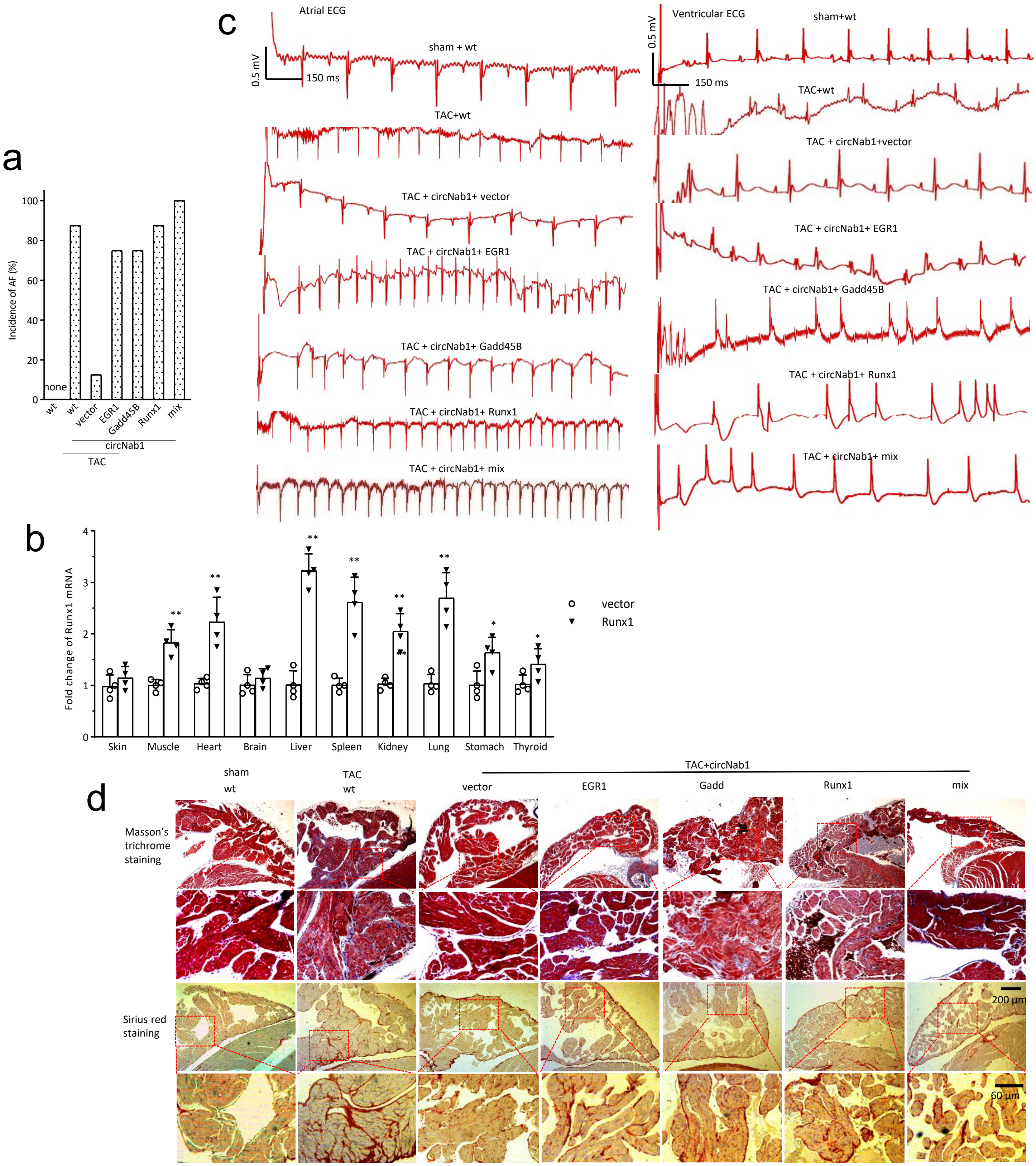

Fig S13. Verification of NAB1-356 by gain of functions

(a) Eight-week-old wild-type (wt) and circNAB1(+) mice were subjected to TAC and injected with EGR1, Gadd45b, Runx1 or a mixture twice a week for 12 weeks. ECG analysis showed that circNAB1(+) mice displayed high incidence of AF after TAC and delivery with EGR1, Gadd45b or Runx1 during 3 series of bursts as well as wt mice (*n=8*). (b) Tissues collected from skin, muscle, heart, brain, liver, spleen, kidney, lung, stomach and thyroid of mice delivered with vector or Runx-1 were lysed and subjected to RT-PCR, showing that expression of Runx-1 with PEG-Au NP increased Runx-1 expression levels in above organs. (*$p<0.05$, **$p<0.01$ versus vector; *n=4*). (c) Mouse hearts were processed to programmed electrical stimulation of right atrium under Langendorff-perfusion. Typical images recorded atrial (left) and ventricular (right) ECG after termination of atrial stimulation (*n=8*). (d) Representative images showed Masson trichrome and Sirius red staining of atriums.

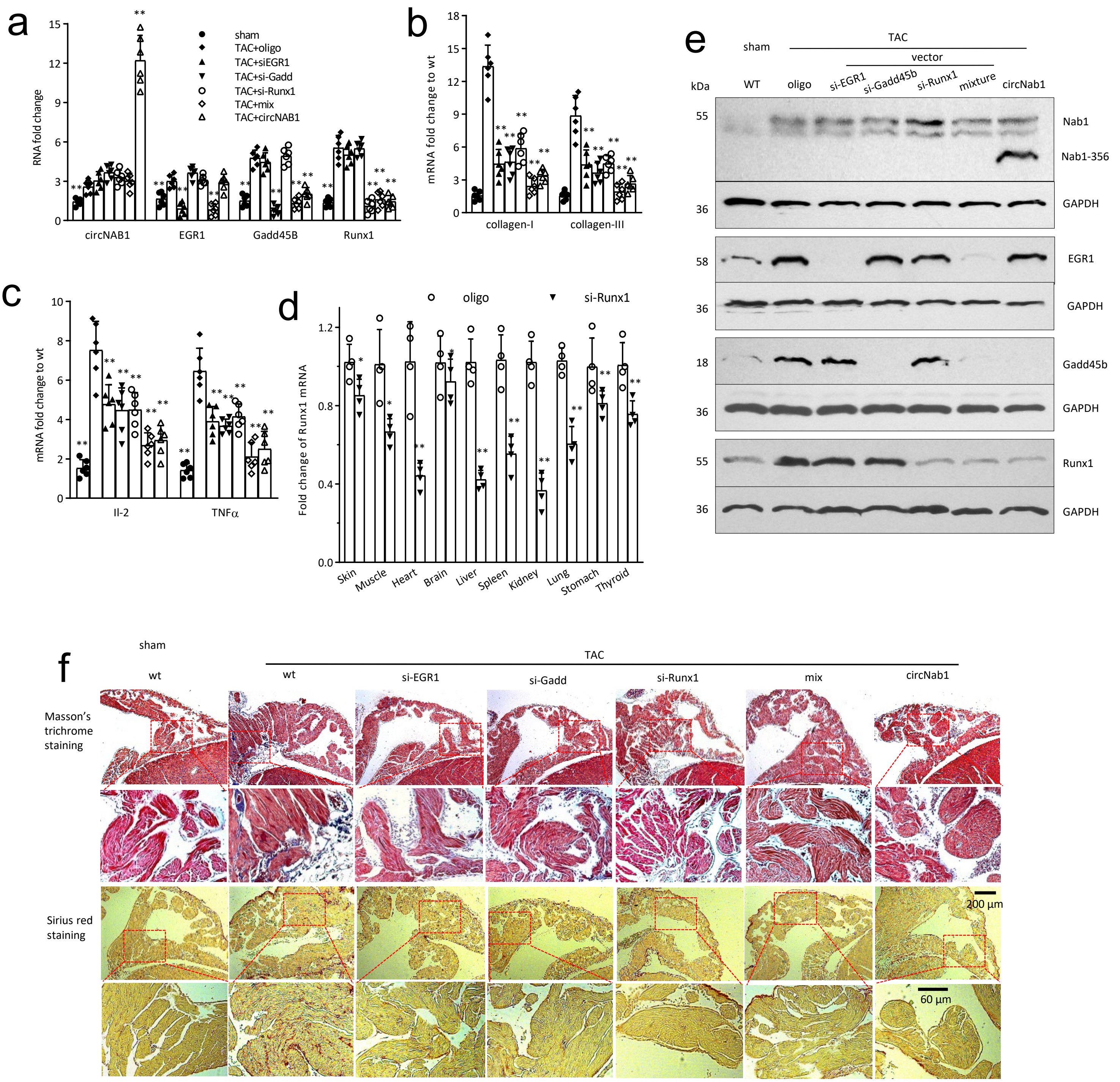

Fig S14. Verification of NAB1-356 by loss of functions

(a) RT-PCR showed delivery of siRNAs against EGR1, Gadd45b or Runx1 decreased EGR1, Gadd45b and Runx1 mRNA in mouse atriums (***$p<0.01$* versus oligo; *$n=6$*).

(b) RT-PCR showed delivery of siRNAs against EGR1, Gadd45b or Runx1 decreased collagen-I and collagen-III mRNA levels in mouse atriums (***$p<0.01$* versus oligo; *$n=6$*).

(c) RT-PCR showed delivery of siRNAs against EGR1, Gadd45b or Runx1 decreased Il-2 and TNF-α mRNA levels in mouse atriums (***$p<0.01$* versus oligo; *$n=6$*).

(d) Tissues collected from skin, muscle, heart, brain, liver, spleen, kidney, lung, stomach and thyroid of mice delivered with oligo or si-Runx-1were lysed and subjected to RT-PCR, showing that silencing of Runx-1 with siRNAs decreased Runx-1 expression in above organs. (**$p<0.05$, **$p<0.01$* versus oligo; *$n=4$*).

(e) Western blot showed delivery of siRNAs against EGR1, Gadd45b or Runx1 decreased EGR1, Gadd45b and Runx1 expression in mouse atriums.

(f) Representative images showed Masson trichrome and Sirius red staining of atriums.

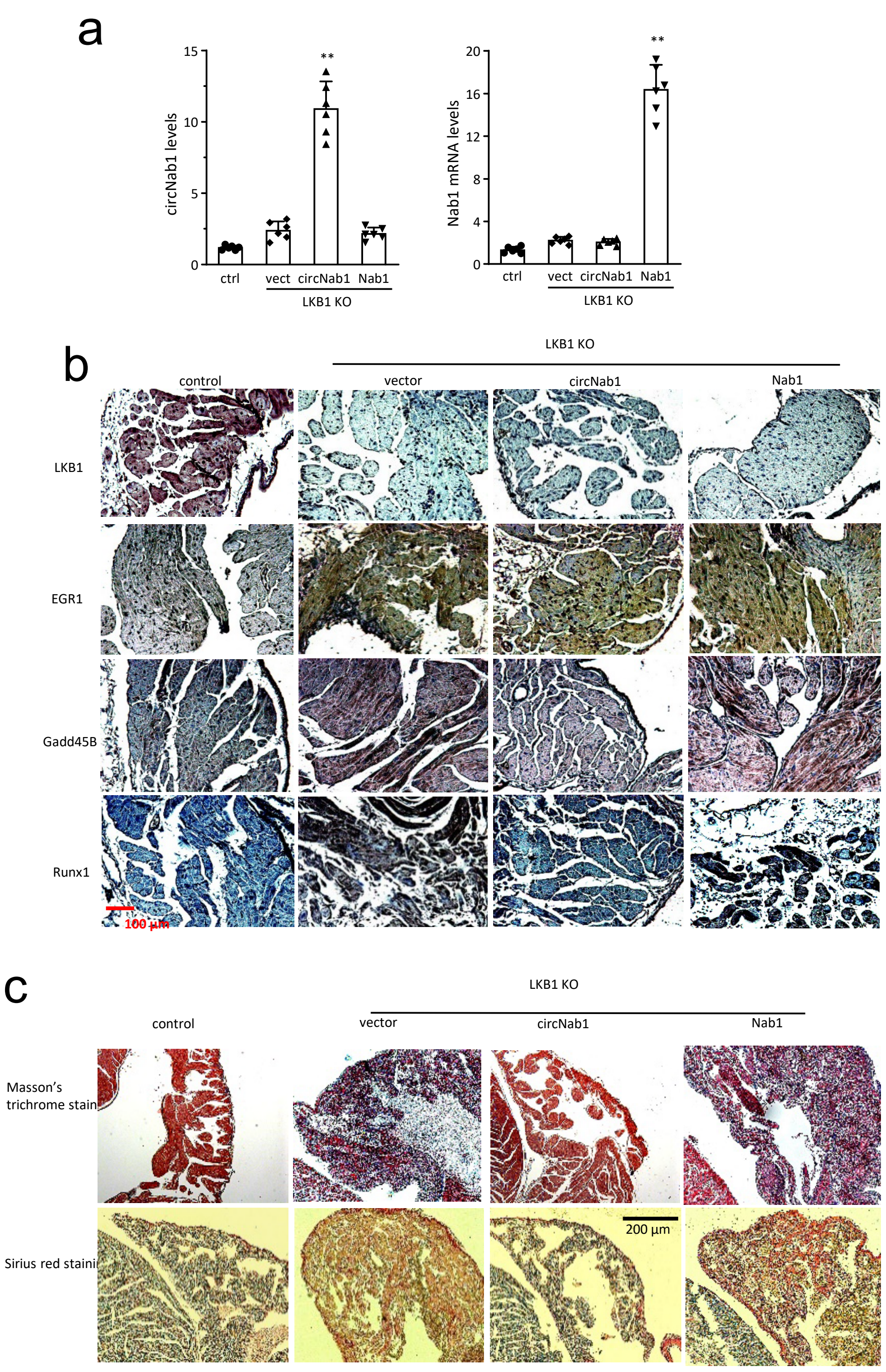

Fig S15. Verification of NAB1-356 in LKB1 KO mice.
(a)PCR showed expression of circNAB1 and NAB1 mRNA in the mouse atriums (***p<0.01* versus vector; *n=6*).
(b) Typical IHC staining images showed expression of EGR, Gadd45b and Runx1 in the mouse atriums.
(c) Representative images showed Masson trichrome and Sirius red staining of atriums.

Supplementary Table S1-RNA-Seq-2

geneid	chr	name	circNab1.1	circNab1.3	circNab1.4	WT2	WT3	WT4	circNab1.1_FPKM	circNab1.3_FPKM	circNab1.4_FPKM	WT2_FPKM	WT3_FPKM	WT4_FPKM	baseMean	log2FC	Pvalue
ENSMUSG00000064351	MT	mt-Co1	2170375.4	2281326.8	2556230.2	1697951.1	1979733.7	2021719.2	63823.6	61640.1	68356.7	53104.8	59224.6	59459.6	2117889.4	0.29818	0.001375441
ENSMUSG00000064370	MT	mt-Cytb	981374.3	1219615.7	1334767.4	859413.4	909751.6	928109.6	38974.8	44504.3	48204.7	36300.6	36755.4	36864.0	1038838.6	0.39051	0.000188481
ENSMUSG00000064341	MT	mt-Nd1	756151.4	905772.3	976870.5	642034.2	717506.5	771525.5	35898.1	39510.4	42173.0	32417.8	34652.8	36632.6	794976.7	0.30830	0.003047945
ENSMUSG00000064363	MT	mt-Nd4	438787.2	515085.8	607294.9	380778.4	387927.8	383304.9	14467.0	15604.0	18207.9	13352.4	13011.5	12639.3	452196.5	0.43847	2.98E-05
ENSMUSG00000064345	MT	mt-Nd2	384459.0	423600.5	454056.3	335468.6	413942.6	423949.1	16827.8	17035.8	18072.7	15616.8	18431.8	18558.6	405912.7	0.10519	0.300290227
ENSMUSG00000064367	MT	mt-Nd5	271475.4	290244.1	315475.4	257205.3	273765.2	279023.6	6762.1	6642.7	7145.8	6813.8	6937.1	6951.0	281198.2	0.11498	0.153004605
ENSMUSG00000064368	MT	mt-Nd6	71772.4	97320.9	124461.8	59621.5	65195.4	63884.8	6283.0	7827.9	9907.8	5551.0	5806.0	5593.2	80376.1	0.63749	7.53E-06
ENSMUSG00000026043	1	Col3a1	9960.1	5974.2	6051.9	26170.2	17632.5	13304.8	81.3	44.8	44.9	227.3	146.5	108.7	13182.3	-1.37703	0.000362042
ENSMUSG00000064354	MT	mt-Co2	12460.2	12840.3	6958.7	4862.1	6215.3	3971.6	827.6	783.7	420.3	343.5	420.0	263.8	7884.7	1.10011	0.000506551
ENSMUSG00000064358	MT	mt-Co3	6839.0	10677.6	8774.8	7087.0	5338.5	6323.8	396.3	568.5	462.4	436.8	314.7	366.5	7506.8	0.48771	0.000475358
ENSMUSG00000029661	6	Col1a2	4326.5	3202.8	3145.5	11255.0	7673.3	6388.9	36.8	25.0	24.3	101.7	66.3	54.3	5998.7	-1.24580	2.18E-05
ENSMUSG00000001506	11	Col1a1	3810.9	2606.6	2313.5	9760.4	6613.2	5301.3	29.2	18.3	16.1	79.5	51.5	40.6	5067.6	-1.31164	0.000164984
ENSMUSG00000064357	MT	mt-Atp6	3898.5	6316.4	5348.0	2890.7	3036.9	6550.5	260.1	387.2	324.5	205.1	206.1	437.1	4673.5	0.31866	0.435370041
ENSMUSG00000019577	6	Pdk4	1408.2	2400.2	2549.0	3375.5	6326.4	7584.6	18.4	28.8	30.2	46.8	83.9	98.9	3940.6	-1.44331	0.000266743
ENSMUSG00000027750	3	Postn	1400.2	1004.3	1037.2	8135.0	5073.2	3569.9	12.2	8.0	8.2	75.3	44.9	31.1	3370.0	-2.28523	1.99E-09
ENSMUSG00000029675	5	Eln	1357.3	1003.3	896.0	3915.6	2751.2	2085.3	16.0	10.9	9.6	49.1	33.0	24.6	2001.4	-1.42591	9.65E-06
ENSMUSG00000042436	11	Mfap4	1071.3	638.8	601.3	3459.0	2173.8	1599.4	27.5	15.1	14.0	94.4	56.8	41.1	1590.6	-1.64521	0.000144397
ENSMUSG00000042254	9	Cilp	396.7	333.5	338.1	4052.4	2334.7	1615.6	4.3	3.4	3.4	47.2	26.0	17.7	1511.8	-2.90493	2.99E-13
ENSMUSG00000021388	13	Aspn	922.1	690.2	728.1	2515.5	1720.3	1281.8	18.0	12.4	12.9	52.2	34.1	25.0	1309.7	-1.23696	7.29E-05
ENSMUSG00000026042	1	Col5a2	1074.9	716.4	724.5	2303.3	1578.9	1215.8	7.4	4.5	4.5	16.8	11.0	8.3	1269.0	-1.01851	0.002599364
ENSMUSG00000032942	7	Ucp3	378.9	849.2	836.8	1011.8	1502.9	1763.9	7.0	14.5	14.1	20.0	28.4	32.7	1057.2	-1.05162	0.013399892
ENSMUSG00000064360	MT	mt-Nd3	1030.2	1216.6	1480.4	801.6	878.7	885.8	134.5	145.9	175.8	111.3	116.7	115.7	1048.9	0.53644	2.80E-05
ENSMUSG00000083863	2	Gm13341	1108.9	1521.9	1607.2	670.9	327.7	815.9	75.0	94.5	98.8	48.2	22.5	55.2	1008.7	1.22363	0.002687199
ENSMUSG00000023034	15	Nr4a1	785.4	1302.8	1206.3	604.5	435.0	405.6	14.0	21.3	19.5	11.4	7.9	7.2	789.9	1.18854	3.44E-11
ENSMUSG00000020473	11	Aebp1	587.0	424.6	355.0	1355.8	956.7	896.3	7.0	4.6	3.8	17.1	11.5	10.6	762.6	-1.22792	6.82E-12
ENSMUSG00000021253	12	Tgfb3	485.2	431.4	423.8	1214.0	805.6	710.7	6.5	5.3	5.2	17.3	11.0	9.5	678.4	-1.02508	1.22E-09
ENSMUSG00000041559	1	Fmod	197.5	154.1	129.2	1469.5	933.3	647.6	2.9	2.1	1.7	22.8	13.8	9.4	588.5	-2.66363	3.51E-12
ENSMUSG00000021702	13	Thbs4	146.5	174.5	179.9	1412.2	895.3	621.8	2.1	2.3	2.3	21.3	12.9	8.8	571.7	-2.54855	3.59E-13
ENSMUSG00000029838	6	Ptn	229.6	147.3	146.1	1223.1	741.2	529.9	4.1	2.4	2.4	23.1	13.4	9.4	502.9	-2.25202	8.40E-08
ENSMUSG00000042985	5	Upk3b	301.1	156.1	147.3	944.5	714.9	602.6	3.9	1.8	1.7	12.9	9.4	7.8	477.7	-1.90143	6.80E-07
ENSMUSG00000031849	8	Comp	258.2	193.9	189.6	953.5	648.5	480.2	4.9	3.3	3.2	19.1	12.4	9.0	454.0	-1.69486	2.53E-16
ENSMUSG00000002020	12	Ltbp2	143.0	140.6	135.2	998.8	585.1	419.9	1.0	0.9	0.8	7.1	4.0	2.8	403.8	-2.25809	9.04E-10
ENSMUSG00000061780	10	Cfd	265.4	108.6	111.1	261.5	737.3	933.6	13.1	4.9	5.0	13.7	37.0	46.0	402.9	-1.99314	0.002528738
ENSMUSG00000035258	16	Abi3bp	295.8	217.1	212.5	718.2	529.6	386.5	2.6	1.7	1.7	6.7	4.7	3.4	393.3	-1.16820	5.84E-09
ENSMUSG00000054675	5	Tmem119	360.1	192.9	152.1	623.6	485.7	359.7	7.3	3.6	2.8	13.4	10.0	7.3	362.3	-1.05684	0.014666938
ENSMUSG00000040152	2	Thbs1	240.4	192.9	207.7	628.6	469.1	399.8	1.9	1.4	1.5	5.2	3.7	3.1	356.4	-1.22221	2.17E-12
ENSMUSG00000040690	4	Col16a1	257.3	204.5	245.1	627.6	435.9	360.6	2.2	1.6	1.9	5.8	3.9	3.1	355.2	-1.00994	8.23E-08
ENSMUSG00000027559	3	Car3	200.1	93.1	117.1	249.4	641.7	815.0	5.4	2.3	2.9	7.2	17.7	22.1	352.7	-2.05495	0.000369936
ENSMUSG00000053310	9	Nrgn	189.4	177.4	136.4	282.6	506.2	659.1	5.9	5.1	3.9	9.4	16.1	20.6	325.2	-1.52323	5.41E-05
ENSMUSG00000031517	8	Gpm6a	246.6	136.7	149.7	484.8	361.8	346.3	3.5	1.8	1.9	7.3	5.2	4.9	287.7	-1.15644	3.67E-08
ENSMUSG00000002289	17	Angptl4	71.5	181.3	177.5	323.9	431.1	470.6	1.0	2.3	2.2	4.7	6.0	6.5	276.0	-1.51258	0.000459433
ENSMUSG00000063415	6	Cyp26b1	612.1	240.4	317.6	137.8	148.2	173.1	6.2	2.2	2.9	1.5	1.5	1.8	271.5	1.35015	0.001552485
ENSMUSG00000066687	9	Zbtb16	165.3	170.6	157.0	268.6	384.2	479.2	1.5	1.4	1.3	2.5	3.5	4.3	270.8	-1.19920	6.98E-10
ENSMUSG00000031673	8	Cdh11	235.9	104.7	154.6	503.9	358.9	247.7	1.9	0.8	1.1	4.3	2.9	2.0	267.6	-1.16421	0.007926955
ENSMUSG00000055653	X	Gpc3	182.3	135.7	113.5	503.9	349.1	299.4	3.7	2.6	2.1	11.0	7.3	6.1	264.0	-1.41007	3.36E-11
ENSMUSG00000063011	17	Msln	136.7	83.4	73.7	468.7	373.5	297.5	2.9	1.6	1.4	10.5	8.0	6.2	238.9	-1.94506	6.24E-18
ENSMUSG00000027996	3	Sfrp2	101.9	83.4	58.0	548.2	329.6	284.1	2.3	1.7	1.2	13.2	7.6	6.4	234.2	-2.24546	9.07E-20
ENSMUSG00000024529	18	Lox	112.6	110.5	78.5	474.7	341.3	238.2	1.1	1.0	0.7	4.8	3.3	2.3	226.0	-1.79830	1.14E-14
ENSMUSG00000065947	MT	mt-Nd4l	196.6	383.9	80.9	268.6	228.2	151.1	30.1	54.0	11.3	43.7	35.5	23.1	218.2	0.03078	0.961907278
ENSMUSG00000027188	2	Pamr1	181.4	91.1	91.8	441.6	287.7	211.4	2.9	1.3	1.3	7.4	4.6	3.4	217.5	-1.36591	0.002530302
ENSMUSG00000038725	15	Pkhd1l1	194.8	113.4	106.3	347.0	295.5	241.1	0.7	0.4	0.3	1.3	1.1	0.8	216.3	-1.08384	1.20E-06
ENSMUSG00000027239	2	Mdk	166.2	110.5	93.0	436.5	290.6	194.2	7.2	4.4	3.6	20.0	12.7	8.4	215.2	-1.31054	6.32E-07
ENSMUSG00000031595	8	Pdgfrl	177.8	96.9	122.0	380.2	238.0	181.7	3.9	2.0	2.4	8.9	5.3	4.0	199.4	-1.00784	0.000111887
ENSMUSG00000028989	4	Angptl7	67.0	86.3	82.1	405.3	262.3	162.6	1.4	1.7	1.6	9.1	5.6	3.4	177.6	-1.82012	1.67E-11
ENSMUSG00000028194	3	Ddah1	102.8	117.3	89.4	306.8	223.3	207.6	1.2	1.3	1.0	3.9	2.7	2.5	174.5	-1.24843	7.90E-10
ENSMUSG00000044006	8	Cilp2	111.7	24.2	15.7	279.6	229.2	252.5	1.2	0.2	0.2	3.1	2.5	2.7	152.2	-2.32313	0.004683206
ENSMUSG00000001131	X	Timp1	76.8	99.8	71.2	286.7	192.1	133.9	4.0	4.7	3.3	15.7	10.1	6.9	143.4	-1.30139	6.32E-07
ENSMUSG00000029372	5	Ppbp	80.4	43.6	38.6	130.8	234.1	320.4	3.4	1.7	1.5	5.8	10.0	13.4	141.3	-2.06349	3.85E-11
ENSMUSG00000024653	19	Scgb1a1	0.0	0.0	0.0	0.0	357.9	488.8	0.0	0.0	0.0	0.0	34.8	46.7	141.1	-10.56169	0.006867054
ENSMUSG00000025479	7	Cyp2e1	83.1	57.2	27.8	82.5	223.3	316.6	2.1	1.3	0.6	2.2	5.6	7.8	131.7	-1.88516	0.005432478
ENSMUSG00000056174	4	Col8a2	55.4	32.0	32.6	321.9	205.8	131.0	0.6	0.3	0.3	3.6	2.2	1.4	129.8	-2.44509	4.56E-15
ENSMUSG00000034664	11	Itga2b	91.1	58.2	51.9	128.7	197.0	220.0	1.2	0.7	0.6	1.8	2.6	2.9	124.5	-1.42968	4.62E-08
ENSMUSG00000036098	19	Myrf	78.6	64.0	61.6	197.1	128.7	106.2	0.6	0.5	0.4	1.7	1.0	0.9	106.0	-1.07554	3.70E-05
ENSMUSG00000032332	9	Col12a1	34.0	19.4	37.4	245.4	152.1	128.2	0.1	0.1	0.1	1.0	0.6	0.5	102.8	-2.54164	1.04E-16
ENSMUSG00000053897	3	Slc39a8	68.8	65.9	47.1	158.9	133.6	111.0	0.7	0.7	0.5	1.8	1.5	1.2	97.5	-1.13911	3.04E-06
ENSMUSG00000031722	8	Hp	31.3	49.4	29.0	30.2	197.0	243.9	1.1	1.5	0.9	1.1	6.8	8.2	96.8	-2.10181	0.010930966

ENSMUSG00000026051	1	1500015O10Rik	36.6	41.7	58.0	221.3	144.3	75.6	1.8	1.9	2.7	11.9	7.4	3.8	96.2	-1.70320	3.92E-07
ENSMUSG00000041324	13	Inhba	57.2	48.5	48.3	169.0	124.8	98.5	0.4	0.3	0.3	1.3	0.9	0.7	91.0	-1.34467	3.04E-07
ENSMUSG00000064356	MT	mt-Atp8	136.7	114.4	105.1	88.5	53.6	43.0	30.4	23.4	21.3	21.0	12.2	9.6	90.2	0.94904	0.000552997
ENSMUSG00000028459	4	Cd72	74.2	49.4	49.5	135.8	114.1	109.0	1.3	0.8	0.8	2.5	2.0	1.9	88.7	-1.04233	3.13E-05
ENSMUSG00000054196	15	Cthrc1	1.8	8.7	9.7	249.4	139.5	118.6	0.1	0.3	0.3	10.3	5.5	4.6	87.9	-4.70666	3.83E-26
ENSMUSG00000030278	6	Cidec	39.3	31.0	27.8	48.3	171.6	207.6	1.0	0.8	0.7	1.4	4.6	5.5	87.6	-2.11853	9.36E-08
ENSMUSG00000039542	9	Ncam1	60.8	42.7	59.2	154.9	116.1	89.0	0.4	0.3	0.4	1.1	0.8	0.6	87.1	-1.14705	2.50E-05
ENSMUSG00000043110	2	Lrrn4	33.1	8.7	24.1	210.2	143.4	102.4	0.4	0.1	0.3	2.7	1.8	1.3	87.0	-2.78544	2.09E-14
ENSMUSG00000002997	12	Prkar2b	57.2	48.5	51.9	85.5	122.9	152.1	0.8	0.6	0.6	1.2	1.7	2.0	86.3	-1.19346	8.16E-06
ENSMUSG00000105703	14	Gm43305	172.5	96.9	99.0	18.1	46.8	79.4	1.6	0.8	0.8	0.2	0.4	0.7	85.5	1.35233	0.000360744
ENSMUSG00000049436	16	Upk1b	49.1	30.1	20.5	173.0	129.7	89.0	1.2	0.7	0.4	4.4	3.2	2.1	81.9	-1.95266	2.39E-09
ENSMUSG00000027574	2	Nkain4	64.3	29.1	44.7	122.7	116.1	97.6	3.1	1.3	1.9	6.2	5.6	4.7	79.1	-1.27778	9.02E-06
ENSMUSG00000018486	11	Wnt9b	52.7	42.7	45.9	151.9	95.6	67.9	0.5	0.4	0.4	1.6	1.0	0.7	76.1	-1.15515	0.00017862
ENSMUSG00000027513	2	Pck1	3.6	5.8	7.2	26.2	167.7	235.3	0.1	0.1	0.1	0.5	2.8	3.9	74.3	-4.70147	6.06E-07
ENSMUSG00000028871	4	Rspo1	50.0	38.8	27.8	138.8	92.6	89.9	1.2	0.9	0.6	3.7	2.3	2.2	73.0	-1.44690	1.04E-06
ENSMUSG00000061451	19	Tmem151a	48.3	31.0	48.3	110.6	81.9	93.7	0.5	0.3	0.5	1.3	0.9	1.1	69.0	-1.16929	2.94E-05
ENSMUSG00000028364	4	Tnc	45.6	19.4	39.8	138.8	105.3	58.4	0.3	0.1	0.2	0.9	0.7	0.4	67.9	-1.52910	2.32E-05
ENSMUSG00000004473	7	Clec11a	51.8	50.4	25.4	103.6	85.8	86.1	0.8	0.7	0.4	1.7	1.3	1.3	67.2	-1.09251	0.000206191
ENSMUSG00000031112	X	Stk26	46.5	27.1	37.4	112.7	91.7	87.0	0.6	0.3	0.4	1.5	1.2	1.1	67.1	-1.38767	1.31E-06
ENSMUSG00000020911	11	Krt19	44.7	22.3	22.9	134.8	91.7	85.1	1.3	0.6	0.6	4.1	2.7	2.5	66.9	-1.77573	5.81E-08
ENSMUSG00000040703	7	Cyp2s1	48.3	13.6	10.9	122.7	97.5	99.5	0.7	0.2	0.1	1.9	1.4	1.4	65.4	-2.10682	1.54E-07
ENSMUSG00000031351	X	Zfp185	32.2	30.1	32.6	129.7	96.5	67.9	0.4	0.4	0.4	1.8	1.3	0.9	64.8	-1.63337	1.43E-07
ENSMUSG00000030562	7	Nox4	52.7	40.7	26.6	107.6	81.9	71.7	0.6	0.4	0.3	1.3	1.0	0.8	63.5	-1.10555	0.000314145
ENSMUSG00000022878	16	Adipoq	30.4	19.4	16.9	36.2	123.9	147.3	1.1	0.6	0.5	1.4	4.4	5.2	62.3	-2.19416	3.69E-07
ENSMUSG00000109564	9	Muc16	37.5	24.2	16.9	114.7	85.8	76.5	0.1	0.0	0.0	0.2	0.2	0.1	59.3	-1.79289	5.56E-08
ENSMUSG00000030546	7	Plin1	13.4	29.1	25.4	31.2	108.3	138.7	0.2	0.4	0.4	0.5	1.8	2.2	57.7	-2.04459	5.95E-06
ENSMUSG00000030616	7	Sytl2	60.8	35.9	15.7	68.4	87.8	72.7	0.6	0.3	0.1	0.8	0.9	0.8	56.9	-1.00523	0.006591737
ENSMUSG00000063594	7	Gng8	54.5	29.1	19.3	102.6	80.0	43.0	2.7	1.3	0.9	5.4	4.1	2.1	54.8	-1.11447	0.004754896
ENSMUSG00000022097	14	Sftpc	0.0	0.0	0.0	2.0	113.1	184.6	0.0	0.0	0.0	0.1	6.6	10.6	50.0	-9.06380	0.000275199
ENSMUSG00000005124	15	Ccn4	42.9	15.5	24.1	92.5	60.5	57.4	0.4	0.1	0.2	0.9	0.5	0.5	48.8	-1.33780	0.000401722
ENSMUSG00000030159	6	Clec1b	29.5	24.2	29.0	48.3	68.3	74.6	0.3	0.2	0.3	0.5	0.7	0.8	45.6	-1.21112	0.000246663
ENSMUSG00000020607	12	Fam84a	22.3	24.2	21.7	91.5	52.7	58.4	0.2	0.2	0.2	1.1	0.6	0.6	45.1	-1.56506	8.22E-06
ENSMUSG00000024598	18	Fbn2	27.7	18.4	24.1	86.5	47.8	55.5	0.1	0.1	0.1	0.4	0.2	0.2	43.3	-1.43057	7.96E-05
ENSMUSG00000031710	8	Ucp1	0.0	3.9	2.4	1.0	105.3	143.5	0.0	0.1	0.1	0.0	3.0	4.0	42.7	-5.32087	0.004090982
ENSMUSG00000035930	8	Chst4	28.6	19.4	8.5	83.5	55.6	54.5	0.6	0.4	0.2	1.9	1.2	1.2	41.7	-1.74676	1.25E-05
ENSMUSG00000037005	X	Xpnpep2	26.8	20.4	14.5	82.5	56.6	40.2	0.3	0.2	0.2	1.1	0.7	0.5	40.1	-1.52125	9.59E-05
ENSMUSG00000024511	18	Rab27b	31.3	25.2	21.7	68.4	51.7	42.1	0.2	0.2	0.1	0.5	0.3	0.3	40.1	-1.04091	0.003226298
ENSMUSG00000027015	2	Cybrd1	12.5	16.5	15.7	98.6	46.8	49.7	0.1	0.1	0.1	0.9	0.4	0.4	40.0	-2.13040	2.53E-07
ENSMUSG00000007888	8	Crlf1	20.6	12.6	13.3	72.4	56.6	52.6	0.6	0.3	0.3	2.1	1.6	1.5	38.0	-1.95256	2.62E-07
ENSMUSG00000032725	7	Folr2	59.0	49.4	41.1	24.1	24.4	23.9	2.2	1.7	1.4	1.0	0.9	0.9	37.0	1.05182	0.002684252
ENSMUSG00000036596	5	Cpz	28.6	14.5	21.7	65.4	47.8	41.1	0.6	0.3	0.4	1.4	1.0	0.8	36.5	-1.24431	0.001186218
ENSMUSG00000023039	15	Krt7	22.3	8.7	8.5	66.4	51.7	51.7	0.2	0.1	0.1	0.8	0.6	0.6	34.9	-2.07168	1.09E-06
ENSMUSG00000049556	9	Lingo1	29.5	17.4	21.7	62.4	38.0	39.2	0.4	0.2	0.2	0.8	0.5	0.5	34.7	-1.01623	0.008760728
ENSMUSG00000027386	2	Fbln7	24.1	17.4	19.3	73.4	38.0	32.5	0.3	0.2	0.2	1.1	0.5	0.5	34.1	-1.23509	0.00302426
ENSMUSG00000049382	15	Krt8	18.8	11.6	8.5	55.3	60.5	49.7	0.4	0.2	0.2	1.4	1.4	1.2	34.1	-2.06291	3.24E-07
ENSMUSG00000031283	X	Chrdl1	19.7	29.1	14.5	57.3	41.9	37.3	0.2	0.3	0.1	0.7	0.5	0.4	33.3	-1.09807	0.00535013
ENSMUSG00000009376	6	Met	27.7	11.6	18.1	69.4	37.1	31.6	0.2	0.1	0.1	0.5	0.3	0.2	32.6	-1.25536	0.004751066
ENSMUSG00000041482	18	Piezo2	15.2	12.6	22.9	63.4	51.7	29.7	0.1	0.0	0.1	0.3	0.2	0.1	32.6	-1.52764	0.000329577
ENSMUSG00000048776	6	Pthlh	25.0	16.5	20.5	59.3	28.3	45.0	0.7	0.4	0.5	1.8	0.8	1.3	32.4	-1.09170	0.007389306
ENSMUSG00000030054	6	Gp9	21.4	6.8	3.6	20.1	57.5	81.3	1.1	0.3	0.2	1.1	3.0	4.2	31.8	-2.28394	7.33E-05
ENSMUSG00000038179	1	Slamf7	25.0	14.5	13.3	53.3	41.9	33.5	0.3	0.2	0.1	0.6	0.5	0.4	30.3	-1.26629	0.002278083
ENSMUSG00000071078	8	Nr2c2ap	20.6	51.4	50.7	25.1	16.6	15.3	0.6	1.4	1.4	0.8	0.5	0.5	29.9	1.09828	0.014317227
ENSMUSG00000024990	19	Rbp4	20.6	5.8	8.5	59.3	41.0	42.1	0.8	0.2	0.3	2.5	1.6	1.6	29.5	-2.00505	1.99E-05
ENSMUSG00000054342	7	Kcnn4	18.8	19.4	18.1	68.4	22.4	28.7	0.2	0.2	0.2	0.9	0.3	0.4	29.3	-1.08356	0.020695142
ENSMUSG00000012705	8	Retn	29.5	12.6	4.8	30.2	40.0	56.4	1.2	0.5	0.2	1.3	1.6	2.3	28.9	-1.40013	0.005891956
ENSMUSG00000031250	X	Tnmd	3.6	5.8	7.2	84.5	39.0	31.6	0.1	0.2	0.2	3.0	1.3	1.1	28.6	-3.24679	6.62E-09
ENSMUSG00000058914	15	C1qtnf3	9.8	1.0	2.4	80.5	41.0	34.4	0.2	0.0	0.0	1.7	0.8	0.7	28.2	-3.50234	8.60E-09
ENSMUSG00000025105	7	Bnc1	22.3	3.9	7.2	56.3	43.9	34.4	0.2	0.0	0.1	0.6	0.4	0.3	28.0	-1.97839	0.000137752
ENSMUSG00000085527	3	Gm15535	34.8	44.6	33.8	13.1	19.5	21.0	1.0	1.2	0.9	0.4	0.6	0.6	27.8	1.07836	0.008100709
ENSMUSG00000055137	13	Sugct	14.3	17.4	18.1	46.3	31.2	39.2	0.4	0.4	0.4	1.3	0.8	1.0	27.8	-1.23281	0.002578416
ENSMUSG00000026259	1	Ngef	11.6	15.5	14.5	56.3	26.3	40.2	0.2	0.2	0.2	0.9	0.4	0.6	27.4	-1.56515	0.000452188
ENSMUSG00000024371	17	C2	18.8	22.3	12.1	53.3	36.1	18.2	0.3	0.4	0.2	1.0	0.6	0.3	26.8	-1.00354	0.034016886
ENSMUSG00000028874	4	Fgr	18.8	13.6	18.1	28.2	34.1	46.9	0.3	0.2	0.2	0.4	0.5	0.6	26.6	-1.11669	0.008389443
ENSMUSG00000079022	15	Col22a1	7.1	22.3	4.8	55.3	37.1	24.9	0.0	0.1	0.0	0.3	0.2	0.1	25.3	-1.75580	0.001133909
ENSMUSG00000001225	12	Slc26a3	19.7	6.8	13.3	54.3	31.2	25.8	0.3	0.1	0.2	0.8	0.4	0.3	25.2	-1.47829	0.002711752
ENSMUSG00000109108	7	Gm36371	34.0	29.1	41.1	6.0	15.6	23.9	1.4	1.1	1.5	0.3	0.7	1.0	24.9	1.18305	0.013577201
ENSMUSG00000101122	1	Gm17971	17.0	44.6	49.5	14.1	7.8	16.3	1.0	2.3	2.5	0.8	0.4	0.9	24.9	1.53175	0.002530626
ENSMUSG00000024673	19	Ms4a1	12.5	36.8	49.5	5.0	17.6	19.1	0.2	0.5	0.7	0.1	0.3	0.3	23.4	1.22967	0.030684561
ENSMUSG00000038600	6	Atp6v0a4	17.9	22.3	4.8	36.2	38.0	21.0	0.2	0.3	0.1	0.5	0.5	0.3	23.4	-1.05564	0.038146477

ENSMUSG00000020627	12	Klhl29	6.3	7.8	27.8	39.2	29.3	26.8	0.0	0.0	0.2	0.3	0.2	0.2	22.8	-1.23053	0.022367427
ENSMUSG00000026579	1	F5	3.6	2.9	10.9	25.1	50.7	43.0	0.0	0.0	0.1	0.2	0.3	0.3	22.7	-2.84131	6.76E-07
ENSMUSG00000028626	4	Col9a2	11.6	12.6	13.3	44.3	32.2	21.0	0.2	0.2	0.2	0.7	0.5	0.3	22.5	-1.38043	0.003629863
ENSMUSG00000050370	19	Ch25h	21.4	9.7	10.9	32.2	24.4	35.4	0.7	0.3	0.3	1.1	0.8	1.2	22.3	-1.11323	0.019010266
ENSMUSG00000054083	7	Capn12	34.0	25.2	29.0	14.1	11.7	18.2	0.5	0.3	0.4	0.2	0.2	0.3	22.0	1.00439	0.025547015
ENSMUSG00000037625	3	Cldn11	8.9	4.8	9.7	52.3	30.2	25.8	0.2	0.1	0.2	1.4	0.7	0.6	22.0	-2.21701	2.47E-05
ENSMUSG00000023993	17	Treml1	8.0	10.7	8.5	13.1	40.0	49.7	0.3	0.4	0.3	0.5	1.5	1.8	21.7	-1.92140	0.000565207
ENSMUSG00000027070	2	Lrp2	10.7	9.7	10.9	34.2	33.2	30.6	0.0	0.0	0.0	0.1	0.1	0.1	21.5	-1.64828	0.000377208
ENSMUSG00000073802	4	Cdkn2b	14.3	3.9	13.3	45.3	26.3	24.9	0.5	0.1	0.4	1.6	0.9	0.8	21.3	-1.62113	0.002254759
ENSMUSG00000005237	11	Dnah2	27.7	15.5	42.3	17.1	10.7	14.3	0.1	0.0	0.1	0.1	0.0	0.0	21.3	1.00825	0.044213187
ENSMUSG00000089417	3	Gm22009	28.6	33.0	24.1	8.0	23.4	8.6	3.8	4.0	2.9	1.1	3.2	1.1	21.0	1.09974	0.030967648
ENSMUSG00000031494	8	Cd209a	14.3	8.7	9.7	36.2	35.1	21.0	0.4	0.2	0.3	1.2	1.1	0.6	20.8	-1.48675	0.002475452
ENSMUSG00000018919	11	Tm4sf5	8.9	2.9	10.9	46.3	30.2	25.8	0.4	0.1	0.4	2.2	1.4	1.2	20.8	-2.19030	5.55E-05
ENSMUSG00000043243	8	Fam129c	15.2	14.5	10.9	30.2	35.1	19.1	0.2	0.2	0.2	0.5	0.6	0.3	20.8	-1.04464	0.027967878
ENSMUSG00000084314	4	Rps15a-ps3	22.3	33.9	26.6	15.1	12.7	13.4	2.6	3.6	2.8	1.8	1.5	1.5	20.7	1.00891	0.029125314
ENSMUSG00000071068	17	Treml2	15.2	11.6	8.5	21.1	29.3	37.3	0.2	0.1	0.1	0.3	0.3	0.4	20.5	-1.29758	0.00789008
ENSMUSG00000026070	1	Il18r1	12.5	8.7	8.5	39.2	33.2	19.1	0.1	0.1	0.1	0.5	0.4	0.2	20.2	-1.61084	0.001546632
ENSMUSG00000016255	2	Tubb1	12.5	12.6	1.2	26.2	29.3	39.2	0.4	0.4	0.0	0.9	1.0	1.3	20.2	-1.80183	0.001237408
ENSMUSG00000026840	2	Lamc3	11.6	15.5	7.2	28.2	39.0	19.1	0.1	0.1	0.1	0.2	0.3	0.1	20.1	-1.31136	0.009815245
ENSMUSG00000027368	2	Dusp2	13.4	9.7	15.7	26.2	34.1	20.1	0.4	0.3	0.4	0.8	1.0	0.6	19.9	-1.05929	0.028858647
ENSMUSG00000109203	7	Gm32849	19.7	6.8	8.5	33.2	26.3	23.0	0.5	0.2	0.2	0.9	0.7	0.6	19.6	-1.21850	0.01934332
ENSMUSG00000030849	7	Fgfr2	13.4	15.5	9.7	36.2	22.4	20.1	0.1	0.1	0.1	0.4	0.2	0.2	19.6	-1.01616	0.039419911
ENSMUSG00000034028	18	Cd226	15.2	3.9	2.4	15.1	46.8	33.5	0.2	0.0	0.0	0.2	0.5	0.3	19.5	-2.10718	0.00097287
ENSMUSG00000097252	3	Gm6634	11.6	5.8	13.3	23.1	31.2	29.7	0.3	0.1	0.3	0.6	0.8	0.7	19.1	-1.46427	0.003798302
ENSMUSG00000029304	5	Spp1	4.5	7.8	19.3	46.3	20.5	16.3	0.1	0.2	0.5	1.5	0.6	0.5	19.1	-1.43200	0.017891436
ENSMUSG00000036298	15	Slc2a13	12.5	7.8	8.5	29.2	19.5	34.4	0.1	0.0	0.1	0.2	0.1	0.2	18.6	-1.52220	0.00315856
ENSMUSG00000062760	15	Shisal1	12.5	12.6	4.8	30.2	33.2	18.2	0.1	0.1	0.0	0.2	0.3	0.1	18.6	-1.41655	0.007605029
ENSMUSG00000073016	X	Uprt	15.2	5.8	7.2	9.1	27.3	45.9	0.3	0.1	0.1	0.2	0.5	0.9	18.4	-1.52837	0.014491087
ENSMUSG00000047844	X	Bex4	13.4	8.7	10.9	29.2	17.6	30.6	0.7	0.4	0.5	1.7	1.0	1.7	18.4	-1.22460	0.015936872
ENSMUSG00000106917	5	Gm7832	17.9	34.9	20.5	13.1	13.7	8.6	0.9	1.7	1.0	0.7	0.7	0.4	18.1	1.05589	0.04109842
ENSMUSG00000036856	4	Wnt4	12.5	11.6	4.8	34.2	23.4	21.0	0.1	0.1	0.0	0.4	0.3	0.2	17.9	-1.41145	0.007881533
ENSMUSG00000027966	3	Col11a1	3.6	0.0	2.4	52.3	27.3	21.0	0.0	0.0	0.0	0.3	0.2	0.1	17.8	-4.06122	2.02E-07
ENSMUSG00000035403	2	Crb2	9.8	11.6	0.0	32.2	21.5	30.6	0.1	0.1	0.0	0.2	0.2	0.2	17.6	-1.92246	0.00178097
ENSMUSG00000053930	11	Shisa6	22.3	23.3	35.0	8.0	8.8	7.7	0.1	0.1	0.2	0.1	0.1	0.0	17.5	1.71045	0.001147431
ENSMUSG00000034687	5	Fras1	17.0	2.9	3.6	24.1	28.3	28.7	0.0	0.0	0.0	0.1	0.1	0.1	17.4	-1.74677	0.004257377
ENSMUSG00000003411	4	Rab3b	14.3	6.8	6.0	22.1	23.4	31.6	0.2	0.1	0.1	0.3	0.3	0.4	17.4	-1.48261	0.005978019
ENSMUSG00000033491	9	Prss35	13.4	5.8	13.3	28.2	25.4	16.3	0.2	0.1	0.2	0.4	0.3	0.2	17.0	-1.10700	0.039537374
ENSMUSG00000053626	8	Tll1	13.4	2.9	7.2	41.2	19.5	17.2	0.1	0.0	0.1	0.4	0.2	0.2	16.9	-1.71052	0.005490743
ENSMUSG00000022012	14	Enox1	6.3	13.6	9.7	24.1	20.5	26.8	0.1	0.2	0.1	0.4	0.3	0.4	16.8	-1.28168	0.015283103
ENSMUSG00000104346	18	Pcdhga3	7.1	14.5	6.0	26.2	17.6	27.7	0.1	0.1	0.1	0.3	0.2	0.3	16.5	-1.35352	0.013959764
ENSMUSG00000024987	19	Cyp26a1	8.0	1.9	2.4	45.3	22.4	17.2	0.2	0.0	0.1	1.2	0.6	0.4	16.2	-2.73053	5.75E-05
ENSMUSG00000032952	3	Ap4b1	18.8	23.3	33.8	6.0	5.9	9.6	0.3	0.3	0.5	0.1	0.1	0.2	16.2	1.80855	0.00127379
ENSMUSG00000026205	1	Slc23a3	9.8	2.9	16.9	30.2	20.5	16.3	0.2	0.1	0.3	0.7	0.5	0.4	16.1	-1.20107	0.045802366
ENSMUSG00000061048	8	Cdh3	7.1	1.0	3.6	39.2	19.5	25.8	0.1	0.0	0.0	0.4	0.2	0.3	16.0	-2.82350	2.19E-05
ENSMUSG00000018849	11	Wwc1	8.0	5.8	7.2	37.2	8.8	27.7	0.1	0.1	0.1	0.4	0.1	0.3	15.8	-1.80274	0.004148748
ENSMUSG00000074220	7	Zfp382	15.2	6.8	4.8	18.1	24.4	24.9	0.3	0.1	0.1	0.4	0.5	0.5	15.7	-1.29889	0.022546027
ENSMUSG00000028654	4	Mycl	16.1	5.8	7.2	21.1	15.6	27.7	0.2	0.1	0.1	0.3	0.2	0.4	15.6	-1.12581	0.04977948
ENSMUSG00000057346	15	Apol9a	12.5	6.8	8.5	23.1	19.5	23.0	0.1	0.1	0.1	0.3	0.2	0.2	15.6	-1.23089	0.022322684
ENSMUSG00000050359	3	Sprr1a	0.9	2.9	2.4	36.2	30.2	20.1	0.1	0.2	0.1	2.2	1.7	1.1	15.5	-3.82877	5.26E-07
ENSMUSG00000031861	8	Lpar2	11.6	5.8	10.9	29.2	13.7	20.1	0.1	0.0	0.1	0.2	0.1	0.2	15.2	-1.15407	0.042870953
ENSMUSG00000019936	10	Epyc	11.6	1.0	4.8	27.2	31.2	15.3	0.2	0.0	0.1	0.5	0.6	0.3	15.2	-2.05509	0.002115064
ENSMUSG00000023232	4	Serinc2	2.7	9.7	4.8	19.1	23.4	30.6	0.1	0.2	0.1	0.5	0.6	0.7	15.1	-2.09098	0.000659576
ENSMUSG00000097793	13	Gm17259	15.2	28.1	20.5	10.1	14.6	1.0	0.1	0.3	0.2	0.1	0.1	0.0	14.9	1.31807	0.045803611
ENSMUSG00000020399	11	Havcr2	4.5	8.7	7.2	28.2	24.4	16.3	0.1	0.1	0.1	0.5	0.4	0.3	14.9	-1.76014	0.002556339
ENSMUSG00000015709	7	Arnt2	8.9	8.7	7.2	31.2	20.5	12.4	0.1	0.1	0.0	0.2	0.2	0.1	14.8	-1.35430	0.019764323
ENSMUSG00000045246	8	Kcng4	25.9	13.6	22.9	7.0	12.7	5.7	0.3	0.1	0.2	0.1	0.2	0.1	14.6	1.29373	0.026054499
ENSMUSG00000040133	2	Gpr176	0.9	2.9	6.0	39.2	13.7	24.9	0.0	0.0	0.1	0.5	0.2	0.3	14.6	-3.05408	3.09E-05
ENSMUSG00000029168	5	Dpysl5	12.5	2.9	4.8	40.2	15.6	10.5	0.1	0.0	0.0	0.4	0.1	0.1	14.4	-1.68688	0.015411155
ENSMUSG00000051048	7	P4ha3	4.5	4.8	6.0	36.2	18.5	16.3	0.1	0.1	0.1	0.7	0.4	0.3	14.4	-2.22028	0.000454561
ENSMUSG00000030134	6	Rasgef1a	11.6	8.7	6.0	21.1	20.5	17.2	0.2	0.1	0.1	0.3	0.3	0.2	14.2	-1.13631	0.042107253
ENSMUSG00000073414	17	Mpig6b	7.1	2.9	13.3	17.1	20.5	23.9	0.1	0.1	0.2	0.4	0.4	0.5	14.1	-1.43273	0.018630076
ENSMUSG00000095463	14	Entpd4	11.6	7.8	4.8	21.1	14.6	23.0	0.1	0.1	0.1	0.3	0.2	0.3	13.8	-1.25280	0.032195638
ENSMUSG00000059864	11	Olfr1393	16.1	14.5	27.8	7.0	5.9	8.6	0.2	0.2	0.4	0.1	0.1	0.1	13.3	1.42754	0.0171903
ENSMUSG00000030905	7	Crym	10.7	4.8	4.8	13.1	17.6	27.7	0.2	0.1	0.1	0.2	0.3	0.5	13.1	-1.49536	0.017357539
ENSMUSG00000048521	9	Cxcr6	12.5	5.8	1.2	22.1	20.5	16.3	0.3	0.1	0.0	0.6	0.5	0.4	13.1	-1.54103	0.019307359
ENSMUSG00000027887	3	Sypl2	9.8	1.9	10.9	13.1	22.4	20.1	0.1	0.0	0.1	0.2	0.3	0.2	13.0	-1.31163	0.041863609
ENSMUSG00000030117	6	Gdf3	5.4	1.9	4.8	28.2	25.4	12.4	0.1	0.0	0.1	0.6	0.6	0.3	13.0	-2.44559	0.0003583
ENSMUSG00000085433	5	Gm16001	5.4	5.8	10.9	21.1	20.5	14.3	0.2	0.2	0.5	1.0	1.0	0.7	13.0	-1.37126	0.022754914
ENSMUSG00000073400	17	Trim10	42.9	7.8	7.2	3.0	7.8	8.6	0.9	0.1	0.1	0.1	0.2	0.2	12.9	1.58735	0.038733941

ENSMUSG00000046607	5	Hrk	21.4	17.4	15.7	7.0	6.8	8.6	0.2	0.1	0.1	0.1	0.1	0.1	12.8	1.28496	0.027673922
ENSMUSG00000109115	7	Gm44669	2.7	12.6	7.2	16.1	15.6	22.0	0.1	0.3	0.2	0.4	0.4	0.5	12.7	-1.26250	0.048418382
ENSMUSG00000097993	1	Ptprv	4.5	5.8	8.5	32.2	14.6	10.5	0.0	0.0	0.1	0.3	0.1	0.1	12.7	-1.63233	0.014056861
ENSMUSG00000056370	6	Sftpb	8.0	1.9	6.0	6.0	22.4	30.6	0.2	0.1	0.2	0.2	0.7	0.9	12.5	-1.88298	0.010342911
ENSMUSG00000115867	15	Gm17753	3.6	9.7	7.2	20.1	21.5	12.4	0.1	0.3	0.2	0.6	0.6	0.4	12.4	-1.40675	0.025526232
ENSMUSG00000023013	15	Aqp2	7.1	8.7	4.8	28.2	12.7	11.5	0.2	0.3	0.1	1.0	0.4	0.4	12.2	-1.32088	0.041226863
ENSMUSG00000032500	9	Dclk3	8.9	4.8	4.8	28.2	11.7	14.3	0.1	0.1	0.1	0.5	0.2	0.2	12.1	-1.52230	0.020152557
ENSMUSG00000006457	19	Actn3	5.4	10.7	1.2	14.1	19.5	21.0	0.1	0.2	0.0	0.2	0.3	0.3	12.0	-1.63005	0.016057136
ENSMUSG00000041737	9	Tmem45b	2.7	1.9	3.6	3.0	24.4	35.4	0.0	0.0	0.1	0.1	0.4	0.6	11.8	-2.95021	0.00068102
ENSMUSG00000058794	15	Nfe2	8.9	5.8	3.6	11.1	14.6	26.8	0.2	0.1	0.1	0.3	0.4	0.6	11.8	-1.48956	0.02464802
ENSMUSG00000104737	3	Gm42937	14.3	21.3	18.1	6.0	6.8	3.8	0.9	1.2	1.0	0.4	0.4	0.2	11.7	1.68699	0.007908808
ENSMUSG00000078902	2	Gm14443	15.2	12.6	22.9	6.0	6.8	5.7	0.2	0.1	0.2	0.1	0.1	0.1	11.6	1.43685	0.022864326
ENSMUSG00000031489	8	Adrb3	8.9	1.0	0.0	5.0	14.6	39.2	0.1	0.0	0.0	0.1	0.2	0.6	11.5	-2.50733	0.007146147
ENSMUSG00000025582	11	Nptx1	9.8	4.8	4.8	20.1	20.5	8.6	0.1	0.0	0.0	0.2	0.2	0.1	11.5	-1.31349	0.048184787
ENSMUSG00000052613	10	Pcdh15	7.1	2.9	3.6	24.1	19.5	9.6	0.0	0.0	0.0	0.1	0.1	0.0	11.1	-1.93610	0.005984138
ENSMUSG00000017723	2	Wfdc2	2.7	0.0	1.2	14.1	24.4	23.0	0.1	0.0	0.1	0.8	1.3	1.2	10.9	-3.94385	2.24E-05
ENSMUSG00000106874	6	Gm20186	6.3	4.8	7.2	15.1	20.5	10.5	0.4	0.3	0.4	1.0	1.3	0.6	10.7	-1.33695	0.041420583
ENSMUSG00000051076	3	Vtcn1	8.0	1.9	3.6	28.2	10.7	10.5	0.1	0.0	0.1	0.5	0.2	0.2	10.5	-1.83561	0.015079993
ENSMUSG00000064246	1	Chil1	5.4	1.9	3.6	14.1	11.7	25.8	0.1	0.0	0.1	0.4	0.3	0.6	10.4	-2.23140	0.002562538
ENSMUSG00000047953	16	Gp5	6.3	1.9	4.8	8.0	15.6	25.8	0.1	0.0	0.1	0.2	0.3	0.5	10.4	-1.92558	0.009671566
ENSMUSG00000102758	3	Naaladl2	6.3	4.8	6.0	20.1	14.6	10.5	0.0	0.0	0.0	0.1	0.1	0.1	10.4	-1.40036	0.035375776
ENSMUSG00000052026	18	Slc6a7	6.3	1.0	8.5	14.1	25.4	6.7	0.1	0.0	0.1	0.2	0.3	0.1	10.3	-1.57969	0.042268408
ENSMUSG00000006389	4	Mpl	5.4	3.9	2.4	12.1	17.6	16.3	0.1	0.1	0.0	0.2	0.3	0.3	9.6	-1.94861	0.006729889
ENSMUSG00000063354	15	Slc39a4	1.8	3.9	3.6	16.1	8.8	22.0	0.0	0.1	0.1	0.3	0.1	0.4	9.4	-2.35603	0.002680717
ENSMUSG00000036136	12	Fam110c	8.0	2.9	1.2	22.1	13.7	7.7	0.1	0.0	0.0	0.3	0.2	0.1	9.3	-1.78526	0.024624812
ENSMUSG00000020793	11	Galr2	3.6	3.9	1.2	17.1	18.5	10.5	0.1	0.1	0.0	0.4	0.4	0.2	9.1	-2.37232	0.002381897
ENSMUSG00000017002	2	Slpi	3.6	1.0	3.6	2.0	13.7	30.6	0.2	0.0	0.2	0.1	0.7	1.6	9.1	-2.51614	0.007842077
ENSMUSG00000020672	12	Sntg2	3.6	1.0	8.5	24.1	6.8	9.6	0.1	0.0	0.1	0.5	0.1	0.2	8.9	-1.68380	0.043642325
ENSMUSG00000063109	11	Dgkeos	12.5	16.5	9.7	6.0	3.9	4.8	0.3	0.4	0.2	0.2	0.1	0.1	8.9	1.40125	0.04845196
ENSMUSG00000111088	9	9430081H08Rik	16.1	14.5	9.7	4.0	3.9	4.8	0.9	0.7	0.5	0.2	0.2	0.3	8.8	1.67310	0.020737637
ENSMUSG00000024972	19	Lgals12	1.8	1.0	0.0	8.0	23.4	18.2	0.0	0.0	0.0	0.1	0.4	0.3	8.7	-4.06647	0.000154807
ENSMUSG00000085139	X	A730046J19Rik	4.5	1.0	3.6	21.1	12.7	7.7	0.0	0.0	0.0	0.2	0.1	0.1	8.4	-2.19274	0.008251145
ENSMUSG00000031284	X	Pak3	6.3	5.8	0.0	10.1	12.7	15.3	0.0	0.0	0.0	0.1	0.1	0.1	8.4	-1.60145	0.046019014
ENSMUSG00000004864	17	Mapk13	1.8	5.8	2.4	20.1	9.8	9.6	0.0	0.1	0.0	0.3	0.2	0.1	8.2	-1.96908	0.01537935
ENSMUSG00000039748	1	Exo1	20.6	8.7	10.9	4.0	2.0	2.9	0.1	0.0	0.1	0.0	0.0	0.0	8.2	2.19427	0.007112502
ENSMUSG00000078810	7	Gp6	4.5	3.9	2.4	11.1	6.8	20.1	0.0	0.0	0.0	0.1	0.1	0.2	8.1	-1.79935	0.024445497
ENSMUSG00000059900	6	Tmem40	1.8	4.8	1.2	4.0	10.7	24.9	0.1	0.1	0.0	0.1	0.3	0.7	7.9	-2.31817	0.012047952
ENSMUSG00000021675	13	F2rl2	2.7	1.0	3.6	9.1	11.7	19.1	0.0	0.0	0.1	0.2	0.2	0.3	7.9	-2.48580	0.003905238
ENSMUSG00000002588	6	Pon1	0.0	4.8	2.4	2.0	14.6	22.0	0.0	0.1	0.1	0.1	0.4	0.6	7.7	-2.43204	0.017261236
ENSMUSG00000052974	7	Cyp2f2	4.5	1.0	1.2	3.0	10.7	24.9	0.1	0.0	0.0	0.1	0.3	0.6	7.5	-2.49840	0.011158311
ENSMUSG00000097888	18	Gm26682	5.4	15.5	15.7	5.0	1.0	1.9	0.1	0.4	0.4	0.1	0.0	0.0	7.4	2.20552	0.013349286
ENSMUSG00000113737	13	BB123696	6.3	15.5	13.3	1.0	3.9	3.8	0.1	0.3	0.3	0.0	0.1	0.1	7.3	1.99122	0.019868907
ENSMUSG00000037962	5	Rflna	1.8	1.9	4.8	13.1	17.6	3.8	0.0	0.0	0.1	0.4	0.5	0.1	7.2	-2.05302	0.023164263
ENSMUSG00000069273	13	Hist1h3e	3.6	1.9	2.4	8.0	9.8	17.2	0.2	0.1	0.1	0.5	0.5	0.9	7.2	-2.13223	0.01285632
ENSMUSG00000095788	3	Sirpb1a	1.8	0.0	3.6	17.1	9.8	7.7	0.0	0.0	0.1	0.4	0.2	0.2	6.7	-2.73731	0.0056896
ENSMUSG00000021208	12	Ifi27l2b	4.5	2.9	1.2	9.1	13.7	8.6	0.2	0.1	0.0	0.4	0.6	0.4	6.6	-1.82462	0.033716714
ENSMUSG00000070473	5	Cldn3	0.0	5.8	1.2	19.1	8.8	3.8	0.0	0.2	0.0	0.7	0.3	0.1	6.5	-2.16642	0.037958176
ENSMUSG00000029005	4	Draxin	5.4	0.0	1.2	8.0	10.7	13.4	0.0	0.0	0.0	0.1	0.1	0.1	6.5	-2.23968	0.020938088
ENSMUSG00000086283	11	2810433D01Rik	2.7	3.9	1.2	13.1	5.9	11.5	0.1	0.2	0.0	0.6	0.3	0.5	6.4	-1.93823	0.03027977
ENSMUSG00000018924	11	Alox15	1.8	0.0	0.0	5.0	14.6	16.3	0.0	0.0	0.0	0.1	0.3	0.3	6.3	-4.19344	0.001326771
ENSMUSG00000028716	4	Pdzk1ip1	1.8	2.9	3.6	8.0	8.8	11.5	0.1	0.1	0.1	0.3	0.4	0.5	6.1	-1.79280	0.04132083
ENSMUSG00000098021	15	Gm9522	10.7	12.6	13.3	0.0	0.0	0.0	0.5	0.6	0.6	0.0	0.0	0.0	6.1	6.07975	1.41E-05
ENSMUSG00000027445	2	Cst9	2.7	0.0	1.2	16.1	11.7	4.8	0.2	0.0	0.1	1.1	0.8	0.3	6.1	-3.03285	0.005219946
ENSMUSG00000031725	8	Ces1f	2.7	1.9	0.0	7.0	10.7	13.4	0.1	0.0	0.0	0.2	0.3	0.3	6.0	-2.67438	0.007886453
ENSMUSG00000037579	15	Kcnh3	1.8	3.9	1.2	15.1	2.9	10.5	0.0	0.0	0.0	0.2	0.0	0.1	5.9	-2.03176	0.038990845
ENSMUSG00000112857	12	Gm35041	7.1	10.7	9.7	1.0	2.9	2.9	0.5	0.7	0.7	0.1	0.2	0.2	5.7	2.00472	0.030726444
ENSMUSG00000030666	7	Calcb	2.7	1.0	2.4	15.1	6.8	5.7	0.1	0.0	0.1	0.8	0.3	0.3	5.6	-2.19032	0.02615099
ENSMUSG00000001670	8	Tat	2.7	0.0	2.4	10.1	5.9	12.4	0.1	0.0	0.0	0.2	0.1	0.2	5.6	-2.48863	0.015903292
ENSMUSG00000033508	6	Asprv1	3.6	1.9	1.2	5.0	12.7	8.6	0.1	0.1	0.0	0.2	0.4	0.3	5.5	-1.93304	0.044156431
ENSMUSG00000078302	13	Foxd1	0.9	3.9	0.0	8.0	4.9	14.3	0.0	0.0	0.0	0.1	0.0	0.1	5.3	-2.46813	0.022592326
ENSMUSG00000026981	2	Il1rn	1.8	1.9	2.4	10.1	9.8	4.8	0.0	0.0	0.0	0.2	0.2	0.1	5.1	-2.01353	0.040943873
ENSMUSG00000057092	7	Fxyd3	0.9	1.9	2.4	9.1	8.8	7.7	0.0	0.0	0.0	0.2	0.1	0.1	5.1	-2.31590	0.021762177
ENSMUSG00000092201	2	A530058N18Rik	4.5	11.6	9.7	2.0	0.0	2.9	0.1	0.4	0.3	0.1	0.0	0.1	5.1	2.39132	0.025412572
ENSMUSG00000026241	1	Nppc	4.5	0.0	0.0	13.1	6.8	5.7	0.2	0.0	0.0	0.6	0.3	0.3	5.0	-2.42584	0.035121936
ENSMUSG00000022066	14	Entpd4b	0.9	0.0	0.0	21.1	3.9	3.8	0.0	0.0	0.0	0.3	0.1	0.0	5.0	-4.72153	0.001964719
ENSMUSG00000034730	15	Adgrb1	13.4	5.8	4.8	2.0	1.0	1.0	0.1	0.0	0.0	0.0	0.0	0.0	4.7	2.62930	0.018927054
ENSMUSG00000025014	19	Dntt	1.8	0.0	0.0	0.0	11.7	14.3	0.0	0.0	0.0	0.0	0.3	0.3	4.6	-3.75453	0.014859387
ENSMUSG00000091476	1	Catspere2	0.9	2.9	1.2	9.1	6.8	6.7	0.0	0.0	0.0	0.1	0.1	0.1	4.6	-2.16509	0.039916992
ENSMUSG00000074715	13	Ccl28	8.0	6.8	7.2	0.0	2.0	2.9	0.1	0.1	0.1	0.0	0.0	0.0	4.5	2.18391	0.044239993

ENSMUSG00000055368	8	Slc6a2	16.1	6.8	0.0	2.0	1.0	1.0	0.1	0.0	0.0	0.0	0.0	0.0	4.5	2.56384	0.044692895
ENSMUSG00000044217	15	Aqp5	0.9	0.0	1.2	3.0	2.0	19.1	0.0	0.0	0.0	0.1	0.1	0.5	4.4	-3.56636	0.014385965
ENSMUSG00000029377	5	Ereg	0.0	2.9	0.0	15.1	3.9	3.8	0.0	0.0	0.0	0.2	0.0	0.0	4.3	-2.93092	0.027009888
ENSMUSG00000097125	17	Gm26885	0.9	1.0	1.2	9.1	9.8	3.8	0.0	0.0	0.0	0.1	0.1	0.1	4.3	-2.89623	0.016378662
ENSMUSG00000039058	3	Ak5	1.8	1.0	1.2	10.1	5.9	5.7	0.0	0.0	0.0	0.1	0.1	0.1	4.3	-2.43329	0.030286805
ENSMUSG00000060962	7	Dmkn	0.0	2.9	0.0	7.0	6.8	8.6	0.0	0.1	0.0	0.2	0.2	0.2	4.2	-2.91051	0.020608752
ENSMUSG00000056423	16	Uts2b	1.8	0.0	0.0	3.0	7.8	11.5	0.1	0.0	0.0	0.2	0.4	0.6	4.0	-3.51832	0.013746417
ENSMUSG00000034156	11	Tspoap1	0.9	1.9	1.2	10.1	4.9	4.8	0.0	0.0	0.0	0.1	0.0	0.0	4.0	-2.28631	0.047705464
ENSMUSG00000057729	10	Prtn3	0.9	0.0	2.4	2.0	6.8	11.5	0.0	0.0	0.1	0.1	0.3	0.5	3.9	-2.70247	0.036829837
ENSMUSG00000049008	8	BB014433	0.9	1.0	1.2	6.0	3.9	9.6	0.0	0.0	0.0	0.2	0.1	0.2	3.8	-2.68380	0.030488499
ENSMUSG00000079186	14	Gzmc	1.8	0.0	0.0	11.1	2.9	6.7	0.1	0.0	0.0	0.6	0.2	0.3	3.7	-3.40799	0.01873331
ENSMUSG00000097674	7	Gm26610	2.7	7.8	8.5	1.0	1.0	1.0	0.1	0.3	0.3	0.0	0.0	0.0	3.6	2.67200	0.034564151
ENSMUSG00000117613	18	Gm2629	0.9	0.0	0.0	12.1	5.9	2.9	0.0	0.0	0.0	0.4	0.2	0.1	3.6	-4.24940	0.006431775
ENSMUSG00000030162	6	Olr1	1.8	0.0	0.0	11.1	2.9	5.7	0.0	0.0	0.0	0.1	0.0	0.1	3.6	-3.34079	0.022639495
ENSMUSG00000040434	2	Large2	1.8	1.0	0.0	9.1	4.9	4.8	0.0	0.0	0.0	0.1	0.0	0.0	3.6	-2.67364	0.037084399
ENSMUSG00000010154	8	Spire2	2.7	0.0	0.0	7.0	7.8	3.8	0.1	0.0	0.0	0.1	0.2	0.1	3.6	-2.69173	0.04410804
ENSMUSG00000052353	7	Cemip	0.0	0.0	0.0	12.1	4.9	3.8	0.0	0.0	0.0	0.1	0.0	0.0	3.5	-5.21028	0.001009098
ENSMUSG00000091345	9	Col6a5	0.9	0.0	1.2	6.0	8.8	3.8	0.0	0.0	0.0	0.0	0.0	0.0	3.5	-3.19394	0.02359677
ENSMUSG00000040138	X	Ndp	0.9	0.0	0.0	10.1	2.0	7.7	0.0	0.0	0.0	0.3	0.0	0.2	3.4	-4.17053	0.008426711
ENSMUSG00000105565	5	Gm43566	0.9	0.0	1.2	8.0	3.9	5.7	0.1	0.0	0.1	0.5	0.2	0.3	3.3	-3.11744	0.028356854
ENSMUSG00000001773	7	Folh1	0.0	0.0	1.2	5.0	6.8	6.7	0.0	0.0	0.0	0.1	0.1	0.1	3.3	-4.08854	0.008222102
ENSMUSG00000026343	1	Gpr39	0.9	0.0	0.0	9.1	5.9	3.8	0.0	0.0	0.0	0.2	0.1	0.1	3.3	-4.09971	0.008694933
ENSMUSG00000028262	3	Clca3a2	1.8	0.0	0.0	9.1	5.9	2.9	0.0	0.0	0.0	0.1	0.1	0.0	3.3	-3.19171	0.031897823
ENSMUSG00000099889	7	Mrgprb11-ps	0.0	1.0	1.2	11.1	3.9	1.9	0.0	0.0	0.1	0.5	0.2	0.1	3.2	-3.02424	0.041675292
ENSMUSG00000092286	12	Dtnbos	0.0	1.9	0.0	5.0	9.8	1.9	0.0	0.0	0.0	0.1	0.3	0.1	3.1	-3.06493	0.044531869
ENSMUSG00000045502	5	Hcar2	0.9	0.0	1.2	1.0	5.9	9.6	0.0	0.0	0.0	0.0	0.1	0.2	3.1	-3.01191	0.046141513
ENSMUSG00000086607	18	4930511M06Rik	4.5	5.8	7.2	0.0	1.0	0.0	0.1	0.1	0.1	0.0	0.0	0.0	3.1	4.05113	0.009976657
ENSMUSG00000026387	1	Sctr	0.0	0.0	0.0	2.0	3.9	12.4	0.0	0.0	0.0	0.0	0.1	0.3	3.1	-5.03657	0.002526178
ENSMUSG00000040264	3	Gbp2b	8.9	1.0	6.0	0.0	1.0	1.0	0.1	0.0	0.1	0.0	0.0	0.0	3.0	3.03432	0.046665793
ENSMUSG00000082738	14	Gm15422	0.0	1.0	1.2	4.0	6.8	4.8	0.0	0.1	0.1	0.4	0.6	0.4	3.0	-2.92092	0.043907165
ENSMUSG00000038415	13	Foxq1	1.8	0.0	0.0	8.0	2.0	5.7	0.0	0.0	0.0	0.1	0.0	0.1	2.9	-3.02197	0.049925324
ENSMUSG00000041552	X	Ptchd1	0.9	0.0	0.0	8.0	4.9	2.9	0.0	0.0	0.0	0.1	0.1	0.0	2.8	-3.85324	0.017585134
ENSMUSG00000114654	13	Gm47918	4.5	5.8	4.8	0.0	0.0	1.0	0.3	0.4	0.3	0.0	0.0	0.1	2.7	3.84441	0.017500558
ENSMUSG00000021090	12	Lrrc9	0.0	1.0	0.0	8.0	3.9	2.9	0.0	0.0	0.0	0.1	0.0	0.0	2.6	-3.76105	0.022332283
ENSMUSG00000053030	5	Spink2	0.0	0.0	0.0	9.1	2.9	2.9	0.0	0.0	0.0	0.7	0.2	0.2	2.5	-4.72568	0.005905326
ENSMUSG00000108660	6	Gm21284	0.0	1.0	0.0	4.0	5.9	3.8	0.0	0.0	0.0	0.1	0.1	0.0	2.4	-3.65062	0.027242661
ENSMUSG00000062252	17	Lhfpl5	4.5	6.8	1.2	1.0	0.0	0.0	0.1	0.2	0.0	0.0	0.0	0.0	2.2	3.58090	0.039149928
ENSMUSG00000055333	11	Fat2	3.6	3.9	4.8	0.0	0.0	1.0	0.0	0.0	0.0	0.0	0.0	0.0	2.2	3.53986	0.036724882
ENSMUSG00000061911	12	Myt1l	4.5	2.9	4.8	1.0	0.0	0.0	0.0	0.0	0.0	0.0	0.0	0.0	2.2	3.53253	0.037684394
ENSMUSG00000085207	11	Gm11767	8.0	2.9	1.2	0.0	1.0	0.0	0.7	0.2	0.1	0.0	0.1	0.0	2.2	3.55075	0.044061999
ENSMUSG00000117522	17	Gm50041	8.0	2.9	1.2	0.0	0.0	1.0	1.7	0.6	0.2	0.0	0.0	0.2	2.2	3.55073	0.044125316
ENSMUSG00000056716	10	Gm5420	4.5	3.9	3.6	1.0	0.0	0.0	0.1	0.1	0.1	0.0	0.0	0.0	2.2	3.51064	0.039216392
ENSMUSG00000072721	6	Klra14-ps	0.0	0.0	1.2	3.0	4.9	3.8	0.0	0.0	0.0	0.1	0.2	0.2	2.2	-3.42590	0.044664185
ENSMUSG00000097385	6	Gm26814	1.8	3.9	7.2	0.0	0.0	0.0	0.0	0.0	0.1	0.0	0.0	0.0	2.2	4.56205	0.010326305
ENSMUSG00000010601	15	Apol7a	0.0	0.0	0.0	7.0	4.9	1.0	0.0	0.0	0.0	0.1	0.1	0.0	2.1	-4.51988	0.012514302
ENSMUSG00000092130	12	D030025P21Rik	0.0	0.0	0.0	3.0	3.9	5.7	0.0	0.0	0.0	0.0	0.0	0.1	2.1	-4.49942	0.010310311
ENSMUSG00000059854	8	Hydin	0.9	0.0	0.0	2.0	5.9	3.8	0.0	0.0	0.0	0.0	0.0	0.0	2.1	-3.42285	0.048385402
ENSMUSG00000020890	11	Gucy2e	3.6	1.9	6.0	0.0	0.0	1.0	0.0	0.0	0.0	0.0	0.0	0.0	2.1	3.44324	0.04814354
ENSMUSG00000105547	16	Iglc3	5.4	4.8	1.2	1.0	0.0	0.0	0.5	0.4	0.1	0.1	0.0	0.0	2.1	3.45672	0.049848342
ENSMUSG00000110521	9	Olfr856-ps1	0.0	0.0	0.0	7.0	2.0	2.9	0.0	0.0	0.0	0.4	0.1	0.1	2.0	-4.40194	0.015411962
ENSMUSG00000051029	13	Serpinb1b	0.0	0.0	0.0	5.0	2.9	3.8	0.0	0.0	0.0	0.1	0.1	0.1	2.0	-4.39377	0.013871091
ENSMUSG00000107531	8	E330037G11Rik	0.0	0.0	0.0	5.0	1.0	5.7	0.0	0.0	0.0	0.2	0.0	0.2	2.0	-4.38996	0.017319576
ENSMUSG00000085569	4	Gm12602	0.0	0.0	0.0	2.0	7.8	1.9	0.0	0.0	0.0	0.1	0.5	0.1	2.0	-4.38828	0.017332584
ENSMUSG00000051596	5	Otop1	0.0	0.0	0.0	2.0	5.9	3.8	0.0	0.0	0.0	0.0	0.1	0.1	1.9	-4.38447	0.015300703
ENSMUSG00000058022	13	Adtrp	0.0	0.0	0.0	0.0	7.8	3.8	0.0	0.0	0.0	0.0	0.1	0.1	1.9	-4.37758	0.028175794
ENSMUSG00000062345	1	Serpinb2	0.0	0.0	0.0	0.0	5.9	5.7	0.0	0.0	0.0	0.0	0.1	0.1	1.9	-4.37356	0.027530286
ENSMUSG00000045991	18	Onecut2	0.9	1.9	8.5	0.0	0.0	0.0	0.0	0.0	0.0	0.0	0.0	0.0	1.9	4.35871	0.022709731
ENSMUSG00000116555	16	Gm38477	0.0	0.0	0.0	6.0	2.9	1.9	0.0	0.0	0.0	0.1	0.0	0.0	1.8	-4.27651	0.020909796
ENSMUSG00000097282	17	5031415H12Rik	0.0	0.0	0.0	3.0	0.0	7.7	0.0	0.0	0.0	0.1	0.0	0.1	1.8	-4.25325	0.038090942
ENSMUSG00000041301	6	Cftr	0.0	0.0	0.0	1.0	2.9	6.7	0.0	0.0	0.0	0.0	0.0	0.0	1.8	-4.24885	0.025561796
ENSMUSG00000043727	2	F830045P16Rik	0.0	0.0	0.0	4.0	2.9	2.9	0.0	0.0	0.0	0.1	0.1	0.1	1.6	-4.13087	0.027931279
ENSMUSG00000021198	12	Unc79	0.0	0.0	0.0	2.0	5.9	1.9	0.0	0.0	0.0	0.0	0.0	0.0	1.6	-4.12574	0.031447345
ENSMUSG00000051920	15	Rspo2	0.0	0.0	0.0	3.0	2.9	3.8	0.0	0.0	0.0	0.0	0.0	0.1	1.6	-4.12490	0.028285499
ENSMUSG00000034584	9	Exph5	0.0	0.0	0.0	2.0	2.9	4.8	0.0	0.0	0.0	0.0	0.0	0.0	1.6	-4.11886	0.030090539
ENSMUSG00000074419	7	Gm15448	5.4	2.9	1.2	0.0	0.0	0.0	0.1	0.1	0.0	0.0	0.0	0.0	1.6	4.14848	0.032935842
ENSMUSG00000028558	4	Calr4	0.9	1.9	6.0	0.0	0.0	0.0	0.0	0.0	0.1	0.0	0.0	0.0	1.5	4.01367	0.046476263
ENSMUSG00000046215	11	Rprml	0.0	0.0	0.0	3.0	4.9	1.0	0.0	0.0	0.0	0.1	0.2	0.0	1.5	-3.98078	0.04531283
ENSMUSG00000004894	3	Hapln2	0.0	0.0	0.0	3.0	1.0	4.8	0.0	0.0	0.0	0.1	0.0	0.1	1.5	-3.97051	0.046206183
ENSMUSG00000110291	16	Gm37419	5.4	1.0	2.4	0.0	0.0	0.0	2.1	0.4	0.9	0.0	0.0	0.0	1.5	4.02518	0.044953549

Supplementary-Table S2 collagen-cytokine

geneid	chr	name	circNab1.1	circNab1.2	circNab1.3	WT1	WT2	WT3	circNab1.1_FPKM	circNab1.2_FPKM	circNab1.3_FPKM	WT1_FPKM	WT2_FPKM	WT3_FPKM	baseMean	log2FC	Pvalue
ENSMUSG00000032332	9	Col12a1	33.95	19.39	37.43	245.42	152.14	128.18	0.13	0.07	0.13	1.03	0.61	0.51	102.75	-2.542	1.04E-16
ENSMUSG00000056174	4	Col8a2	55.40	31.99	32.60	321.86	205.78	131.05	0.58	0.31	0.31	3.59	2.20	1.37	129.78	-2.445	4.56E-15
ENSMUSG00000023034	15	Nr4a1	785.41	1302.83	1206.28	604.49	434.96	405.58	13.98	21.30	19.52	11.44	7.87	7.22	789.93	1.189	3.44E-11
ENSMUSG00000021253	12	Tgfb3	485.18	431.37	423.83	1214.02	805.56	710.72	6.52	5.32	5.18	17.34	11.01	9.55	678.45	-1.025	1.22E-09
ENSMUSG00000027750	3	Postn	1400.15	1004.27	1037.23	8135.01	5073.25	3569.88	12.18	8.03	8.20	75.26	44.89	31.06	3369.97	-2.285	1.99E-09
ENSMUSG00000040690	4	Col16a1	257.34	204.54	245.12	627.63	435.94	360.62	2.25	1.64	1.95	5.83	3.87	3.15	355.20	-1.010	8.23E-08
ENSMUSG00000027966	3	Col11a1	3.57	0.00	2.41	52.30	27.31	21.04	0.02	0.00	0.01	0.33	0.16	0.12	17.77	-4.061	2.02E-07
ENSMUSG00000001131	X	Timp1	76.84	99.85	71.24	286.66	192.12	133.92	3.95	4.72	3.33	15.69	10.06	6.89	143.44	-1.301	6.32E-07
ENSMUSG00000068196	16	Col8a1	669.25	546.73	572.35	1225.08	881.63	777.68	5.95	4.47	4.63	11.59	7.98	6.92	778.79	-0.688	8.61E-06
ENSMUSG00000029661	6	Col1a2	4326.45	3202.80	3145.50	11255.04	7673.26	6388.86	36.75	25.00	24.30	101.69	66.32	54.28	5998.65	-1.246	2.18E-05
ENSMUSG00000026837	2	Col5a1	1477.89	1167.12	950.29	2076.00	1781.78	1557.28	7.99	5.80	4.67	11.93	9.80	8.42	1501.73	-0.589	5.65E-05
ENSMUSG00000015312	10	Gadd45b	175.13	188.06	195.61	306.77	283.80	251.58	6.20	6.11	6.29	11.54	10.22	8.90	233.49	-0.594	0.000136
ENSMUSG00000001506	11	Col1a1	3810.89	2606.64	2313.55	9760.40	6613.17	5301.25	29.20	18.35	16.12	79.53	51.54	40.62	5067.65	-1.312	0.000165
ENSMUSG00000019577	6	Pdk4	1408.20	2400.16	2549.01	3375.51	6326.44	7584.56	18.36	28.76	30.23	46.82	83.93	98.92	3940.65	-1.443	0.000267
ENSMUSG00000029648	5	Flt1	2978.12	3054.49	2953.51	2251.01	2378.63	2613.32	19.62	18.49	17.70	15.78	15.94	17.22	2704.85	0.311	0.000276
ENSMUSG00000022952	16	Runx1	64.33	52.35	49.51	104.60	105.33	98.53	0.50	0.37	0.35	0.86	0.83	0.76	79.11	-0.885	0.000276
ENSMUSG00000026043	1	Col3a1	9960.13	5974.23	6051.93	26170.23	17632.51	13304.79	81.33	44.82	44.94	227.28	146.47	108.66	13182.30	-1.377	0.000362
ENSMUSG00000000567	11	Sox9	90.25	74.64	83.32	224.30	130.68	114.79	0.99	0.75	0.83	2.62	1.46	1.26	119.66	-0.919	0.000394
ENSMUSG00000001119	10	Col6a1	3293.54	2494.19	2311.13	4497.99	3619.15	3182.47	36.50	25.40	23.29	53.01	40.80	35.27	3233.08	-0.480	0.000625
ENSMUSG00000000058	6	Cav2	2492.94	1976.55	2485.01	1689.76	1831.52	1911.21	41.44	30.19	37.57	29.88	30.97	31.78	2064.50	0.356	0.000901
ENSMUSG00000079022	15	Col22a1	7.15	22.30	4.83	55.32	37.06	24.87	0.04	0.11	0.02	0.30	0.19	0.13	25.25	-1.756	0.001134
ENSMUSG00000004044	11	Cavin1	9420.44	9088.82	8742.21	7595.90	7741.53	7918.40	122.60	108.68	103.46	105.14	102.49	103.07	8417.88	0.229	0.001363
ENSMUSG00000026070	1	Il18r1	12.51	8.72	8.45	39.23	33.16	19.13	0.14	0.09	0.08	0.46	0.37	0.21	20.20	-1.611	0.001547
ENSMUSG00000026042	1	Col5a2	1074.91	716.36	724.49	2303.31	1578.93	1215.79	7.37	4.51	4.52	16.80	11.02	8.34	1268.97	-1.019	0.002599
ENSMUSG00000031740	8	Mmp2	2472.39	1870.89	1671.16	4443.68	3311.95	2716.63	36.49	25.37	22.43	69.76	49.73	40.10	2747.78	-0.800	0.003193
ENSMUSG00000028626	4	Col9a2	11.62	12.60	13.28	44.26	32.18	21.04	0.18	0.18	0.19	0.73	0.51	0.33	22.50	-1.380	0.003630
ENSMUSG00000020241	10	Col6a2	3235.46	2359.45	2072.05	4070.52	3193.94	3039.95	29.93	20.05	17.43	40.04	30.05	28.12	2995.23	-0.426	0.003942
ENSMUSG00000059456	14	Ptk2b	150.11	119.23	129.20	251.45	189.20	159.75	1.69	1.23	1.32	3.01	2.17	1.80	166.49	-0.588	0.004089
ENSMUSG00000023972	17	Ptk7	146.54	131.83	107.47	223.29	188.22	161.66	1.57	1.30	1.05	2.55	2.05	1.73	159.84	-0.564	0.004200
ENSMUSG00000031565	8	Fgfr1	1032.02	766.77	835.58	1171.77	1074.72	1033.08	9.30	6.35	6.85	11.23	9.85	9.31	985.66	-0.315	0.009639
ENSMUSG00000091345	9	Col6a5	0.89	0.00	1.21	6.03	8.78	3.83	0.00	0.00	0.01	0.03	0.04	0.02	3.46	-3.194	0.023597
ENSMUSG00000002897	6	Il17ra	100.97	127.96	72.45	124.72	148.24	161.66	0.59	0.69	0.39	0.78	0.88	0.95	122.67	-0.521	0.023680
ENSMUSG00000063564	11	Col23a1	95.61	48.47	55.54	116.67	103.38	88.96	0.77	0.36	0.41	1.00	0.84	0.71	84.77	-0.621	0.026498
ENSMUSG00000030849	7	Fgfr2	13.40	15.51	9.66	36.21	22.43	20.09	0.14	0.15	0.09	0.40	0.23	0.21	19.55	-1.016	0.039420
ENSMUSG00000007613	4	Tgfbr1	428.89	356.73	444.36	559.23	456.42	467.76	3.39	2.59	3.19	4.69	3.66	3.69	452.23	-0.271	0.046950
ENSMUSG00000048126	1	Col6a3	1235.75	831.72	840.41	1875.84	1389.73	1089.52	5.56	3.44	3.44	8.97	6.36	4.90	1210.49	-0.582	0.053031
ENSMUSG00000022371	15	Col14a1	901.57	462.39	434.70	1452.39	990.85	761.42	6.34	2.99	2.78	10.87	7.09	5.36	833.89	-0.833	0.054813
ENSMUSG00000026068	1	Il18rap	4.47	7.75	3.62	12.07	10.73	17.22	0.04	0.07	0.03	0.12	0.10	0.16	9.31	-1.325	0.059107
ENSMUSG00000022969	16	Il10rb	2080.13	2439.91	2565.91	2208.76	2078.26	1962.86	33.86	36.49	37.98	38.24	34.42	31.96	2222.64	0.180	0.071272
ENSMUSG00000070427	7	Il18bp	199.26	208.41	175.09	140.81	149.21	180.79	3.49	3.35	2.79	2.62	2.66	3.16	175.60	0.310	0.081545
ENSMUSG00000038418	18	Egr1	1012.36	1037.23	992.55	1332.70	1103.01	1039.78	10.26	9.66	9.15	14.36	11.37	10.54	1086.27	-0.192	0.083288
ENSMUSG00000032089	9	Il10ra	203.72	181.27	200.44	292.69	226.26	202.79	2.65	2.16	2.37	4.04	2.99	2.63	217.86	-0.301	0.085726
ENSMUSG00000031274	X	Col4a5	653.17	562.24	654.46	740.28	716.81	669.59	4.54	3.59	4.14	5.47	5.07	4.66	666.09	-0.186	0.095643
ENSMUSG00000034807	8	Colgalt1	897.99	753.20	801.77	934.40	888.45	944.12	12.72	9.80	10.33	14.08	12.80	13.38	869.99	-0.173	0.098908

ENSMUSG00000046108	8	Il17c	0.00	0.00	1.21	2.01	0.98	5.74	0.00	0.00	0.07	0.14	0.06	0.36	1.66	-3.002	0.111581
ENSMUSG00000079465	1	Col4a3	115.26	86.27	76.07	130.76	111.18	111.92	0.61	0.42	0.37	0.74	0.60	0.59	105.24	-0.342	0.133075
ENSMUSG00000027570	2	Col9a3	2.68	11.63	9.66	13.08	11.70	21.04	0.04	0.16	0.13	0.21	0.18	0.31	11.63	-0.954	0.151024
ENSMUSG00000028197	3	Col24a1	0.89	0.97	0.00	3.02	4.88	1.91	0.01	0.01	0.00	0.02	0.03	0.01	1.94	-2.324	0.155601
ENSMUSG00000045672	4	Col27a1	228.74	163.82	129.20	253.46	211.63	179.83	1.37	0.90	0.70	1.61	1.28	1.07	194.45	-0.298	0.162615
ENSMUSG00000050222	14	Il17d	255.55	241.37	194.41	305.77	239.91	254.44	9.11	7.91	6.30	11.60	8.70	9.08	248.58	-0.206	0.213285
ENSMUSG00000043088	6	Il17re	16.08	18.42	9.66	25.15	19.50	22.00	0.25	0.26	0.14	0.41	0.31	0.34	18.47	-0.578	0.234863
ENSMUSG00000031273	X	Col4a6	65.23	50.41	41.05	77.45	60.47	62.18	0.45	0.32	0.26	0.56	0.42	0.42	59.46	-0.342	0.236014
ENSMUSG00000016529	1	Il10	0.00	0.00	1.21	1.01	2.93	1.91	0.00	0.00	0.04	0.04	0.10	0.07	1.18	-2.423	0.236072
ENSMUSG00000043719	9	Col6a6	236.78	237.50	257.19	203.17	230.16	212.36	1.23	1.13	1.21	1.12	1.21	1.10	229.53	0.178	0.237249
ENSMUSG00000025650	9	Col7a1	11.62	18.42	16.90	20.12	28.28	20.09	0.06	0.08	0.08	0.11	0.14	0.10	19.24	-0.552	0.247644
ENSMUSG00000057606	14	Colq	114.37	148.31	166.63	125.73	113.13	127.22	1.81	2.16	2.40	2.12	1.82	2.02	132.57	0.224	0.273862
ENSMUSG00000067158	1	Col4a4	293.97	248.16	276.51	311.80	322.81	274.53	1.32	1.02	1.13	1.49	1.48	1.23	287.96	-0.151	0.298485
ENSMUSG00000026147	1	Col9a1	0.00	0.97	0.00	2.01	0.00	2.87	0.00	0.01	0.00	0.03	0.00	0.03	0.98	-2.162	0.350081
ENSMUSG00000001435	10	Col18a1	538.80	401.32	329.64	488.82	458.37	461.06	4.38	2.99	2.43	4.22	3.79	3.74	446.34	-0.145	0.367671
ENSMUSG00000032312	9	Csk	737.16	668.87	617.03	713.12	743.14	705.94	12.18	10.16	9.27	12.54	12.49	11.67	697.54	-0.094	0.389773
ENSMUSG00000028599	4	Tnfrsf1b	428.00	284.99	286.17	418.42	376.45	313.75	5.09	3.12	3.10	5.30	4.56	3.73	351.30	-0.146	0.408089
ENSMUSG00000031503	8	Col4a2	6647.83	5876.33	5297.25	6584.05	6169.43	6049.28	46.83	38.03	33.93	49.33	44.21	42.62	6104.03	-0.077	0.415646
ENSMUSG00000039462	10	Col10a1	0.00	0.97	0.00	1.01	0.98	1.91	0.00	0.01	0.00	0.02	0.01	0.03	0.81	-1.838	0.424293
ENSMUSG00000039217	9	Il18	25.91	14.54	10.87	30.17	18.53	19.13	1.33	0.69	0.51	1.65	0.97	0.98	19.86	-0.383	0.443709
ENSMUSG00000028339	4	Col15a1	3784.97	2523.27	2456.03	4081.59	3169.56	2759.67	33.25	20.37	19.62	38.13	28.32	24.25	3129.18	-0.192	0.452535
ENSMUSG00000038591	15	Colec10	0.00	0.00	0.00	0.00	0.98	1.91	0.00	0.00	0.00	0.00	0.01	0.02	0.48	-2.370	0.453495
ENSMUSG00000024401	17	Tnf	23.23	17.45	8.45	25.15	22.43	17.22	0.64	0.44	0.21	0.74	0.63	0.48	18.99	-0.376	0.454233
ENSMUSG00000032649	1	Colgalt2	398.51	306.32	291.00	313.81	305.25	304.19	4.42	3.12	2.94	3.70	3.45	3.38	319.85	0.113	0.454482
ENSMUSG00000004415	5	Col26a1	24.13	18.42	4.83	26.15	22.43	14.35	0.39	0.28	0.07	0.45	0.37	0.23	18.38	-0.383	0.493265
ENSMUSG00000016356	2	Col20a1	42.00	42.65	43.47	45.26	59.49	42.09	0.38	0.36	0.36	0.44	0.55	0.38	45.83	-0.198	0.525459
ENSMUSG00000015966	14	Il17rb	14.30	3.88	7.24	8.05	4.88	5.74	0.25	0.06	0.12	0.15	0.09	0.10	7.35	0.459	0.561139
ENSMUSG00000027398	2	Il1b	32.17	33.93	28.98	48.28	24.38	37.31	1.08	1.04	0.88	1.72	0.83	1.25	34.17	-0.206	0.583857
ENSMUSG00000058806	10	Col13a1	37.53	49.44	54.34	43.25	46.81	36.35	0.54	0.65	0.71	0.66	0.68	0.52	44.62	0.154	0.629609
ENSMUSG00000058897	3	Col25a1	14.30	14.54	12.07	16.09	14.63	17.22	0.10	0.09	0.07	0.12	0.10	0.12	14.81	-0.224	0.668024
ENSMUSG00000068794	6	Col28a1	16.08	18.42	16.90	19.11	16.58	22.96	0.17	0.18	0.17	0.22	0.18	0.25	18.34	-0.191	0.686604
ENSMUSG00000021669	13	Col4a3bp	692.48	721.21	782.45	762.41	791.90	695.42	5.95	5.70	6.12	6.97	6.93	5.98	740.98	-0.036	0.735537
ENSMUSG00000004098	9	Col5a3	671.93	693.10	572.35	700.05	617.33	574.89	4.99	4.73	3.86	5.53	4.66	4.27	638.28	0.036	0.766428
ENSMUSG00000031502	8	Col4a1	10212.10	8285.21	7816.07	9423.46	8916.70	8514.34	70.14	52.29	48.82	68.84	62.30	58.49	8861.31	-0.029	0.777567
ENSMUSG00000024330	17	Col11a2	54.51	70.76	54.34	70.41	57.54	43.05	0.41	0.49	0.37	0.56	0.44	0.32	58.43	0.074	0.800577
ENSMUSG00000079559	9	Colca2	39.32	37.81	19.32	47.27	26.33	30.61	1.41	1.24	0.63	1.80	0.96	1.10	33.44	-0.097	0.810005
ENSMUSG00000040717	14	Il17rd	174.24	152.19	184.75	196.13	157.02	167.40	0.96	0.77	0.92	1.14	0.88	0.92	171.95	-0.028	0.875729
ENSMUSG00000027720	3	Il2	0.89	0.97	2.41	3.02	0.98	0.96	0.04	0.04	0.09	0.13	0.04	0.04	1.54	-0.239	0.881550
ENSMUSG00000025064	19	Col17a1	4.47	2.91	1.21	1.01	2.93	3.83	0.04	0.02	0.01	0.01	0.02	0.03	2.72	0.168	0.891626
ENSMUSG00000036103	18	Colec12	664.78	453.67	528.88	680.93	503.23	489.76	3.76	2.36	2.72	4.10	2.90	2.77	553.54	-0.022	0.894295
ENSMUSG00000030341	6	Tnfrsf1a	1472.53	1399.77	1403.10	1551.97	1403.38	1352.58	31.03	27.10	26.89	34.78	30.09	28.51	1430.55	-0.010	0.910249
ENSMUSG00000030281	6	Il17rc	283.25	266.58	222.18	277.60	269.17	236.27	5.59	4.83	3.99	5.83	5.40	4.66	259.17	-0.016	0.918417
ENSMUSG00000036655	12	Colec11	687.12	848.20	786.07	818.73	768.50	748.99	19.47	22.09	20.26	24.68	22.16	21.23	776.27	-0.010	0.929686
ENSMUSG00000000869	11	Il4	16.98	10.66	16.90	14.08	15.60	13.39	1.27	0.74	1.15	1.12	1.19	1.01	14.60	0.045	0.932358
ENSMUSG00000029287	5	Tgfbr3	881.91	850.14	1016.70	849.91	939.17	942.21	6.58	5.83	6.90	6.75	7.13	7.03	913.34	0.007	0.946364
ENSMUSG00000022483	15	Col2a1	1.79	0.97	2.41	1.01	1.95	1.91	0.02	0.01	0.02	0.01	0.02	0.02	1.67	0.069	0.963575

Table S3. Patient clinical Information

Sample number	Classification of diseases	LVEF	LVFS	AGE	SEX	Heart rhythm
40L	Normal			48	F	Normal rhythm
44L	Normal			51	M	Normal rhythm
65	Normal			1	M	Normal rhythm
61	Normal			46	M	Normal rhythm
62	Normal			37	M	Normal rhythm
68	Normal			47	M	Arrhythmia
69	Normal			32	M	Normal rhythm
70	Normal			49	M	Normal rhythm
71	Normal			57	M	Normal rhythm
74	Normal			1.5	M	Normal rhythm
77	Normal			23	M	Normal rhythm
79	Normal			42	M	Normal rhythm
94	Normal			47	F	Normal rhythm
95	Normal			24	M	Normal rhythm
104	Normal			37	F	Normal rhythm
107	Normal			61	M	Normal rhythm
119	Normal			19	M	Arrhythmia
132	Normal			34	M	Normal rhythm
137	Normal			35	M	Normal rhythm
145	Normal			50	M	Normal rhythm
146	Normal			40	M	Normal rhythm
147	Normal			55	M	Normal rhythm
153	Normal			22	F	Normal rhythm
32	HCM	70	42	65	F	AF
34	HCM	85	55	13	M	Normal rhythm
89	HCM	69	50	24	F	Normal rhythm
90	HCM	63	40	9	F	Arrhythmia
125	HCM	68	37	19	M	Normal rhythm
141	HCM	74	41	10	M	Normal rhythm
142	HCM	64	56	29	F	Arrhythmia
2	MS	61	33	61	F	Normal rhythm
28	MS	70	45	64	F	Normal rhythm
31	MS	71	42	68	F	Arrhythmia
4	AS	66	40	71	M	Normal rhythm
7	AS	60	39	53	F	Normal rhythm
27	AS	85	52	55	F	Arrhythmia
33	AS	70	31	65	F	Normal rhythm
129	AS	60	49	65	M	AF
1	TOF	65	33	6 M	M	Arrhythmia
3	TOF	64	33	11 M	F	Normal rhythm
5	TOF	70	39	7 M	F	Normal rhythm
8	TOF	75	41	11 M	M	Normal rhythm
9	TOF	70	37	1	F	Arrhythmia
11	TOF	76	43	2	F	Normal rhythm
12	TOF	73	39	7 M	M	Normal rhythm
13	TOF	84	52	3	F	Normal rhythm
14	TOF	66	34	1	M	AF
15	TOF	75	42	1	M	Normal rhythm
16	TOF	74	41	6 M	M	Normal rhythm
20	TOF	83	49	4 M	M	Normal rhythm
22	TOF	62		35	F	Normal rhythm
23	TOF	67	35	1	M	Arrhythmia
25	TOF	61		45	F	Normal rhythm
35	TOF	67	40	3 M	F	Normal rhythm
36	TOF	73	39	9 M	F	Arrhythmia
37	TOF	65	33	6 M	M	Normal rhythm
38	TOF	70		18	F	Normal rhythm
41	TOF	81	47	9 M	M	Arrhythmia
42	TOF	71	38	9 M	F	Normal rhythm
43	TOF	74	40	2 M	F	Normal rhythm
45	TOF	82	48	10 M	M	Normal rhythm
46	TOF	96	64	4 M	F	Normal rhythm

47	TOF	62	31	6 M	F	Arrhythmia
49	TOF	78	44	1	M	AF
50	TOF	75	41	1	F	Normal rhythm
52	TOF	72	38	5 M	F	Normal rhythm
54	TOF	77	44	5 M	M	Normal rhythm
55	TOF	81	47	7 M	F	Normal rhythm
56	TOF	75	41	6 M	F	Normal rhythm
57	TOF	62	32	4 M	M	Arrhythmia
58	TOF	70	38	11 M	M	Normal rhythm
59	TOF	74	41	11 M	M	Normal rhythm
60	TOF	84	50	5 M	M	Normal rhythm
63	TOF	61	31	5M	M	Arrhythmia
64	TOF	68	37	1	M	Normal rhythm
6	TOF	66	34	7 M	M	Normal rhythm
66	TOF	63	32	3	M	Normal rhythm
72	TOF	69		23	F	Arrhythmia
73	TOF	61	30	9 M	M	Normal rhythm
75	TOF	63	32	1	F	Arrhythmia
76	TOF	77	44	6 M	M	Normal rhythm
78	TOF	71	40	7 M	M	Arrhythmia
80	TOF	73	42	6M	F	AF
81	TOF	70	46	2	M	Normal rhythm
82	TOF	66	33	1 M	M	Normal rhythm
83	TOF	75	40	8 M	F	AF
84	TOF	88	57	2	F	Arrhythmia
86	TOF	66	35	7 M	M	Normal rhythm
87	TOF	75	41	6 M	F	Arrhythmia
88	TOF	69	35	7 M	M	Normal rhythm
91	TOF	68	36	6 M	F	Normal rhythm
92	TOF	66	33	7 M	F	Normal rhythm
93	TOF	67	34	1	M	Normal rhythm
96	TOF	65	35	5	M	Arrhythmia
99	TOF	61	32	15	M	AF
108	TOF	56		43	F	Normal rhythm
109	TOF	76	44	5	F	Normal rhythm
110	TOF	67	35	6 M	M	Normal rhythm
111	TOF	63	32	6 M	M	Normal rhythm
113	TOF	79	45	5 M	M	AF
115	TOF	77	43	3	F	Normal rhythm
116	TOF	82	49	4	M	Normal rhythm
117	TOF	81	46	1	F	Normal rhythm
118	TOF	71	44	8 M	M	Normal rhythm
100	TOF	82	48	9 M	M	Arrhythmia
101	TOF	80	46	7 M	M	Normal rhythm
103	TOF	65		20	M	Normal rhythm
105	TOF	70	38	6	M	Normal rhythm
106	TOF	64		20	F	Normal rhythm
120	TOF	66		52	F	Arrhythmia
121	TOF	75	41	8 M	M	Normal rhythm
122	TOF	74	40	1	M	Normal rhythm
124	TOF	81	46	1	F	Normal rhythm
126	TOF	80	50	8 M	M	Arrhythmia
127	TOF	82	49	1	M	Normal rhythm
128	TOF	67	34	5	M	Normal rhythm
130	TOF	76	42	5 M	M	Normal rhythm
131	TOF	63	32	10 M	M	AF
133	TOF	80	45	M	M	Normal rhythm
135	TOF	78	44	2	M	Normal rhythm
136	TOF	70	37	6 M	M	Arrhythmia
138	TOF	63		27	F	Normal rhythm
140	TOF	81	46		M	Normal rhythm
143	TOF	81	47	6 M	M	Arrhythmia
144	TOF	71	48	5 M	M	Normal rhythm
148	TOF	66	35	6 M	F	AF
149	TOF	64	32	2	M	Normal rhythm

151	TOF	62	42	27	F	Normal rhythm
152	TOF	71	44	6 M	F	AF
HF9	HF	36		66	M	Normal rhythm
HF10	HF	26.3	12.8	12	M	AF
11L	HF	23		50	M	Normal rhythm
12L	HF	37		54	M	AF
13L	HF	23		64	M	Arrhythmia
14L	HF	29		37	M	AF
15L	HF	32		52	M	Normal rhythm
16HF	HF	44		41	M	Normal rhythm
17 HF	HF	24		29	F	AF
18HF	HF	23		51	F	Normal rhythm
19HF	HF	16.9		45	M	Normal rhythm
20HF	HF	24		52	F	Arrhythmia
HF21	HF	26		55	M	Normal rhythm
HF22	HF	31		59	M	Arrhythmia
HF23	HF	27		60	M	Normal rhythm
HF24	HF	18	7	61	F	Normal rhythm
HF25	HF	28	14	40	M	AF
HF26	HF	20		23	M	Normal rhythm
HF27	HF	24		49	M	Normal rhythm
HF28	HF	25	12	11	F	Normal rhythm
HF29	HF	9		64	M	Arrhythmia
HF31	HF	24		31	M	Normal rhythm
HF32	HF	21		68	M	Normal rhythm
HF33	HF	33		28	M	Arrhythmia
HF34	HF	19		38	F	Normal rhythm
HF35	HF	27		53	M	AF
HF2	HF	27		23	M	Normal rhythm
HF3	HF	21		55	M	Normal rhythm
HF4	HF	27		44	M	AF
HF5	HF	22		42	M	Arrhythmia
HF6	HF	27		54	M	Normal rhythm
HF7	HF	28		55	M	AF
HF8	HF	14		54	M	Normal rhythm

Normal: Normal heart (n=23)
HCM: Hypertrophic cardiomyopathy (n=7)
MS: Mitral stenosis (n=3)
AS: Aortic stenosis (n=5)
TOF: Tetralogy of Fallot (n=91)
HF: Heart failure (n=33)
LVEF: Left ventricular ejection fraction
LVFS: Left ventricular fractional shortening
AF: Atrial fibrillation

Major Resources Table

In order to allow validation and replication of experiments, all essential research materials listed in the Methods should be included in the Major Resources Table below. Authors are encouraged to use public repositories for protocols, data, code, and other materials and provide persistent identifiers and/or links to repositories when available. Authors may add or delete rows as needed.

Animals (in vivo studies)

Species	Vendor or Source	Background Strain	Sex	Persistent ID / URL
Mouse	Inhouse breeding	C57BL/6J	Male and female	

Genetically Modified Animals

	Species	Vendor or Source	Background Strain	Other Information	Persistent ID / URL
**Parent - Male**	Mouse	Toronto Centre for Phenogenomics (TCP)	C57BL/6J	circNAB1 transgenic mice	http://phenogenomics.ca/index2.html?
**Parent - Male**	Mouse	Jackson	C57BL/6J	B6.FVB-Tg (Myh6-cre) 2182Mds/J	https://www.jax.org/strain/011038
**Parent - Male**	Mouse	Jackson	C57BL/6J	$Stk11^{tm1.1Sjm}$/J	https://www.jax.org/strain/014143
**Parent - Female**	Mouse	Jackson	C57BL/6J	B6.FVB-Tg (Myh6-cre) 2182Mds/J	https://www.jax.org/strain/011038
**Parent - Female**	Mouse	Jackson	C57BL/6J	$Stk11^{tm1.1Sjm}$/J	https://www.jax.org/strain/014143

Antibodies

Target antigen	Vendor or Source	Catalog #	Working concentration	Lot # (preferred but not required)	Persistent ID / URL
NAB1-356	Genscript	Custom-made	WB:1000, IP: 30 µl each IF: 1:200		
NAB1 (N-T)	Novus Bio	NBP1-86163	WB:500 IP: 10 µl each IF: 1:100		https://www.novusbio.com/products/nab1-antibody_nbp1-86163
NAB1 (N-T)	Novus Bio	NBP2-55767	IF: 1:100		https://www.novusbio.com/products/mypop-antibody_nbp2-55767
NAB1 (C-T)	Santa Cruz	SC-137084	WB: 1:2000, IP: 10 µl each		https://www.scbt.com/p/nab1-antibody-a-8
EGR1	Santa Cruz	SC-515830	WB: 1:5000, IP: 5 µl each IF: 1:200		https://www.scbt.com/p/egr-1-antibody-b-6
Gadd45B	Santa Cruz	SC-377311	WB: 1:1000, IP: 10 µl each IF: 1:200		https://www.scbt.com/p/gadd-45beta-antibody-g-11

DOI [to be added]

Runx1	ABclonal	A2055	WB: 1:4000,<br>IP: 5 µl each<br>IF: 1:200		https://abclonal.com/catalog-antibodies/RUNX1RabbitpAb/A2055
LKB1	ABclonal	A22636	WB: 1:4000,<br>IP: 5 µl each<br>IF: 1:200		https://abclonal.com/index.php?s=/catalog-antibodies/KOValidatedLKB1RabbitmAb/A22636
cardiac troponin T	Abcam	Ab8295	IF: 1:500		https://www.abcam.com/cardiac-troponin-t-antibody-1c11-ab8295.html
vimentin	Cell signaling	5741	IF: 1:500		https://www.cellsignal.com/products/primary-antibodies/vimentin-d21h3-xp-rabbit-mab/5741
goat anti-mouse HRP	Bio-Rad	1706516	WB: 1:10000		https://www.bio-rad.com/en-ca/sku/1706516-goat-anti-mouse-igg-h-l-hrp-conjugate?ID=1706516
goat anti-rabbit HRP	Bio-Rad	1706515	WB: 1:10000		https://www.bio-rad.com/en-ca/sku/1706515-goat-anti-rabbit-igg-h-l-hrp-conjugate?ID=1706515
Goat anti-Mouse IgG, Alexa Fluor 488	ThemoFisher Scientific	A-11001	IF: 1:200		https://www.thermofisher.com/antibody/product/Goat-anti-Mouse-IgG-H-L-Cross-Adsorbed-Secondary-Antibody-Polyclonal/A-11001
Goat anti-Mouse IgG, Alexa Fluor Plus 647	ThemoFisher Scientific	A-32728	IF: 1:200		https://www.fishersci.ca/shop/products/goat-anti-mouse-igg-h-l-secondary-antibody-alexa-fluor-plus-647-invitrogen/pia32728?searchHijack=true&searchTerm=PIA32728&searchType=RAPID&matchedCatNo=PIA32728
Goat anti-Rabbit IgG, Alexa Fluor 555	ThemoFisher Scientific	A-21422	IF: 1:200		https://www.fishersci.ca/shop/products/anti-mouse-igg-h-l-alexa-fluor-555-conjugated-polyclonal-thermo-scientific-novex-4/a21422?searchHijack=true&searchTerm=A21422&searchType=RAPID&matchedCatNo=A21422
Goat anti-Rabbit IgG, Alexa Fluor 488	ThemoFisher Scientific	A-32731	IF: 1:200		https://www.fishersci.ca/ca/en/catalog/search/products?keyword=A-32731
Goat anti-Rabbit IgG, Alexa Fluor Plus 647	ThemoFisher Scientific	A-32732	IF: 1:200		https://www.fishersci.ca/shop/products/goat-anti-rabbit-igg-h-l-secondary-antibody-alexa-fluor-plus-555-invitrogen/pia32732?searchHijack=true&searchTerm=PIA32732&searchType=RAPID&matchedCatNo=PIA32732
Goat anti-Rabbit IgG, Alexa Fluor 555	ThemoFisher Scientific	A-32733	IF: 1:200		https://www.fishersci.ca/ca/en/catalog/search/products?keyword=A-32733

DNA/cDNA Clones

DOI [to be added]

Clone Name	Sequence	Source / Repository	Persistent ID / URL
circNAB1		Gene Universal	
NAB1-356		Gene Universal	
circNAB1-mut		Gene Universal	
circNAB1 precursor		Gene Universal	

Primers

Primer Name	Sequence	Source / Repository	Persistent ID / URL
circNAB1 genotying-1F	5' gcactttctgcatgtcccccgtc	Eurofins Genomics LLC	
circNAB1 genotying-1R	5' cggcagaagagagaaccagtgag	Eurofins Genomics LLC	
circNAB1 genotying-2F	5' gcactttctgcatgtcccccgtc	Eurofins Genomics LLC	
circNAB1 genotying-2R	5' cggcagaagagagaaccagtgag	Eurofins Genomics LLC	
humu.circNAB1-mRNA-F1	5' cccaaagagaattaaagtggag	Eurofins Genomics LLC	
humu.circNAB1-mRNA-R1	5' gtgaactaagagctgcaagttc	Eurofins Genomics LLC	
mu.circNAB1-R	5' ggtaaggctgtggccattactg	Eurofins Genomics LLC	
mu.circNAB1-F	5' cgatttgactccaaaagaaagg	Eurofins Genomics LLC	
hu.circNAB1-R	5' cagggtcctgggtaaggccgca	Eurofins Genomics LLC	
hu.circNAB1-F	5' cccaaagagaattaaagtggag	Eurofins Genomics LLC	
humu.EGR1-F	5' gcctgggcaccccagaccagaag	Eurofins Genomics LLC	
humu.EGR1-R	5' gtcctgggagcccgactgagtg	Eurofins Genomics LLC	
humu.Runx1-F	5' cgccttccaccgcgctgagc	Eurofins Genomics LLC	
humu.Runx1-F	5' catgctgcggtcgccgctcc	Eurofins Genomics LLC	
humu.Gadd45B-F	5' gacagcgtggtcctctgcctc	Eurofins Genomics LLC	
humu.Gadd45B-R	5' cgatgttgatgtcgttgtcac	Eurofins Genomics LLC	
hu.FN1-F	5' acaagtgtctctctaccaaggtc	Eurofins Genomics LLC	
hu.FN1-R	5' ctgcatacaaagtgtcttcaatac	Eurofins Genomics LLC	
mu.FN1-F	5' GAAGACAGATGAGCTTCCCCA	Eurofins Genomics LLC	
mu.FN1-R	5' GGTTGGTGATGAAGGGGGTC	Eurofins Genomics LLC	
hu.VIM-F	5' taaaaattgcacacacttggtgc	Eurofins Genomics LLC	
hu.VIM-R	5' ctatcttgcgctcctgaaaaactgc	Eurofins Genomics LLC	
mu.VIM-F	5' taaaaattgcacacacttggtgc	Eurofins Genomics LLC	
mu.VIM-R	5' ct at ctt gcgc tcct gaaaaa ct gc	Eurofins Genomics LLC	
hu.Coll-Ia1F	5' taaactccctccatcccaacctgg	Eurofins Genomics LLC	
hu.Coll-Ia1R	5' tcgtggcccttcctgactctcctccg	Eurofins Genomics LLC	
hu.mu.coll-IIIA1-F	5' gtaaagaagtctctgaagctgatg	Eurofins Genomics LLC	
hu.mu.coll-IIIA1-R	5' gcgatatctatgatgggtagtctc	Eurofins Genomics LLC	
mu.coll-IA1-F	5' gaatggagatgatggggaagctgg	Eurofins Genomics LLC	
mu.coll-IA1-R	5' catctcctttggcaccatccaaac	Eurofins Genomics LLC	
hu.GAPDH-F	5' aaggctgggg ctcatttgca g	Eurofins Genomics LLC	
hu.GAPDH-R	5' gat gtt ctg gag agc ccc gcg	Eurofins Genomics LLC	

DOI [to be added]

hu.mu.U6-F	5’ gtgctcgcttcggcagcacata	Eurofins Genomics LLC	
hu.mu.U6-F	5’ tggaacgcttcacgaatttgcg	Eurofins Genomics LLC	
Myh6-cre genotying-F	5’ atgacagacagatccctcctatctcc	Eurofins Genomics LLC	https://www.jax.org/Protocol?stockNumber=011038&protocolID=23633
Myh6-cre genotying-R	5’ ctcatcactcgttgcatcatcgac	Eurofins Genomics LLC	https://www.jax.org/Protocol?stockNumber=011038&protocolID=23633
Myh6-cre genotying Internal positive control-F	5’ caaatgttgcttgtctggtg	Eurofins Genomics LLC	https://www.jax.org/Protocol?stockNumber=011038&protocolID=23633
Myh6-cre genotying Internal positive control-R	5’ gtcagtcgagtgcacagttt	Eurofins Genomics LLC	https://www.jax.org/Protocol?stockNumber=011038&protocolID=23633
Stk11$^{tm1.1Sjm}$/J genotying-F	5’ atcggaatgtgatccagctt	Eurofins Genomics LLC	https://www.jax.org/Protocol?stockNumber=014143&protocolID=29002
Stk11$^{tm1.1Sjm}$/J genotying-R	5’ cgtaggctgtgcaacctct	Eurofins Genomics LLC	https://www.jax.org/Protocol?stockNumber=014143&protocolID=29002

Primers labeled with “hu” were used for human sequences. Primers labeled with “mu” were used for mouse sequences. Primers labeled with “hu,mu” were used for human and mouse sequences.

siRNAs

siRNA Name	Sequence	Source / Repository	Persistent ID / URL
hu.cirNAB1-1	5’ gagaauuaaaguggagguu	Gene Universal	
hu.cirNAB1-2	5’ gaauuaaaguggagguuaa	Gene Universal	
hu.NAB1-1	5’ cguuccaggauaucuugaa	Gene Universal	
hu.NAB1-2	5’ gcccgggaaccucauuuaa	Gene Universal	
mu.EGR1-1	5’ Ccagccgcaugcgcaagua	Gene Universal	
mu.EGR1-2	5’ gccauguccaaguucuuca	Gene Universal	
mu.Runx1-1	5’ CGGGCAUCGGCAUCGGCAU	Gene Universal	
mu.Runx1-2	5’ GGAGCGGCGACCGCAGCAU	Gene Universal	
mu.Gadd45B-1	5’ GGCUUGGUGGAGGUGGCCA	Gene Universal	
mu.Gadd45B-2	5’ GGGUCCCCUAUAUCUCUCU	Gene Universal	

siRNAs labeled with “hu” were used to silence human sequences. siRNAs labeled with “mu” were used to silence mouse sequences. siRNAs labeled with “hu,mu” were used to silence human and mouse sequences.

DOI [to be added]

Cultured Cells

Name	Vendor or Source	Sex (F, M, or unknown)	Persistent ID / URL
Primary mouse cardiomyocytes (wt and circNAB1)	Isolated in the lab	Male and female	
Primary moue cardiac fibroblasts (wt and circNAB1)	Isolated in the lab	Male and female	
HL-1 atrial myocytes, mouse	Dr. William C. Claycomb; LSUHSC School of Medicine- Derived from AT-1 subcutaneous tumor from an adult female mouse, Jackson Laboratory C57BL/6J.	Female	
AC16 cardiomyocytes, human	Millipore Sigma	unknown	
MCF cardiac fibroblasts, mouse	Isolated in the lab	Male and female	

Other

Description	Source / Repository	Catalog #	Persistent ID / URL
Presto™ Mini Plasmid Kit	Geneaid	PD300	https://www.geneaid.com/Plasmid-DNA-Purification/PDH
GENEzol™ TriRNA Pure Kit	Geneaid	GZXD20 0	https://www.geneaid.com/Tri-RNA/GZXD
iScript™ Reverse Transcription Supermix for RT-qPCR	Bio-Rad	170884 0	https://www.bio-rad.com/en-ca/product/iscript-reverse-transcription-supermix-for-rt-qpcr?ID=M87EVMKG4
iTaq Universal SYBR Green Supermix	Bio-Rad	172512 0	https://www.bio-rad.com/en-ca/product/itaq-universal-sybr-green-supermix?ID=M87FTF8UU
IPTG	Duchefa Biochemie	367-93-1	https://www.duchefa-biochemie.com/product/details/number/I1401
NucBlue™ Fixed Cell ReadyProbes™ Reagent (DAPI)	ThemoFisher Scientific	R37606	https://www.thermofisher.com/order/catalog/product/R37606#/R37606
SureBeads™ Protein G Magnetic Beads	Bio-Rad	161402 3	https://www.bio-rad.com/en-ca/sku/1614023-surebeads-protein-g-magnetic-beads-3-ml?ID=1614023
Western Blotting Reagents	Millipore Sigma	C72652	https://www.emdmillipore.com/CA/en/product/Western-Blotting-Reagents,MM_NF-C72652
SimpleChIP® Enzymatic Chromatin IP Kit	Cell Signaling	9002	https://www.cellsignal.com/products/chip-kits-reagents/enzymatic-chromatin-ip-kit-agarose-beads/9002

DOI [to be added]

Masson's Trichrome Stain Kit	American MasterTech	ML7227	https://www.marketlab.com/masson-2000-trichrome-stain-kit/p/Masson2000TrichromeStainKit/
Sirius Red	Millipore Sigma	365548	https://www.sigmaaldrich.com/catalog/product/sial/365548?lang=en&region=CA&gclid=Cj0KCQiA-aGCBhCwARIsAHDl5x-s26o7QXMURYtIJKIdwTu3oZjhtsmDZyfTSURxRZE4Z16EvqaiXocaArjzEALw_wcB
Pierce™ Crosslink Magnetic IP/Co-IP Kit	ThemoFisher Scientific	88805	https://www.thermofisher.com/order/catalog/product/88805?ca&en#/88805?ca&en
VECTASTAIN® ABC Kits	Vector Laboratories	PK-6100	https://vectorlabs.com/vectastain-elite-abc-kit-standard.html
DAB Substrate Kit	Vector Laboratories	SK-4100	https://vectorlabs.com/dab-peroxidase-hrp-substrate.html
Hematoxylin Solution, Mayer's	Millipore Sigma	MHS32	https://www.sigmaaldrich.com/catalog/product/sigma/mhs32?lang=en&region=CA
Alexa Fluor™ 488 Phalloidin	ThemoFisher Scientific	A12379	https://www.thermofisher.com/order/catalog/product/A12379?ca&en#/A12379?ca&en
Alexa Fluor™ 555 Phalloidin	ThemoFisher Scientific	A34055	https://www.thermofisher.com/order/catalog/product/A34055?SID=srch-srp-A34055#/A34055?SID=srch-srp-A34055
eBioscience™ Annexin V Apoptosis Detection Kit APC	ThemoFisher Scientific	88-8007-72	https://www.thermofisher.com/order/catalog/product/88-8007-72?ca&en#/88-8007-72?ca&en
Propidium iodide (PI)	Millipore Sigma	P4170	https://www.sigmaaldrich.com/catalog/product/sigma/p4170?lang=en&region=CA
RNAse R	abm	E049	https://www.abmgood.com/rnase-r-e049.html
Carbamylcholine	Millipore Sigma	51-83-2	https://www.sigmaaldrich.com/CA/en/search/carbachol?focus=products&page=1&perpage=30&sort=relevance&term=carbachol&type=product_name
mPEG-SH	Nanocs	PG1-TH-2k-1	https://www.nanocs.net/mPEG-Thiol-2k-1g.htm
AuNP	Cytodiagnostics	CG-10-20	https://www.cytodiagnostics.com/products/10nm-stabilized-gold-nanoparticles?variant=31504342253642

DOI [to be added]
